# Discovery of indolylchalcone benzenesulfonamides as selective inhibitors of tumor-associated carbonic anhydrase IX and XII

**DOI:** 10.1038/s41598-026-49231-6

**Published:** 2026-07-08

**Authors:** Joohan Lee, Karol Biernacki, Simone Giovannuzzi, Hossam Nada, Shubham C Rivonker, Changseong Kim, Claudiu T. Supuran, Ahmed Elkamhawy, Kyeong Lee

**Affiliations:** 1 College of Pharmacy, Dongguk University-Seoul, Goyang, 10326 Republic of Korea; 2 Section of Pharmaceutical and Nutraceutical Sciences, Department of NEUROFARBA, University of Florence, Polo Scientifico, Via U. Schiff 6, Sesto Fiorentino, 50019 Florence, Italy; 3 Department of Organic Chemistry, Faculty of Chemistry, Gdańsk University of Technology, Narutowicza St. 11/12, 80-233 Gdańsk, Poland; 4 Department of Radiology, Molecular Imaging Innovations Institute (MI3), Weill Cornell Medicine, New York, NY 10065 USA; 5 Department of Chemistry, School of Sciences and Humanities, Nazarbayev University, Kabanbay Batyr Avenue 53, 010000 Astana, Kazakhstan; 6 Department of Pharmaceutical Organic Chemistry, Faculty of Pharmacy, Mansoura University, Mansoura, 35516 Egypt

**Keywords:** Biochemistry, Cancer, Chemical biology, Chemistry, Computational biology and bioinformatics, Drug discovery

## Abstract

Indole, characterized by its favorable bioavailability, unique structural attributes, and a broad pharmacological profile, represents a privileged scaffold in anticancer drug discovery. Chalcones constitute another important scaffold in medicinal chemistry and numerous chalcone derivatives have been reported to exhibit diverse biological activities, including anticancer and enzyme inhibitory effects. Several chalcone-based compounds have also been investigated as carbonic anhydrase inhibitors. Sulfonamides are a classical class of carbonic anhydrase (CA, EC 4.2.1.1) inhibitors with diverse therapeutic relevance, being employed as diuretics, anticonvulsants, topical antiglaucoma agents and in the management of obesity and cancer. Herein, we designed and synthesized a novel series of indolylchalcone–benzenesulfonamide hybrids (**15a**−**m** and **16a**−**d**) and evaluated their inhibitory activities against a panel of four human carbonic anhydrases (hCA isoforms I, II, IX and XII). Interestingly, most of the tested compounds inhibited the tumor-associated hCA IX and XII isoforms with single- to double-digit nanomolar inhibition constants (*K*_i_s). In particular, compound **15h** ((*E*)-4-(3-(5-cyano-1*H*-indol-3-yl)acryloyl)benzenesulfonamide) showed potent inhibition of hCA IX (*K*_i_ = 8.9 nM) with marked selectivity over hCA I, II, and XII (SI = 61.9, 7.2, and 5.7, respectively), whereas compound **15j** ((*E*)-4-(3-(6-bromo-1*H*-indol-3-yl)acryloyl)benzenesulfonamide) exhibited strong inhibition of hCA XII (*K*_i_ = 4.9 nM) with pronounced selectivity over hCA I, II, and IX (SI = 628.4, 12.8, and 11.9, respectively), compared with the reference inhibitor acetazolamide (AAZ). Structure–activity relationship analysis revealed that electron withdrawing groups, particularly cyano and bromo substituents on the indole scaffold, conferred enhanced selectivity toward tumor-associated carbonic anhydrase isoforms. Molecular docking demonstrated that both compounds adopted the canonical sulfonamide-binding mode, coordinating with Zn²⁺ and engaging in stable hydrogen-bond interactions with key active-site residues. Complementary molecular dynamics simulations confirmed the persistence of these interactions, as reflected by lower RMSD values and the maintenance of critical contacts throughout the simulation period. In addition, in silico ADME prediction using the SwissADME server suggested that compound **15j** possesses a more favorable balance of lipophilicity and polarity, indicating improved membrane permeability and oral absorption potential compared to **15h** and acetazolamide (AAZ), whereas **15h** exhibited relatively higher polarity that may limit permeability despite comparable lipophilicity. Accordingly, these findings suggest that the novel indolylchalcone-benzenesulfonamide hybrids **15h** and **15j** represent promising leads for the development of potent and selective inhibitors of tumor-associated carbonic anhydrases.

**Supplementary Information:**

The online version contains supplementary material available at 10.1038/s41598-026-49231-6.

## Introduction

Carbonic anhydrases (CAs) are zinc-containing metalloenzymes that catalyze the reversible hydration of carbon dioxide (CO_2_) to bicarbonate ions (HCO_3_^−^) and protons (H^+^)^1,2^. These enzymes are essential for maintaining physiological homeostasis, playing pivotal roles in regulating acid-base balance, ion transport, gas exchange and various metabolic pathways^3^. Human carbonic anhydrases (hCAs) belong to the α-class and they are classified into fifteen isoforms which exhibit distinct cellular localization, structural characteristics, tissue-specific distribution and catalytic kinetics^4,5^. These isoforms can be grouped into catalytically active cytosolic isoforms (hCA I, II, III, VII, and XIII), membrane-associated isoforms (hCA IV, IX, XII, and XIV), mitochondrial isoforms (hCA VA and VB) and secreted isoforms (hCA VI)^6–8^, as well as the catalytically inactive carbonic anhydrase-related proteins (hCA VIII, X, and XI)^9^. Due to their diverse biological functions, CAs were found to be attractive therapeutic targets with promising clinical applications. 

Carbonic anhydrase inhibitors (CAIs) are a key therapeutic class with clinical applications for conditions such as glaucoma^10^, epilepsy^11^, altitude sickness^12^ and idiopathic intracranial hypertension^13^. Recently, CAIs have been explored as therapeutic agents in oncology, primarily due to their potential in selectively inhibiting tumor-associated isoforms, particularly hCA IX and XII^4,14^. Human CA IX and XII isoforms have been shown to be overexpressed in hypoxic tumor microenvironments which supports tumor cell survival, growth and metastasis. Classical CAIs such as acetazolamide (**1**), methazolamide (**2**), and ethoxzolamide (**3**) are widely used clinically but lack isoform selectivity, whereas SLC-0111 (**4**) has emerged as a more selective inhibitor targeting tumor-associated hCA IX. To date, benzenesulfonamides have been one of the most common class capable of inhibiting hCA with favorable selectivity toward tumor-associated isoforms where their activities extend to single-digit nanomolar *K*_i_ values^4,14,15,16,17^. Among the benzenesulfonamides, carbohydrate-based triazoles have frequently shown potent inhibition of hCA IX and XII, suppression of hCA IX-dependent tumor cell viability in cell-based models, and effective mimicry of hCA IX and XII gene silencing^18^. Glycosyl coumarin CAIs are another class that has demonstrated strong antitumor efficacy in vivo by suppressing the growth and metastasis of hypoxic CA IX-positive breast tumors, while exerting minimal effects on CA IX-negative tumors^19^. While the current CAIs have shown promising efficacy, there are several limitations such as physicochemical properties, incomplete isoform selectivity and reduced performance under hypoxic conditions. These limitations highlight the need for novel CAIs with improved drug-like properties and reliable hCA IX and XII selectivity. It is also worthy to mention that other isoforms such as hCA I and hCA II are ubiquitously expressed; while hCA I predominantly mediates physiological processes, hCA II is implicated in various cancers including melanoma^20^, renal^21^, lung^22^ and esophageal cancers^23^, as well as angiogenesis modulation via vascular endothelial growth factor receptor (VEGFR) signaling pathways^24^.

A major challenge in developing CAIs is the lack of isoform selectivity, which often results in off-target interactions^25^. Since many hCA isoforms are widely expressed in normal tissues, non-selective inhibition may interfere with physiological functions such as acid-base balance, electrolyte transport, and respiration^3,26^. Thus, the development of new inhibitors with improved selectivity toward tumor-associated isoforms, particularly hCA IX and XII, has become an important strategy for minimizing adverse effects and improving therapeutic outcomes^27^. To address this issue, current strategies focus on enhancing isoform selectivity and inhibitory potency by modifying the zinc-binding group (ZBG) or incorporating specific functional groups onto established pharmacophoric scaffolds^28^. Sulfonamides have emerged as the predominant ZBG due to their strong coordination to the catalytic zinc ion (Zn^2+^) in the enzyme’s active site. The negatively charged nitrogen atom within the sulfonamide moiety (SO_2_NH_2_) interacts strongly with the positively charged zinc ion, resulting in potent inhibitory activity^29^.

**Fig. 1:**
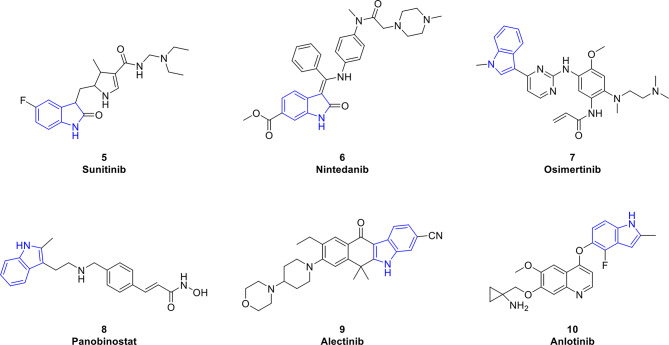
Chemical structures of FDA-approved anticancer agents containing indole moiety.

Indole is a promising scaffold for anticancer drug research due to its bioavailability, unique chemical properties and significant pharmacological activities (Fig. 1)^30^. Several FDA-approved anticancer agents contain indole moiety and act as histone deacetylase (HDAC) inhibitors or tyrosine kinase inhibitors (TKIs). Sunitinib (Sutent^®^, **5**), an indolinone-based TKI, inhibits VEGFRs, PDGFRs, c-KIT, FLT3 and RET kinases, making it a first-line treatment for renal cell carcinoma (RCC), though resistance often develops^31^. Nintedanib (Ofev^®^, **6**), another indolinone-derived TKI, targets FGFR, VEGFR and PDGFR and was FDA-approved in 2014 for idiopathic pulmonary fibrosis (IPF), with ongoing cancer trials^32^. Osimertinib (Tagrisso^®^, **7**), a third-generation EGFR TKI, was approved in 2015 for EGFR-mutant non-small-cell lung cancer (NSCLC)^33^. Panobinostat (Farydak^®^, **8**), an HDAC inhibitor, was approved in 2015 for multiple myeloma and later designated as an orphan drug for glial tumors in 2022^31^. Alectinib (Alecensa^®^, **9**), a second-generation ALK inhibitor, was approved in 2015 for ALK-positive NSCLC, overcoming resistance to crizotinib^34^. Anlotinib (Focus V^®^, **10**), a multi-target RTKI, was approved in China in 2018 for advanced/metastatic NSCLC and later designated as an orphan drug in Europe for soft tissue sarcoma, with ongoing phase II and III trials for various cancers^35^.

Beside indole-containing pharmacophores, chalcones represent another privileged scaffold in medicinal chemistry and have attracted considerable attention due to their simple synthetic accessibility and diverse pharmacological properties. Numerous chalcone derivatives have been reported to exhibit a wide range of biological activities, including anticancer, antimicrobial, anti-inflammatory, and enzyme inhibitory effects. In particular, chalcone-based benzenesulfonamides have also been reported as potent inhibitors of human carbonic anhydrases, demonstrating that the chalcone framework can be successfully combined with sulfonamide zinc-binding group to produce effective CAIs^36^. These findings highlight the suitability of chalcone scaffolds for the development of sulfonamide-containing CAIs.

In addition to the aforementioned chalcone-sulfonamide hybrids, there have been increasing reports of more complex hybrid molecules incorporating indole and chalcone scaffolds. For example, indole-chalcone-sulfonamide hybrids have been reported to exhibit potent therapeutic effects in cancer. Similarly, fluorinated indole-based chalcones and their benzenesulfonamide analogues have been synthesized and evaluated for diverse pharmacological properties which further supports key therapeutic roles exhibited by indole-chalcone-sulfonamide hybrid structures^37,38^. Together, these reports highlight the potential of combining indole, chalcone, and sulfonamide pharmacophores within a single molecular framework.

**Fig. 2:**
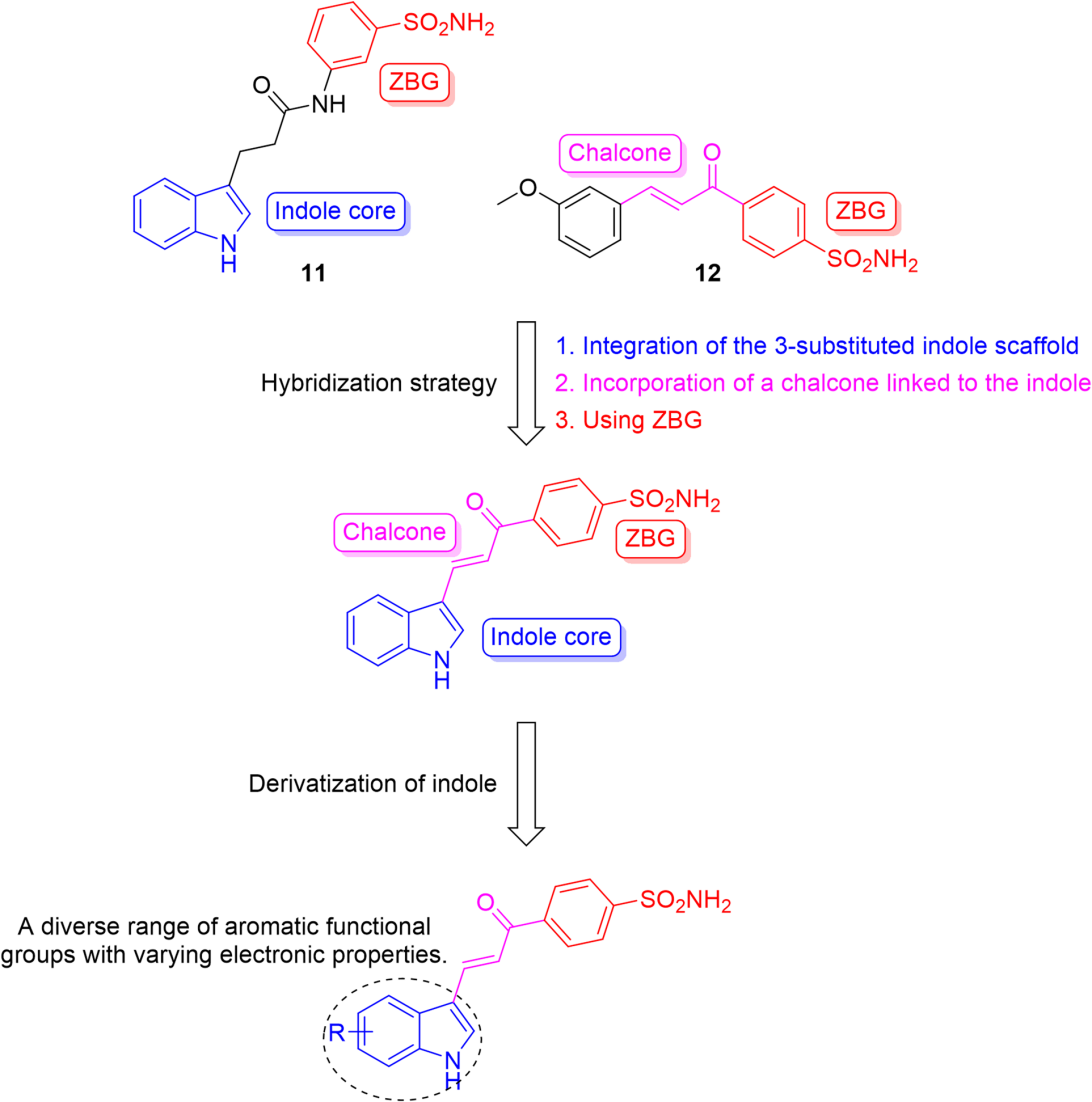
Design strategy of the novel indolylchalcone-benzenesulfonamides.

To overcome the limitations of current CAIs, our research group has previously designed and synthesized different novel series of structurally diverse hCAIs^36,39,40^. Among these compounds, the indole-based inhibitor **11** and the chalcone-containing inhibitor **12** demonstrated notable potency^36,40^. Encouraged by these findings, we further developed an innovative series of hybrid indole-chalcone derivatives incorporating a benzenesulfonamide moiety as a critical ZBG (Fig. 2). This hybridization strategy integrates the pharmacophores of both indole and chalcone, aiming to enhance potency and selectivity simultaneously. Additionally, systematic derivatization of the indole scaffold was conducted by introducing diverse substituents with varying electronic properties to elucidate structure-activity relationships (SAR) (Fig. 2). The synthesized derivatives were structurally characterized and evaluated for inhibitory activity against four hCA isoforms (hCA I, II, IX and XII). Molecular docking and molecular dynamics studies were further performed to understand the binding interaction and elucidate the inhibitory mechanisms of these novel derivatives.

## Results and discussion

### Chemical synthesis

**Scheme 1:**
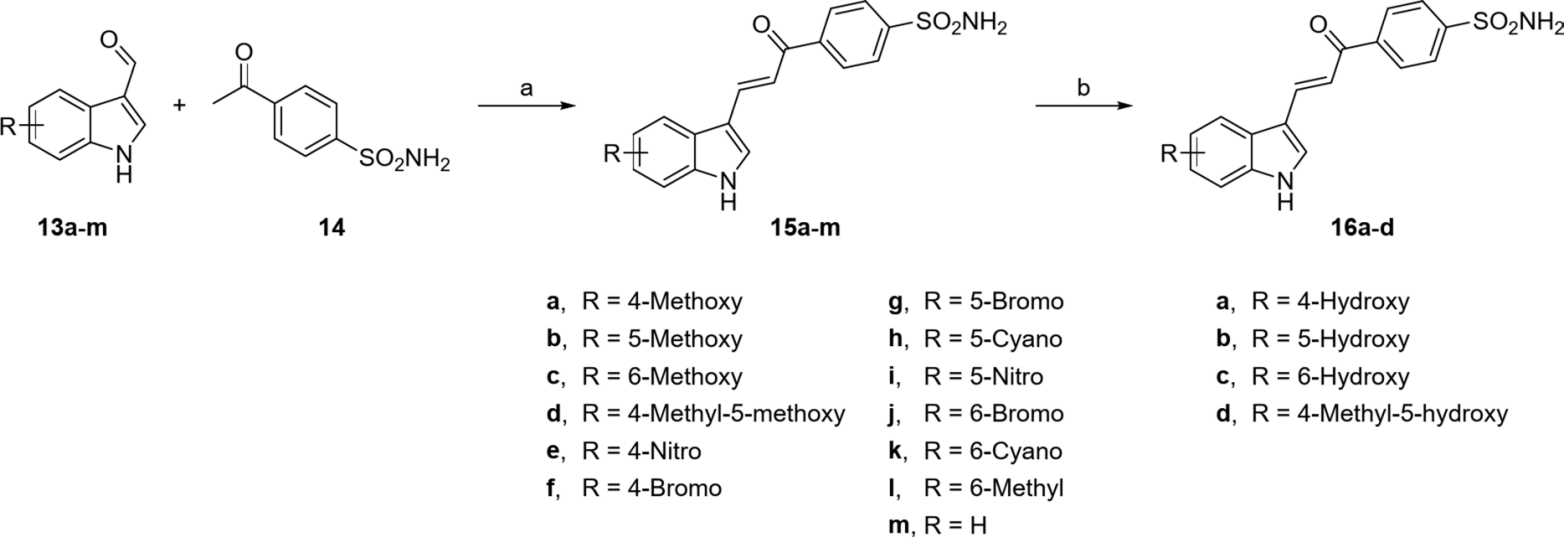
Synthesis of compounds **15a**−**m** and **16a**−**d**. Reagents and conditions: (a) piperidine, MeOH, 80 ℃, 24 h; (b) BBr_3_, DCM, 0 ℃ to room temperature, 6 h.

Scheme 1 illustrates the synthetic route employed for the preparation of target compounds **15a**−**m** and **16a**−**d**. The synthesis was achieved via a single-step aldol condensation between substituted 1*H*-indole-3-carbaldehydes **13a**−**m** and 4-acetylbenzenesulfonamide **14** in the presence of piperidine as a base, yielding the desired chalcone derivatives **15a**−**m**. Subsequently, the methoxy groups in **15a**−**d** were converted to hydroxyl groups through BBr_3_-mediated demethylation, affording **16a**−**d**. The chemical structures were confirmed using NMR spectroscopy. Notably, in the ^1^H NMR spectrum, the acetyl group peak of the starting material **14**, which appeared at approximately 2.61 ppm, disappeared after the reaction. Additionally, a new olefinic proton signal emerged in the 7.50−8.50 ppm range, corresponding to the chalcone product, with a coupling constant of 15.00–16.00 Hz, which is characteristic of an *E*-configuration double bond. Likewise, the methoxy group peak of **15a**−**d**, previously observed at approximately 4.00 ppm, disappeared following demethylation, while a new hydroxyl proton signal appeared in the 9.00−10.00 ppm range, confirming successful transformation. To the best of our knowledge, all final compounds reported in this study are novel and have not been previously described in the literature, as confirmed by database searches.

### Carbonic anhydrase inhibition study

**Fig. 3:**
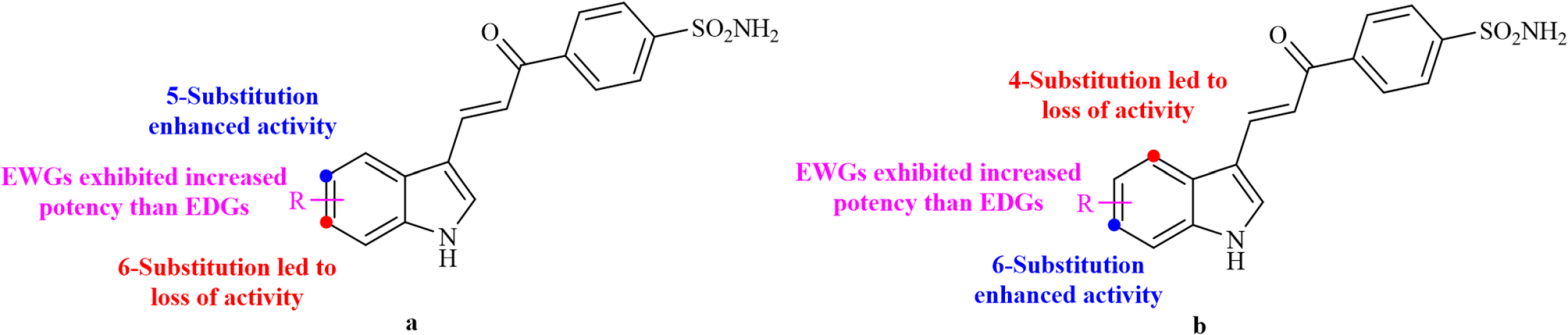
Structure-activity relationship (SAR) study of indole-chalcone derivatives against hCA IX (**a**) and hCA XII (**b**) isoforms.

As shown in Table 1, the biological evaluation of the hCA inhibitory activity of compounds **15a**−**m**, **16a**−**d** and acetazolamide (as a standard inhibitor) was performed against four isoforms of hCA using the stopped-flow CO_2_ hydrase assay. The SAR analysis (Fig. 3) of the synthesized derivatives provided valuable insight into hCA isoform inhibition. Compared to the standard inhibitor acetazolamide, which exhibited the lowest *K*_i_ values across all isoforms, most derivatives displayed weaker inhibition. However, certain derivatives demonstrated selective potency, particularly for tumor-associated isoforms hCA IX and XII.

**Table 1:** Inhibition of the hCA I, II, IX, and XII isoforms by **15a**−**m**, **16a**−**d** and standard inhibitor acetazolamide, shown as *K*_i_ values (nM).


Compound	*R*	K_i_ (nM)^a^			
		hCA I	hCA II	hCA IX	hCA XII
**15a**	4-OMe	833 ± 54	32.5 ± 1.8	50.3 ± 2.9	74.1 ± 5.6
**15b**	5-OMe	579 ± 46	23.2 ± 1.7	47.9 ± 3.6	61.3 ± 3.4
**15c**	6-OMe	853 ± 49	31.0 ± 2.2	24.6 ± 2.5	47.2 ± 2.8
**15d**	4-Me, 5-OMe	2890 ± 196	86.4 ± 5.6	20.8 ± 1.5	44.5 ± 2.6
**15e**	4-NO_2_	2554 ± 201	56.1 ± 3.2	40.1 ± 3.4	80.9 ± 5.1
**15f**	4-Br	3784 ± 370	49.9 ± 4.1	32.9 ± 2.5	31.0 ± 2.2
**15g**	5-Br	1701 ± 86	77.6 ± 6.8	14.2 ± 1.1	48.6 ± 3.1
**15h**	5-CN	551 ± 29	64.4 ± 3.5	8.9 ± 0.61	50.7 ± 3.2
**15i**	5-NO_2_	764 ± 42	46.1 ± 2.9	36.7 ± 1.9	20.0 ± 1.2
**15j**	6-Br	3079 ± 213	62.5 ± 3.8	58.4 ± 3.3	4.9 ± 0.36
**15k**	6-CN	376 ± 28	75.5 ± 6.0	87.1 ± 4.8	19.5 ± 1.0
**15l**	6-Me	1385 ± 84	93.4 ± 5.2	106 ± 8.6	33.1 ± 2.5
**15m**	H	1998 ± 101	23.4 ± 1.7	46.2 ± 4.0	41.4 ± 2.9
**16a**	4-OH	794 ± 66	38.6 ± 3.1	67.5 ± 5.0	56.2 ± 5.4
**16b**	5-OH	747 ± 51	57.3 ± 5.2	78.3 ± 4.8	27.9 ± 1.4
**16c**	6-OH	583 ± 50	76.0 ± 6.3	61.0 ± 3.9	33.8 ± 3.1
**16d**	4-Me, 5-OH	412 ± 29	51.4 ± 4.4	54.2 ± 3.3	26.1 ± 2.0
**Acetazolamide**	-	250.0 ± 13	12.0 ± 0.7	25.0 ± 1.6	5.7 ± 0.30

^a^ Mean from 3 different assays, by a stopped-flow technique (errors were in the range of ± 5−10% of the reported values).

Substituent variations on the core indole scaffold significantly influenced isoform selectivity. Bromo substitution exhibited variable effects; compound **15f** (4-Br) demonstrated poor inhibition of hCA I (*K*_i_ = 3784 nM) and moderate inhibition of hCA II (*K*_i_ = 49.9 nM). In contrast, compound **15g** (5-Br) exhibited improved inhibition of hCA I (*K*_i_ = 1701 nM) and remarkable selectivity for hCA IX (*K*_i_ = 14.2 nM). Cyano substitution showed significant effects; compound **15h** (5-CN) achieved the highest potency against hCA IX (*K*_i_ = 8.9 nM) and maintained moderate activity against hCA XII (*K*_i_ = 50.7 nM). Alternatively, compound **15k** (6-CN) demonstrated inferior inhibition of hCA IX (*K*_i_ = 87.1 nM) while exhibiting enhanced activity against hCA XII (*K*_i_ = 19.5 nM). Nitro-substituted derivatives exhibited moderate inhibition across the tested isoforms. For example, compound **15e** (4-NO_2_) displayed suboptimal inhibition of hCA I (*K*_i_ = 2554 nM) while demonstrating moderate activity against hCA II (*K*_i_ = 56.1 nM). Compound **15i** (5-NO_2_) exhibited pronounced inhibition of hCA XII (*K*_i_ = 20.0 nM). Methoxy substitution revealed position-dependent effects where compound **15d** (4-OMe) exhibited weak inhibition of hCA I (*K*_i_ = 2890 nM), while compound **15b** (5-OMe) demonstrated superior activity against hCA II (*K*_i_ = 23.2 nM) and hCA IX (*K*_i_ = 47.9 nM). Hydroxyl-bearing compounds, represented by **16a**−**d**, likely benefited from additional hydrogen bonding interactions. For instance, compound **16b** (5-OH) exhibited enhanced inhibition of hCA IX (*K*_i_ = 78.3 nM) and moderate selectivity for hCA XII (*K*_i_ = 27.9 nM). These derivatives were designed to evaluate whether introduction of a phenolic hydroxyl group could serve as an additional interaction site within the carbonic anhydrase active cavity. Consistent with this design rationale, the phenolic hydroxyl group may complement the zinc-binding sulfonamide moiety by forming secondary hydrogen bonding interactions with key amino acid residues, thereby contributing to the observed activity.

Overall, electron-withdrawing substituents such as nitro, cyano and bromo promoted selectivity toward hCA IX and XII, whereas electron-donating groups such as methoxy and hydroxyl resulted in broader inhibition profiles (Fig. 3). For hCA IX, substitution at the 5-position was well tolerated and often enhanced inhibitory activity, while substitution at the 6-position consistently resulted in a pronounced loss of activity (Fig. 3a). Among the series, compound **15h** (5-CN) emerged as the most potent hCA IX inhibitor (*K*_i_ = 8.9 nM). In contrast, the hCA XII inhibition profile displayed opposite positional preference. Substitution at the 6-position was favorable and resulted in enhanced inhibitory activity, while 4-substitution resulted in a loss of activity (Fig. 3b). Consistent with this observation, compound **15j** (6-Br) exhibited the highest potency against hCA XII (*K*_i_ = 4.9 nM), surpassing the standard inhibitor acetazolamide (*K*_i_ = 5.7 nM).

Importantly, although the absolute *K*_i_ values observed for several derivatives were in the nanomolar to submicromolar range comparable to previously reported CAIs, the newly synthesized indolylchalcone−benzenesulfonamide series demonstrated a highly desirable isoform-selective inhibitory profile, favoring hCA IX and XII over the physiologically abundant isoforms hCA I and II. Such a selectivity pattern is clinically relevant, as non-selective inhibition remains a key limitation of existing CAIs used in oncology.

**Table 2:** Selectivity indices of **15a**−**m**, **16a**−**d** and acetazolamide for the tumor-associated hCA IX and XII isoforms.

Compound	Selectivity index					
	I/IX	II/IX	XII/IX	I/XII	II/XII	IX/XII
**15a**	16.6	0.6	1.5	11.2	0.4	0.7
**15b**	12.1	0.5	1.3	9.4	0.4	0.8
**15c**	34.7	1.3	1.9	18.1	0.7	0.5
**15d**	138.9	4.2	2.1	64.9	1.9	0.5
**15e**	63.7	1.4	2.0	31.6	0.7	0.5
**15f**	115.0	1.5	0.9	122.1	1.6	1.1
**15g**	119.8	5.5	3.4	35.0	1.6	0.3
**15h**	61.9	7.2	5.7	10.9	1.3	0.2
**15i**	20.8	1.3	0.5	38.2	2.3	1.8
**15j**	52.7	1.1	0.1	628.4	12.8	11.9
**15k**	4.3	0.9	0.2	19.3	3.9	4.5
**15l**	13.1	0.9	0.3	41.8	2.8	3.2
**15m**	43.2	0.5	0.9	48.3	0.6	1.1
**16a**	11.8	0.6	0.8	14.1	0.7	1.2
**16b**	9.5	0.7	0.4	26.8	2.1	2.8
**16c**	9.6	1.2	0.6	17.2	2.2	1.8
**16d**	7.6	0.9	0.5	15.8	2.0	2.1
**Acetazolamide**	10.0	0.5	0.2	43.9	2.1	4.4

The selectivity profiles of the synthesized compounds toward the hCA isoforms were thoroughly evaluated, as summarized in Table 2. For hCA IX, selectivity indices were calculated by dividing the inhibitory potency against hCA I, II and XII by that toward hCA IX. Similarly, hCA XII selectivity was determined by dividing the inhibitory potency against hCA I, II and IX by that toward hCA XII. Overall, several compounds exhibited a clear preference for the tumor-associated isoforms. Among them, compound **15h** showed notable selectivity for hCA IX, displaying 62-, 7- and 6-fold greater selectivity over hCA I, II and XII, respectively. Likewise, compound **15j** demonstrated pronounced selectivity for hCA XII, exhibiting 628-, 13- and 12-fold greater selectivity relative to hCA I, II and IX, respectively. Notably, the substituents on the indole scaffold showed a significant effect on the observed experimental CAI selectivity of the synthesized compounds. Substitution at the 5-position of the indole ring generally resulted in improved selectivity toward hCA IX which is highlighted by compounds **15g** and **15h**. In contrast, substitution at the 6-position of the indole moiety resulted in improved selectivity toward hCA XII as observed for compound **15k**. Meanwhile, substituents at the 4-position of the indole ring exhibited comparatively lower selectivity profiles. 

To further assess these observations, we compared the present scaffold with previously reported hCA inhibitors. Chalcone-based benzenesulfonamides have been reported to exhibit effective carbonic anhydrase inhibition through incorporation of a sulfonamide zinc-binding group. However, these compounds displayed inhibitory activity across multiple hCA isoforms, reflecting the inherent challenge of achieving isoform selectivity among closely related carbonic anhydrases^36^. In comparison, the previously reported indole-based benzenesulfonamides demonstrated promising inhibitory potency, although their isoform selectivity profiles were not systematically optimized^40^. The indolylchalcone-benzenesulfonamide scaffold employed in this study integrates structural features of both chalcone- and indole-based benzenesulfonamides while providing improved flexibility in modulating isoform selectivity. In particular, the pronounced selectivity observed for compounds **15h** and **15j** highlights the potential of this hybrid framework to preferentially target tumor-associated isoforms hCA IX and XII. Despite these advantages, the present data also indicate that isoform selectivity is highly sensitive to substituent position on the indole ring, which may complicate rational optimization. Overall, these results demonstrate that the indolylchalcone-benzenesulfonamide scaffold represents a promising yet structurally sensitive platform for the development of selective inhibitors targeting tumor-associated carbonic anhydrases.

### Molecular docking study

**Fig. 4:**
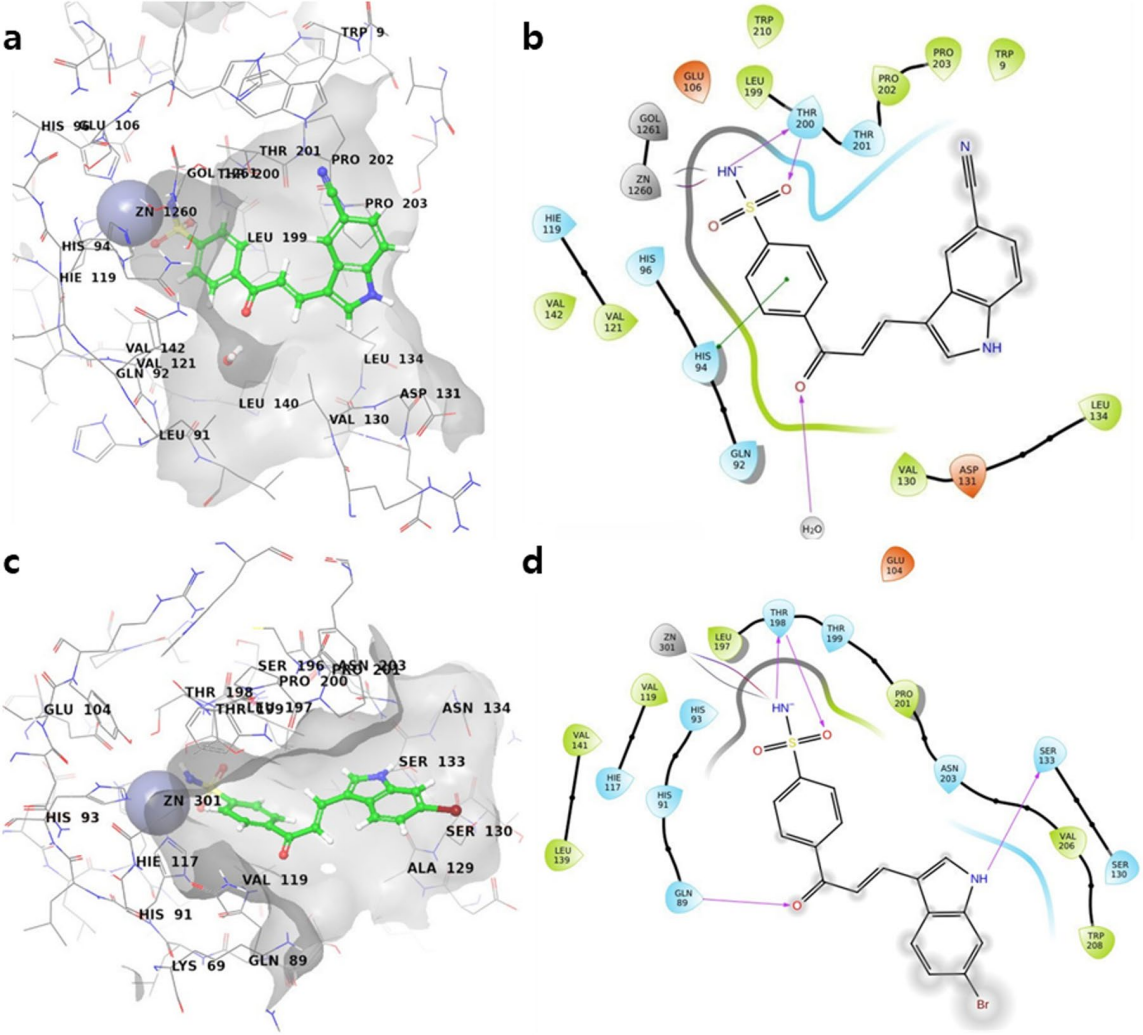
Docked complexes of compounds **15h** and **15j** with hCA IX and XII isoforms, respectively. (**a**) 3D model of hCA IX complexed with **15h** (PDB ID: 5FL5, resolution: 2.05 Å); (**b**) 2D interaction map showing the binding interactions of **15h** within the active site of hCA IX; (**c**) 3D model of hCA XII complexed with **15j** (PDB ID: 4HT2, resolution: 1.45 Å); (**d**) 2D interaction map of showing the binding interactions of **15j** within the active site of hCA XII. Favorable interactions are color-coded as follows: pink for hydrogen bonds and black for coordinate (dative) interactions.

Molecular docking study is a computational tool which is employed to predict the binding interaction between a ligand and its biological target^41^. Molecular docking plays a key role in understanding the structural basis of ligand-protein interactions which provides insights into understanding the observed experimental binding affinity^42^. Herein we employed molecular docking in order to evaluate the ligand/protein binding affinity. Moreover, we attempted to identify the key amino acid residues which are predicted to contribute to the stability of the synthesized ligand to guide future rational drug design and optimization efforts^41,43^. Accordingly, molecular docking studies were conducted to elucidate the binding modes of compounds **15h** and **15j** with human carbonic anhydrase isoforms hCA IX and XII, respectively (Fig. 4). Both compounds displayed a canonical binding orientation characteristic of sulfonamide-based carbonic anhydrase inhibitors. Specifically, the deprotonated sulfonamide moiety (-SO_2_NH^−^) of each compound formed a strong coordinate (dative) bond with the catalytic Zn^2+^ ion which contributed to the stability of the established ligand-protein complexes. Compound **15h** occupied the same binding site as the reference compound acetazolamide (Figure S1), demonstrating a shared binding modality. Both compounds formed a hydrogen bond with Thr200, adjacent to the catalytic zinc ion, suggesting a conserved mode of interaction within the active site.

Notably, compound **15j** formed an additional hydrogen bond through its indole ring, where the nitrogen atom of the indole established a hydrogen bond with Ser133 of hCA XII. Interestingly, it was observed that both the bromo (Br) and cyano (CN) substituents were not predicted to establish a direct interaction with the binding site residues which indicates that their involvement may be due to steric effects where the Br or CN substituents may function as conformational anchors restricting rotational flexibility. We would like to highlight that the current docking study is limited in its ability to distinguish between the *E* and *Z* isomers. 

### Molecular dynamics (MD) analysis

**Fig. 5:**
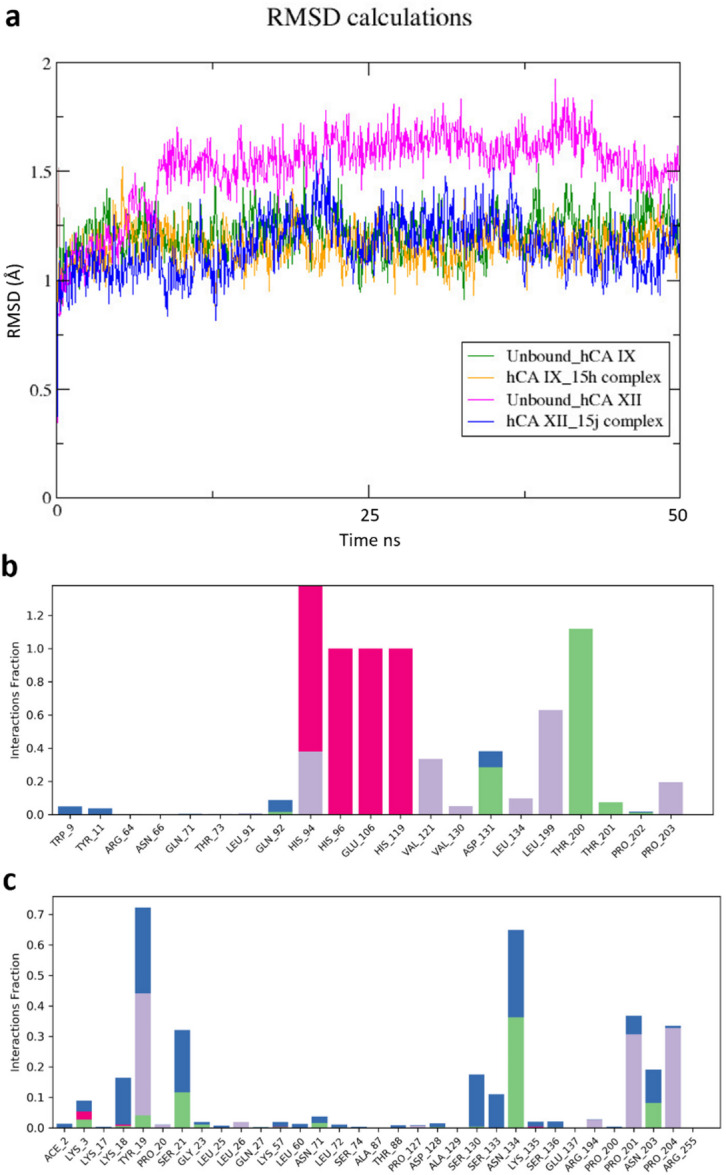
Molecular dynamics (MD) analysis of compounds **15h** and **15j** with hCA IX and XII, respectively. (**a**) RMSD plots comparing the unbound hCA IX (green), hCA IX/**15h** complex (orange), unbound hCA XII (magenta) and hCA XII/**15j** complex (blue). (**b,c**) Protein-ligand interaction histograms for the hCA IX/**15h** and hCA XII/**15j** complexes, respectively. Interaction types are color-coded: green for hydrogen bonds, purple for hydrophobic interactions, red for coordinate (dative) interactions and blue for water bridges.

Four fifty nanosecond molecular dynamic simulations were carried out to predict the stability of the ligands when binding to carbonic anhydrase. The four molecular dynamic simulations included two runs for the unbound hCA IX and XII proteins which were used as reference points for their respective ligand-bound complexes. The MD simulation of compound **15h** showed that the hCA IX/**15h** complex exhibited of a root mean square deviation (RMSD) of 1.15 Å. Meanwhile, the unbound hCA IX protein exhibited an average RMSD of 1.2 Å., as shown in Fig. 5a. This reduction in RMSD indicates that the binding of **15h** contributes to protein stabilization which is consistent with its observed experimental inhibitory activity. Further analysis of the protein-ligand interaction histogram (Fig. 5b) revealed that the hydrogen bond between the sulfonamide group of **15h** and Thr200 remained stable and uninterrupted throughout the simulation which suggests that this bond is important in maintaining complex stability. Additionally, multiple interactions were observed between **15h** and His94, His96, Glu106 and His119 residues of hCA IX. These interactions are predicted to contribute to the observed stability of **15h**/hCA IX and validating the predicted binding mode.

The unbound hCA XII exhibited greater RMSD fluctuations compared to hCA IX, with an average RMSD of 1.5 Å. However, binding with compound **15j** resulted in a pronounced stabilizing effect, as evidenced by the reduced average RMSD of 1.15 Å for the hCA XII/**15j** complex, indicating minimized structural deviations and supporting the compound’s inhibitory activity. Throughout its MD simulation, compound **15j** established multiple stable interactions within the active site of hCA XII where interactions involving Tyr19 and Asn134 persisted for over 60% of the simulation time (Fig. 5c). The observed sustained nature of these interactions predict their role in stabilizing the binding of **15j** within the binding site of hCA XII complex and further validate the predicted binding mode of **15j**.

We would like to highlight that the current docking study is a prediction of the binding modes of the synthesized CA inhibitors. Future work should involve the co-crystallization of optimized leads. Herein, our molecular docking predictions were further validated through MD simulations, which maintained stable Zn^2+^ coordination and conserved hydrogen-bond interactions throughout the trajectory. The combined docking − MD approach therefore provides a mechanistic rationale for the observed inhibition and selectivity trends, despite the absence of experimentally solved protein−ligand complexes.

### ADME calculations

The freely accessible Swiss-ADME server was employed to predict the physicochemical properties of compounds **15j** and **15h**, compared to acetazolamide (Figure S2)^44^. Compound **15j** is predicted to lie closer to the region associated with passive absorption into systemic circulation, indicating a more favorable balance that may support membrane permeability and oral bioavailability. Meanwhile, compound **15h** is predicted to exhibit increased polarity with similar lipophilicity, which may reduce permeability and limit absorption despite potentially improved solubility.

## Materials and methods

### Chemistry

#### General procedures

All commercially available reagents and solvents were of reagent grade and used without further purification unless otherwise stated. Reactions were monitored by thin-layer chromatography (TLC) using 0.25 mm silica gel 60 F254 plates (E. Merck) and visualized under UV light. Proton nuclear magnetic resonance (^1^H NMR) spectra were recorded on a Varian 400 MHz spectrometer (Varian Medical Systems, Inc., Palo Alto, CA, USA), while carbon-13 nuclear magnetic resonance (^13^C NMR) spectra were obtained on a Varian 100 MHz spectrometer. Chemical shifts (*δ*) are reported in parts per million (ppm) relative to tetramethylsilane (TMS) as an internal standard. Coupling constants (*J*) are reported in hertz (Hz). Multiplicities are denoted as follows: singlet (s), doublet (d), doublet of doublets (dd), triplet (t), quartet (q), multiplet (m) and broad (br). High-resolution electrospray ionization mass spectrometry (HR-ESI-MS) data were collected using a G2 QTOF mass spectrometer (Waters Corporation, Milford, MA, USA). Reverse-phase high performance liquid chromatography (RP-HPLC) was performed to determine compound purity. A UV detector was set at 254 nm. Mobile phase consisted of (A) H_2_O containing 0.05% trifluoroacetic acid (TFA) and (B) acetonitrile (CH_3_CN). The final product purity was assessed using a gradient elution of 75% B over 30 min. The purity of all the biologically evaluated compounds was > 95.0%. Melting points of the compounds were measured using a Thermo Scientific 9200 apparatus.

#### Synthesis of 15a−m

To a stirring solution of substituted indole-3-carbaldehyde **13a**−**m** (1.0 eq) in MeOH (20 mL) was added piperidine (1.0 eq) and 4-acetylbenzenesulfonamide (**14, **1.1 eq). The resulting mixture was stirred at 80 ℃ for 24 h. The reaction mixture was then evaporated under reduced pressure and a small amount of cold MeOH was added for recrystallization. The crude was filtered and washed with hexane, diethyl ether and ethanol.

##### (*E*)-4-(3-(4-Methoxy-1*H*-indol-3-yl)acryloyl)benzenesulfonamide (15a)

Obtained as orange solid (0.51 g, 62.7%). ^1^H NMR (400 MHz, DMSO-*d*_6_) δ 11.96 (s, 1H), 8.39 (d, *J* = 15.5 Hz, 1H), 8.26 (s, 1H), 8.19 (d, *J* = 8.4 Hz, 2 H), 7.98 (d, *J* = 8.4 Hz, 2 H), 7.75 (d, *J* = 15.5 Hz, 1H), 7.52 (s, 2 H), 7.14–7.05 (m, 2 H), 6.68 (d, *J* = 7.6 Hz, 1H), 3.94 (s, 3 H); ^13^C NMR (100 MHz, DMSO-*d*_6_) δ 193.09, 159.35, 152.07, 146.48, 143.63, 133.85, 133.69, 131.23, 131.08, 128.69, 121.34, 120.89, 118.25, 110.93, 106.97, 60.56; HRMS (EI) *m/z* calcd for C_18_H_17_N_2_O_4_S [M + H]^+^ 357.0909, found 357.0898; RP-HPLC purity 98.0% at 254 nm, *t*_R_ = 12.70 min; melting point: 250−251 ℃.

##### (*E*)-4-(3-(5-Methoxy-1*H*-indol-3-yl)acryloyl)benzenesulfonamide (15b)

Obtained as purple solid (0.45 g, 44.2%). ^1^H NMR (400 MHz, DMSO-*d*_6_) δ 11.88 (s, 1H), 8.25 (d, *J* = 8.3 Hz, 2 H), 8.13 (s, 1H), 8.09 (d, *J* = 15.5 Hz, 1H), 7.98 (d, *J* = 8.3 Hz, 2 H), 7.60–7.51 (m, 3 H), 7.48 (d, *J* = 2.4 Hz, 1H), 7.40 (d, *J* = 8.7 Hz, 1H), 6.89 (dd, *J* = 8.7, 2.4 Hz, 1H), 3.87 (s, 3 H); ^13^C NMR (100 MHz, DMSO-*d*_6_) δ 188.63, 155.56, 147.31, 141.67, 140.56, 134.11, 132.83, 129.33, 129.18, 126.48, 126.45, 115.27, 113.70, 113.15, 112.78, 103.12, 56.05; HRMS (EI) *m/z* calcd for C_18_H_17_N_2_O_4_S [M + H]^+^ 357.0909, found 357.0903; RP-HPLC purity 95.4% at 254 nm, *t*_R_ = 12.07 min; melting point: 269−270 ℃.

##### (*E*)-4-(3-(6-Methoxy-1*H*-indol-3-yl)acryloyl)benzenesulfonamide (15c)

Obtained as yellow solid (0.46 g, 56.5%). ^1^H NMR (400 MHz, DMSO-*d*_6_) δ 11.76 (s, 1H), 8.24 (d, *J* = 8.5 Hz, 2 H), 8.04–7.95 (m, 5 H), 7.55 (d, *J* = 15.9 Hz, 3 H), 6.98 (d, *J* = 2.3 Hz, 1H), 6.85 (dd, *J* = 8.7, 2.3 Hz, 1H), 3.79 (s, 3 H); ^13^C NMR (100 MHz, DMSO-*d*_6_) δ 188.57, 156.92, 147.34, 141.53, 140.75, 139.11, 133.98, 129.21, 126.43, 121.81, 119.44, 115.24, 113.53, 111.53, 96.07, 55.73; HRMS (EI) *m/z* calcd for C_18_H_17_N_2_O_4_S [M + H]^+^ 357.0909, found 357.0896; RP-HPLC purity 97.2% at 254 nm, *t*_R_ = 11.95 min; melting point: 265−266 ℃.

##### (*E*)-4-(3-(5-Methoxy-4-methyl-1*H*-indol-3-yl)acryloyl)benzenesulfonamide (15d)

Obtained as red solid (0.15g, 38.3%). ^1^H NMR (400 MHz, DMSO-*d*_6_) δ 11.89 (s, 1H), 8.42–8.38 (m, 2 H), 8.23 (d, *J* = 8.5 Hz, 2 H), 7.96 (d, *J* = 8.4 Hz, 2 H), 7.66 (d, *J* = 15.1 Hz, 1H), 7.51 (s, 2 H), 7.26 (d, *J* = 8.7 Hz, 1H), 6.94 (d, *J* = 8.8 Hz, 1H), 3.78 (s, 3 H), 2.53 (s, 3 H); ^13^C NMR (100 MHz, DMSO-*d*_6_) δ 187.85, 152.58, 147.35, 141.61, 132.82, 130.28, 129.10, 126.62, 126.36, 117.09, 115.58, 114.11, 110.91, 109.77, 57.58, 12.86; HRMS (EI) *m/z* calcd for C_19_H_19_N_2_O_4_S [M + H]^+^ 371.1066, found 371.1055; RP-HPLC purity 97.6% at 254 nm, *t*_R_ = 13.33 min; melting point: 287−288 ℃.

##### (*E*)-4-(3-(4-Nitro-1*H*-indol-3-yl)acryloyl)benzenesulfonamide (15e)

Obtained as yellow solid (0.20 g, 60.2%). ^1^H NMR (400 MHz, DMSO-*d*_6_) δ 12.70 (s, 1H), 8.71 (s, 1H), 8.29–8.22 (m, 3 H), 7.98 (d, *J* = 8.5 Hz, 2 H), 7.92 (ddd, *J* = 10.4, 8.0, 1.0 Hz, 2 H), 7.64 (d, *J* = 15.4 Hz, 1H), 7.54 (s, 2 H), 7.37 (t, *J* = 8.0 Hz, 1H); ^13^C NMR (100 MHz, DMSO-*d*_6_) δ 193.31, 152.38, 147.37, 145.90, 144.68, 138.15, 134.00, 131.24, 131.08, 126.78, 124.58, 124.13, 123.05, 116.45; HRMS (EI) *m/z* calcd for C_17_H_14_N_3_O_5_S [M + H]^+^ 372.0654, found 372.0636; RP-HPLC purity 95.8% at 254 nm, *t*_R_ = 12.63 min; melting point: 301−302 ℃.

##### (*E*)-4-(3-(4-Bromo-1*H*-indol-3-yl)acryloyl)benzenesulfonamide (15f)

Obtained as yellow solid (0.07 g, 19.4%). ^1^H NMR (400 MHz, DMSO-*d*_6_) δ 12.33 (s, 1H), 8.85 (d, *J* = 15.4 Hz, 1H), 8.57 (s, 1H), 8.23 (d, *J* = 8.6 Hz, 2 H), 7.97 (d, *J* = 8.5 Hz, 2 H), 7.75 (d, *J* = 15.4 Hz, 1H), 7.53–7.49 (m, 3 H), 7.36 (dd, *J* = 7.7, 0.8 Hz, 1H), 7.10 (t, *J* = 7.8 Hz, 1H); ^13^C NMR (100 MHz, DMSO-*d*_6_) δ 188.30, 147.52, 141.32, 139.25, 138.69, 130.38, 129.22, 126.45, 126.40, 125.88, 124.58, 123.86, 116.92, 113.29, 112.87; HRMS (EI) *m/z* calcd for C_17_H_14_N_2_O_3_SBr [M + H]^+^ 404.9909, found 404.9890; RP-HPLC purity 96.6% at 254 nm, *t*_R_ = 14.53 min; melting point: 282−283 ℃.

##### (*E*)-4-(3-(5-Bromo-1*H*-indol-3-yl)acryloyl)benzenesulfonamide (15g)

Obtained as yellow solid (6.0 g, 66.3%). ^1^H NMR (400 MHz, DMSO-*d*_6_) δ 12.15 (s, 1H), 8.31–8.22 (m, 4 H), 8.07 (d, *J* = 15.5 Hz, 1H), 7.99 (d, *J* = 8.4 Hz, 2 H), 7.63 (d, *J* = 15.5 Hz, 1H), 7.57 (s, 2 H), 7.47 (d, *J* = 8.6 Hz, 1H), 7.38 (dd, *J* = 8.6, 1.8 Hz, 1H); ^13^C NMR (100 MHz, DMSO-*d*_6_) δ 188.84, 147.47, 141.37, 139.68, 136.68, 134.96, 129.36, 127.38, 126.40, 125.92, 122.99, 116.56, 114.92, 114.52, 112.90. HRMS (EI) *m/z* calcd for C_17_H_14_N_2_O_3_SBr [M + H]^+^ 404.9909, found 404.9905; RP-HPLC purity 97.1% at 254 nm, *t*_R_ = 14.59 min; melting point: 295−296 ℃.

##### (*E*)-4-(3-(5-Cyano-1*H*-indol-3-yl)acryloyl)benzenesulfonamide (15h)

Obtained as yellow solid (0.18 g, 37.9%). ^1^H NMR (400 MHz, DMSO-*d*_6_) δ 12.42 (s, 1H), 8.76 (s, 1H), 8.37–8.33 (m, 3 H), 8.10 (d, *J* = 15.6 Hz, 1H), 8.01 (d, *J* = 8.4 Hz, 2 H), 7.78 (d, *J* = 15.6 Hz, 1H), 7.68 (d, *J* = 8.4 Hz, 1H), 7.62 (dd, *J* = 8.4, 1.4 Hz, 1H), 7.59 (s, 2 H); ^13^C NMR (100 MHz, DMSO-*d*_6_) δ 188.84, 147.61, 141.10, 139.75, 139.05, 136.02, 129.52, 129.13, 126.39, 126.24, 125.26, 120.89, 117.50, 114.19, 113.88, 103.82; HRMS (EI) *m/z* calcd for C_18_H_14_N_3_O_3_S [M + H]^+^ 352.0756, found 352.0737; RP-HPLC purity 99.0% at 254 nm, *t*_R_ = 11.73 min; melting point: 296−297 ℃.

##### (*E*)-4-(3-(5-Nitro-1*H*-indol-3-yl)acryloyl)benzenesulfonamide (15i)

Obtained as yellow solid (0.14 g, 35.9%). ^1^H NMR (400 MHz, DMSO-*d*_6_) δ 12.51 (s, 1H), 8.92 (d, *J* = 2.2 Hz, 1H), 8.43 (s, 1H), 8.25 (d, *J* = 8.5 Hz, 2 H), 8.13–8.10 (m, 1H), 8.10–8.08 (m, 1H), 7.99 (d, *J* = 8.4 Hz, 2 H), 7.73 (d, *J* = 15.6 Hz, 1H), 7.67 (d, *J* = 9.0 Hz, 1H), 7.54 (s, 2 H); ^13^C NMR (100 MHz, DMSO-*d*_6_) δ 189.00, 147.64, 142.63, 141.19, 140.88, 138.38, 135.68, 129.37, 126.47, 125.26, 118.44, 117.24, 115.01, 113.54; HRMS (EI) *m/z* calcd for C_17_H_14_N_3_O_5_S [M + H]^+^ 372.0654, found 372.0637; RP-HPLC purity 95.0% at 254 nm, *t*_R_ = 12.80 min; melting point: 283−284 ℃.

##### (*E*)-4-(3-(6-Bromo-1*H*-indol-3-yl)acryloyl)benzenesulfonamide (15j)

Obtained as yellow solid (3.5 g, 38.7%). ^1^H NMR (400 MHz, DMSO-*d*_6_) δ 11.88 (s, 1H), 8.28 (d, *J* = 6.2 Hz, 2 H), 8.18 (s, 1H), 8.11–8.04 (m, 2 H), 7.98 (dd, *J* = 8.5, 2.4 Hz, 2 H), 7.64 (q, *J* = 22.4, 19.6 Hz, 4 H), 7.35 (d, *J* = 8.5 Hz, 1H); ^13^C NMR (100 MHz, DMSO-*d*_6_) δ 188.65, 147.51, 141.29, 139.83, 138.89, 134.95, 129.31, 126.43, 124.58, 124.45, 122.73, 116.46, 115.89, 115.59, 113.34; HRMS (EI) *m/z* calcd for C_17_H_14_N_2_O_3_SBr [M + H]^+^ 404.9909, found 404.9888; RP-HPLC purity 98.8% at 254 nm, *t*_R_ = 18.24 min; melting point: 271−272 ℃.

##### (*E*)-4-(3-(6-Cyano-1*H*-indol-3-yl)acryloyl)benzenesulfonamide (15k)

Obtained as yellow solid (0.080 g, 25.8%). ^1^H NMR (400 MHz, DMSO-*d*_6_) δ 12.42 (s, 1H), 8.76 (s, 1H), 8.37–8.32 (m, 3 H), 8.10 (d, *J* = 15.6 Hz, 1H), 8.01 (d, *J* = 8.1 Hz, 2 H), 7.77 (d, *J* = 15.6 Hz, 1H), 7.67 (d, *J* = 8.4 Hz, 1H), 7.62 (dd, *J* = 8.4, 1.4 Hz, 1H), 7.58 (s, 2 H); ^13^C NMR (100 MHz, DMSO-*d*_6_) δ 188.84, 147.61, 141.10, 139.75, 139.04, 136.02, 129.52, 126.44, 126.39, 126.24, 125.26, 120.89, 117.50, 114.18, 113.88, 103.82; HRMS (EI) *m/z* calcd for C_18_H_14_N_3_O_3_S [M + H]^+^ 352.0756, found 352.0744; RP-HPLC purity 97.1% at 254 nm, *t*_R_ = 11.89 min; melting point: 297−298 ℃.

##### (*E*)-4-(3-(6-Methyl-1*H*-indol-3-yl)acryloyl)benzenesulfonamide (15l)

Obtained as yellow solid (0.19 g, 44.4%). ^1^H NMR (400 MHz, DMSO-*d*_6_) δ 11.82 (s, 1H), 8.24 (d, *J* = 8.4 Hz, 2 H), 8.06–8.02 (m, 2 H), 7.97 (dd, *J* = 8.5, 2.0 Hz, 3 H), 7.56 (d, *J* = 15.4 Hz, 1H), 7.52 (s, 2 H), 7.27 (s, 1H), 7.06 (dd, *J* = 8.4, 1.5 Hz, 1H), 2.42 (s, 3 H); ^13^C NMR (100 MHz, DMSO-*d*_6_) δ 188.51, 147.36, 141.54, 140.81, 138.56, 134.36, 132.69, 129.21, 126.43, 123.44, 123.35, 120.81, 115.29, 113.35, 112.82, 21.77; HRMS (EI) *m/z* calcd for C_18_H_17_N_2_O_3_S [M + H]^+^ 341.0960, found 341.0945; RP-HPLC purity 97.8% at 254 nm, *t*_R_ = 13.46 min; melting point: 287−288 ℃.

##### (*E*)-4-(3-(1*H*-Indol-3-yl)acryloyl)benzenesulfonamide (15m)

Obtained as yellow solid (0.25 g, 61.0%). ^1^H NMR (400 MHz, DMSO-*d*_6_) δ 11.96 (s, 1H), 8.25 (d, *J* = 8.5 Hz, 2 H), 8.13 (d, *J* = 2.8 Hz, 1H), 8.11–8.05 (m, 2 H), 7.97 (d, *J* = 8.4 Hz, 2 H), 7.61 (d, *J* = 15.5 Hz, 1H), 7.53 (s, 2 H), 7.50–7.47 (m, 1H), 7.26–7.21 (m, 2 H); ^13^C NMR (100 MHz, DMSO-*d*_6_) δ 188.54, 147.41, 141.47, 140.64, 138.06, 134.57, 129.24, 126.44, 125.53, 123.36, 121.81, 121.04, 115.54, 113.34, 113.01; HRMS (EI) *m/z* calcd for C_17_H_15_N_2_O_3_S [M + H]^+^ 327.0803, found 327.0788; RP-HPLC purity 98.0% at 254 nm, *t*_R_ = 12.04 min; melting point: 283−284 ℃.

#### Synthesis of 16a−d

To a stirring solution of **15a**−**d** (1.0 eq) in DCM (20 mL) was added BBr_3_ (5.0 eq) at 0 ℃ under argon atmosphere. The resulting mixture was stirred at room temperature for 6 h. The reaction mixture was then quenched by adding ice cold water. The resulting mixture was diluted with DCM (30 mL), and the aqueous phase was extracted with DCM (3 × 10 mL). The combined organic layer was dried over anhydrous magnesium sulfate, filtered, and evaporated under reduced pressure. The crude was filtered and washed with hexane, diethyl ether and ethanol.

##### (*E*)-4-(3-(4-Hydroxy-1*H*-indol-3-yl)acryloyl)benzenesulfonamide (16a)

Obtained as black solid (0.080 g, 55.5%). ^1^H NMR (400 MHz, DMSO-*d*_6_) δ 11.82 (s, 1H), 9.90 (s, 1H), 8.41 (d, *J* = 15.4 Hz, 1H), 8.22–8.15 (m, 3 H), 7.96 (d, *J* = 8.6 Hz, 2 H), 7.84 (d, *J* = 15.5 Hz, 1H), 7.52 (s, 2 H), 6.96 (t, *J* = 7.8 Hz, 1H), 6.90 (d, *J* = 7.4 Hz, 1H), 6.54 (d, *J* = 7.1 Hz, 1H); ^13^C NMR (100 MHz, DMSO-*d*_6_) δ 188.30, 170.83, 152.39, 147.27, 141.94, 141.88, 139.53, 129.08, 128.97, 126.38, 123.95, 116.44, 115.72, 113.82, 106.35, 104.27; HRMS (EI) *m/z* calcd for C_17_H_15_N_2_O_4_S [M + H]^+^ 343.0753, found 343.0747; RP-HPLC purity 97.8% at 254 nm, *t*_R_ = 8.15 min; melting point > 400 ℃.

##### (*E*)-4-(3-(5-Hydroxy-1*H*-indol-3-yl)acryloyl)benzenesulfonamide (16b)

Obtained as black solid (1.8 g, 93.7%). ^1^H NMR (400 MHz, DMSO-*d*_6_) δ 11.75 (s, 1H), 9.04 (s, 1H), 8.18 (d, *J* = 8.4 Hz, 2 H), 8.03–7.95 (m, 4 H), 7.52 (s, 2 H), 7.42 (d, *J* = 15.4 Hz, 1H), 7.33–7.27 (m, 2 H), 6.74 (dd, *J* = 8.7, 2.2 Hz, 1H); ^13^C NMR (100 MHz, DMSO-*d*_6_) δ 188.57, 153.24, 147.26, 141.85, 141.11, 134.75, 132.20, 129.00, 126.56, 126.45, 114.64, 113.57, 113.13, 112.75, 105.26; HRMS (EI) *m/z* calcd for C_17_H_15_N_2_O_4_S [M + H]^+^ 343.0753, found 343.0745; RP-HPLC purity 98.3% at 254 nm, *t*_R_ = 8.59 min; melting point: 284−285 ℃.

##### (*E*)-4-(3-(6-Hydroxy-1*H*-indol-3-yl)acryloyl)benzenesulfonamide (16c)

Obtained as black solid (0.11 g, 35.8%). ^1^H NMR (400 MHz, DMSO-*d*_6_) δ 11.60 (s, 1H), 8.24 (d, *J* = 8.5 Hz, 2 H), 8.01–7.95 (m, 3 H), 7.92–7.87 (m, 2 H), 7.55–7.48 (m, 3 H), 6.83 (d, *J* = 2.1 Hz, 1H), 6.74 (dd, *J* = 8.5, 2.2 Hz, 1H); ^13^C NMR (100 MHz, DMSO-*d*_6_) δ 188.46, 154.72, 147.30, 141.61, 140.96, 139.51, 133.91, 129.19, 126.42, 121.78, 118.47, 114.78, 113.66, 111.94, 98.17; HRMS (EI) *m/z* calcd for C_17_H_15_N_2_O_4_S [M + H]^+^ 343.0753, found 343.0749; RP-HPLC purity 97.08% at 254 nm, *t*_R_ = 7.14 min; melting point: 228−229 ℃.

##### (*E*)-4-(3-(5-Methoxy-4-methyl-1*H*-indol-3-yl)acryloyl)benzenesulfonamide (16d)

Obtained as orange solid (0.030 g, 67.8%). ^1^H NMR (400 MHz, DMSO-*d*_6_) δ 11.78 (s, 1H), 8.79 (s, 1H), 8.39 (d, *J* = 15.1 Hz, 1H), 8.33 (d, *J* = 3.1 Hz, 1H), 8.22 (d, *J* = 8.4 Hz, 2 H), 7.95 (d, *J* = 8.2 Hz, 2 H), 7.63 (d, *J* = 15.1 Hz, 1H), 7.50 (s, 2 H), 7.09 (d, *J* = 8.6 Hz, 1H), 6.75 (d, *J* = 8.6 Hz, 1H), 2.49 (s, 3 H); ^13^C NMR (100 MHz, DMSO-*d*_6_) δ 187.76, 150.15, 147.28, 141.72, 141.69, 131.70, 129.45, 129.05, 126.86, 126.34, 115.07, 114.10, 114.02, 112.84, 110.74, 12.96; HRMS (EI) *m/z* calcd for C_18_H_17_N_2_O_4_S [M + H]^+^ 357.0909, found 357.0895; RP-HPLC purity 97.8% at 254 nm, *t*_R_ = 8.05 min; melting point: 312−313 ℃.

### Carbonic anhydrase inhibition study

The catalytic amount of carbonic anhydrase (CA)-mediated CO_2_ hydration was evaluated using a stopped-flow instrument (Applied Photophysics, UK). The pH indicator phenol red was used at a concentration of 0.2 nM, with the absorbance monitored at 557 nm. The assay was performed in 20 mM HEPES buffer (pH 7.4) containing 10 mM NaClO_4_ to maintain a constant ionic strength. The reaction was initiated after a delay period of 10–100 s, during which the enzyme and inhibitor were preincubated together at room temperature for 15 min to allow for the formation of enzyme-inhibitor (E-I) complex. Carbon dioxide (CO_2_) was used as the substrate at varying concentrations ranging from 1.7 to 17 mM to determine kinetic parameters and inhibition constants. The initial reaction velocities were calculated from at least six kinetic traces corresponding to the first 5−10% of the reaction. Uncatalyzed reaction rates were also measured separately and subtracted from the total measured rates to obtain CA-specific activity. Stock inhibitor solutions (10 mM) were prepared in DMSO and serially diluted down to 0.01 nM using 20 mM HEPES buffer (pH 7.4). The inhibition constants (*K*_i_) were calculated using GraphPad Prism 3 software. All measurements represent the mean of at least three independent experiments, with associated errors typically ranging from ± 5−10% of the reported values^39,40^.

### Molecular docking study

The crystal structure of human carbonic anhydrase isoforms hCA IX (PDB ID: 5FL5, resolution 2.05 Å)^45^ and hCA XII (PDB ID: 4HT2, resolution 1.45 Å)^46^ were retrieved from the Protein Data Bank (www.pdb.org, accessed on 21 March 2025). Protein structures were prepared using the Protein Preparation Wizard in the Schrodinger 2021 suite^47^ under default settings, with the target pH set to 7.4. Hydrogen atoms were added, missing side chains and loops were filled, bond orders were assigned and water molecules beyond 5 Å from the active site were removed. The protein structures were then minimized using the OPLS3e force field. Ligands were initially drawn using ChemDraw^48^ Professional 17.0 and exported as SDF (Structure Data File) format. Ligands were prepared in Maestro using LigPrep with Epik to generate ionization and tautomeric states at pH 7.0 ± 2.0 (default). Epik state penalties were retained and applied to the docking score during Glide docking (default Maestro/Glide settings). Molecular docking was carried out using the Glide module in the standard precision (SP) mode. Each ligand was rigidly docked into the active site of the prepared protein, where the co-crystallized ligand pose was used as the direct reference for binding mode comparison. The pose with the most favorable (most negative) docking score was selected for further analysis and visualization^36^.

### Molecular dynamics study

Molecular dynamics (MD) simulations were carried out using DESMOND software (version 2021.2). An orthorhombic simulation box was generated and solvated with explicit single-point charge (SPC) water molecules. The system temperature was maintained at 300 K and the pressure at 1 bar throughout the simulation. The total simulation time was set to 50 ns, with a 1 ps relaxation time applied to selected positions. Energy minimization of the solvated system was performed using the OPLS 2005 force field. Electrostatic interactions were treated using the particle mesh Ewald (PME) method. Periodic boundary conditions (PBC) were applied, and a 9.0 Å cutoff was used for nonbonded interactions. A six-step relaxation protocol was followed prior to the production run. This included 2000 steps of LBFGS minimization with solute restraints and a loose convergence criterion of 50 kcal mol^− 1^ Å^−1^. Two equilibration simulations of 12 ns each were conducted: one for temperature equilibration at 10 K (thermostat relaxation constant = 0.1 ps) and one for pressure equilibration at 1 atm (barostat relaxation constant = 50 ps), both with nonhydrogen solute atoms restrained. A final 24 ps NPT ensemble simulation was run at 300 K and 1 atm (thermostat relaxation constant = 0.1 ps) to complete the preparation phase. Following relaxation, a 50 ns MD simulation was performed in the NPT ensemble using a Nose–Hoover thermostat (relaxation time = 1.0 ps) and a Martyna-Tobias-Klein barostat (relaxation time = 2.0 ps). No Zn^2+^ coordination or backbone H-bond constraints were enforced during the simulation. Trajectory analysis and data plotting^49^ were conducted using QtGrace (version 2009) and Microsoft Excel^49,50^.

## Conclusion

In this study, we designed and synthesized a novel hybrid series of indolylchalcone–benzenesulfonamide hybrids (**15a**−**m** and **16a**−**d**) *via* combining the indole and chalcone pharmacophores with a sulfonamide ZBG, as potential selective human carbonic anhydrase (hCA) inhibitors. Among them, compound **15h** (5-CN) showed potent and selective inhibition of hCA IX (*K*_i_ = 8.9 nM) with selectivity over hCA I, II, and XII (SI = 61.9, 7.2, and 5.7, respectively), while compound **15j** (6-Br) exhibited strong activity against hCA XII (*K*_i_ = 4.9 nM) with selectivity over hCA I, II, and IX (SI = 628.4, 12.8, and 11.9, respectively), both outperforming the standard inhibitor acetazolamide. SAR analysis indicated that electron withdrawing groups, particularly cyano and bromo substituents on the indole scaffold, enhanced selectivity toward tumor-associated hCA IX and XII. A clear positional effect was observed in which substitution at the 5-position of the indole core favors hCA IX inhibition whereas substitution at the 6-position promotes hCA XII selectivity.

Molecular docking studies predicted that the top two lead compounds (**15h** and **15j**) exhibited the typical coordinate bond with Zn^2+^ which was further stabilized by the presence of hydrogen bonds with key active site residues. MD simulations were employed to validate the stability of these predicted interactions where ligand/protein complexes showed reduced RMSD values and sustained key contacts throughout the simulation. In silico ADME calculation suggested that compound **15j** possesses a more favorable balance of lipophilicity and polarity, indicating improved membrane permeability when compared to compound **15h**.

Despite these promising results, the present study is limited to enzyme-based assays and computational analysis and therefore does not yet confirm whether the observed selectivity translates into anticancer activity in biological systems. In addition, the absence of experimentally determined co-crystal structures limits definitive validation of the proposed binding modes. Furthermore, the predicted ADME properties have not been experimentally verified. To address these limitations, our future studies will focus on evaluating the anticancer efficacy of these compounds in cancer cell lines overexpressing hCA IX and XII and on validating their isoform selective effects under cellular conditions. In parallel, pharmacokinetic and metabolic studies will be conducted to confirm the predicted drug like properties. Further structural optimization based on the identified SAR trends will also be pursued to improve both potency and selectivity.

Overall, compounds **15h** and **15j** represent promising lead candidates and the indolylchalcone benzenesulfonamide scaffold provides a useful platform for the rational development of isoform selective carbonic anhydrase inhibitors.

## Supplementary Information

Below is the link to the electronic supplementary material.

Supplementary Material 1

Supplementary Material 2

## Data Availability

Data is provided within the manuscript and/or the supplementary information file.
