# Supplementary material for: Discovery of indolylchalcone benzenesulfonamides as selective inhibitors of tumor-associated carbonic anhydrase IX and XII
Source: Sci Rep. 2026 Jul 8;16:21192. doi: 10.1038/s41598-026-49231-6 (PMC13346758; doi:10.1038/s41598-026-49231-6)
Supplement: Supplementary file 2 — Supplementary Material 2 [file 41598_2026_49231_MOESM2_ESM.docx]

**Supporting Information**

**1. NMR spectrum**


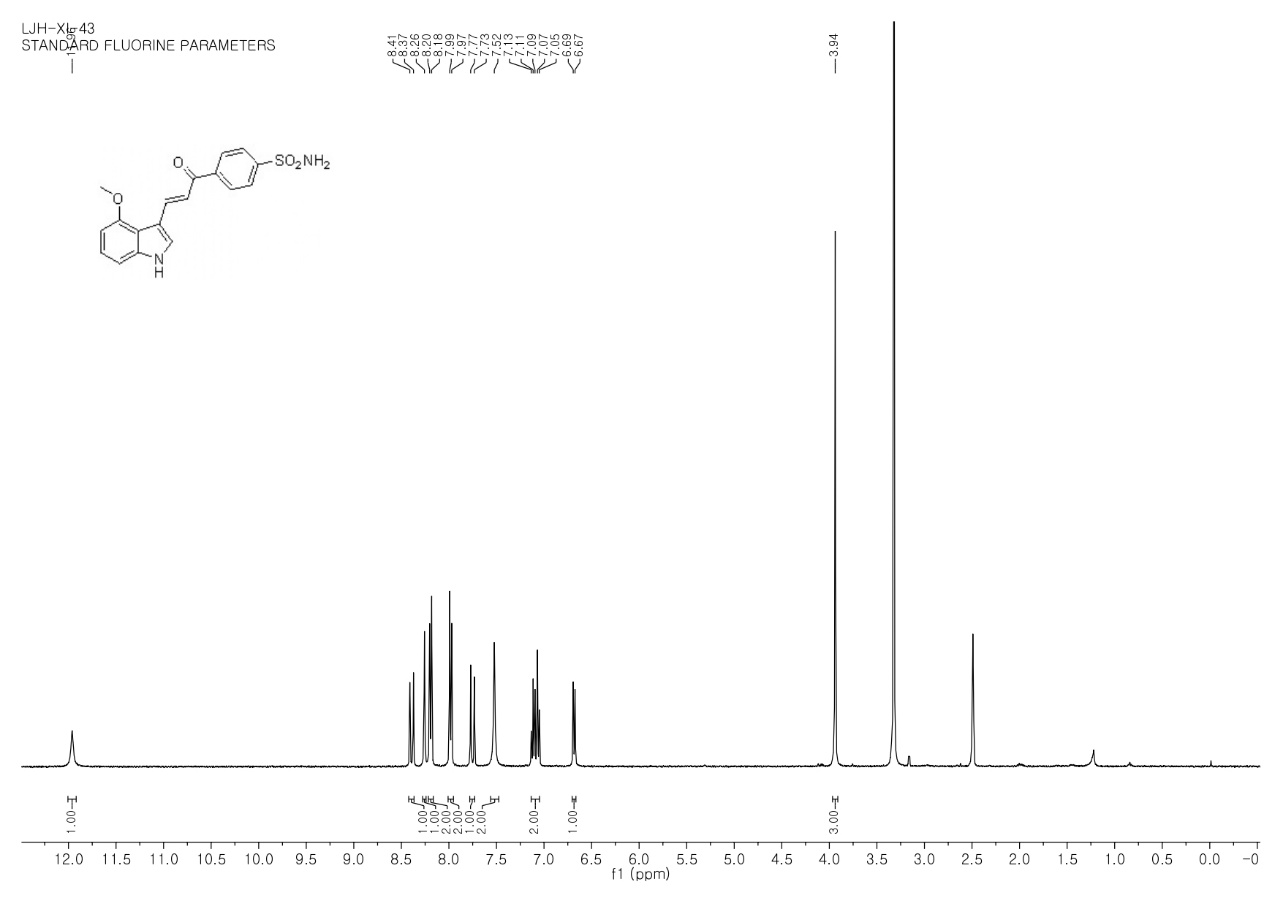


^1^H NMR spectrum of **15a**


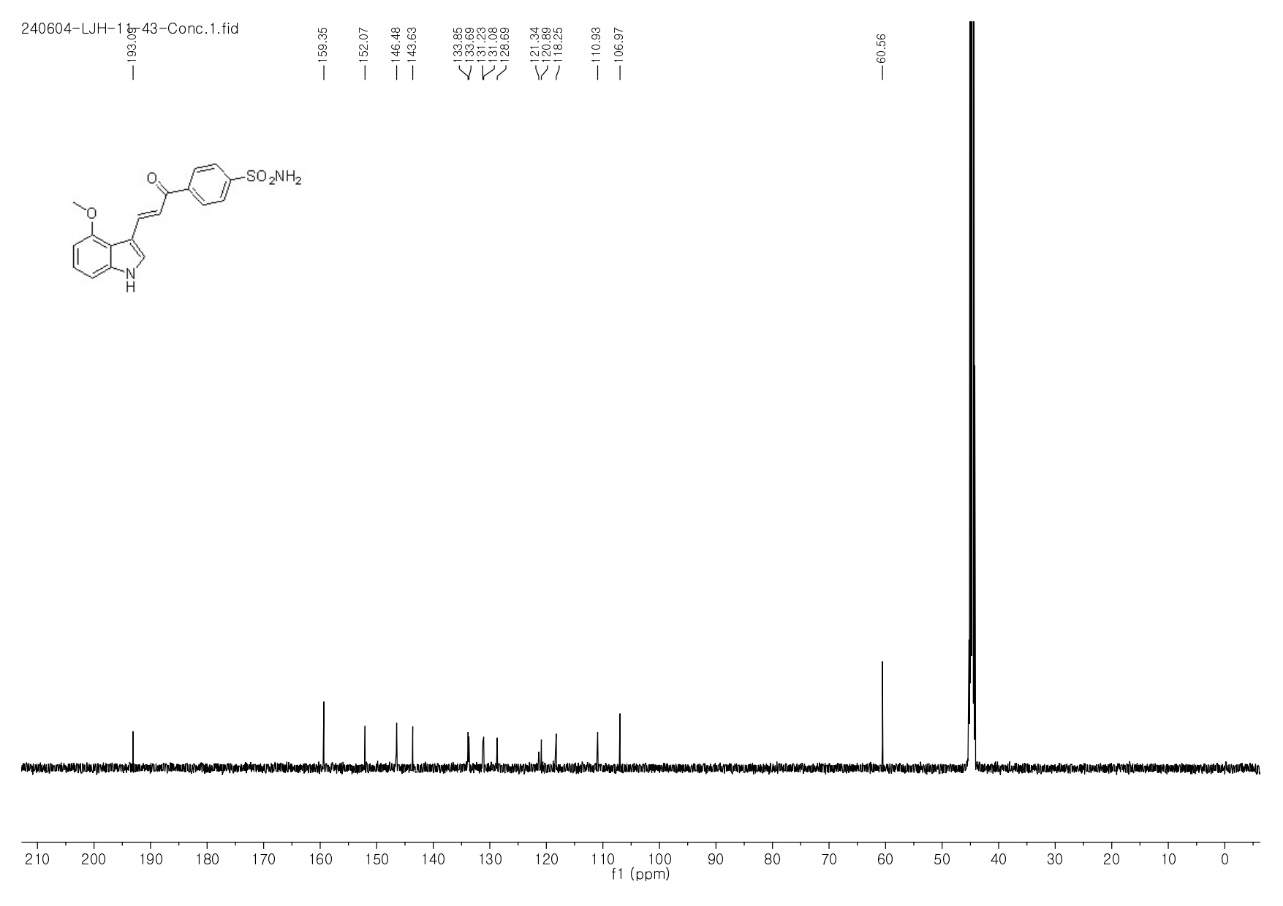


^13^C NMR spectrum of **15a**


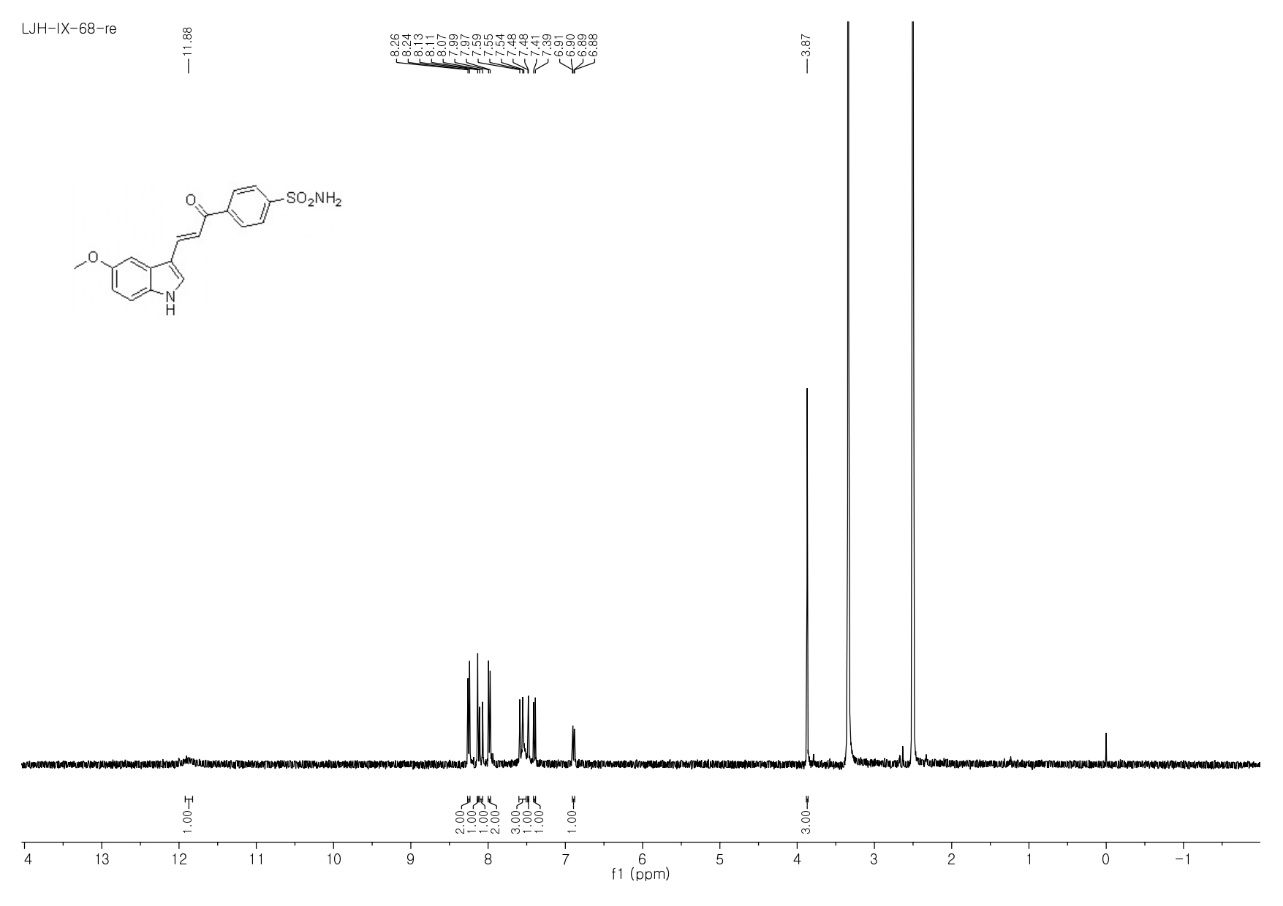


^1^H NMR spectrum of **15b**


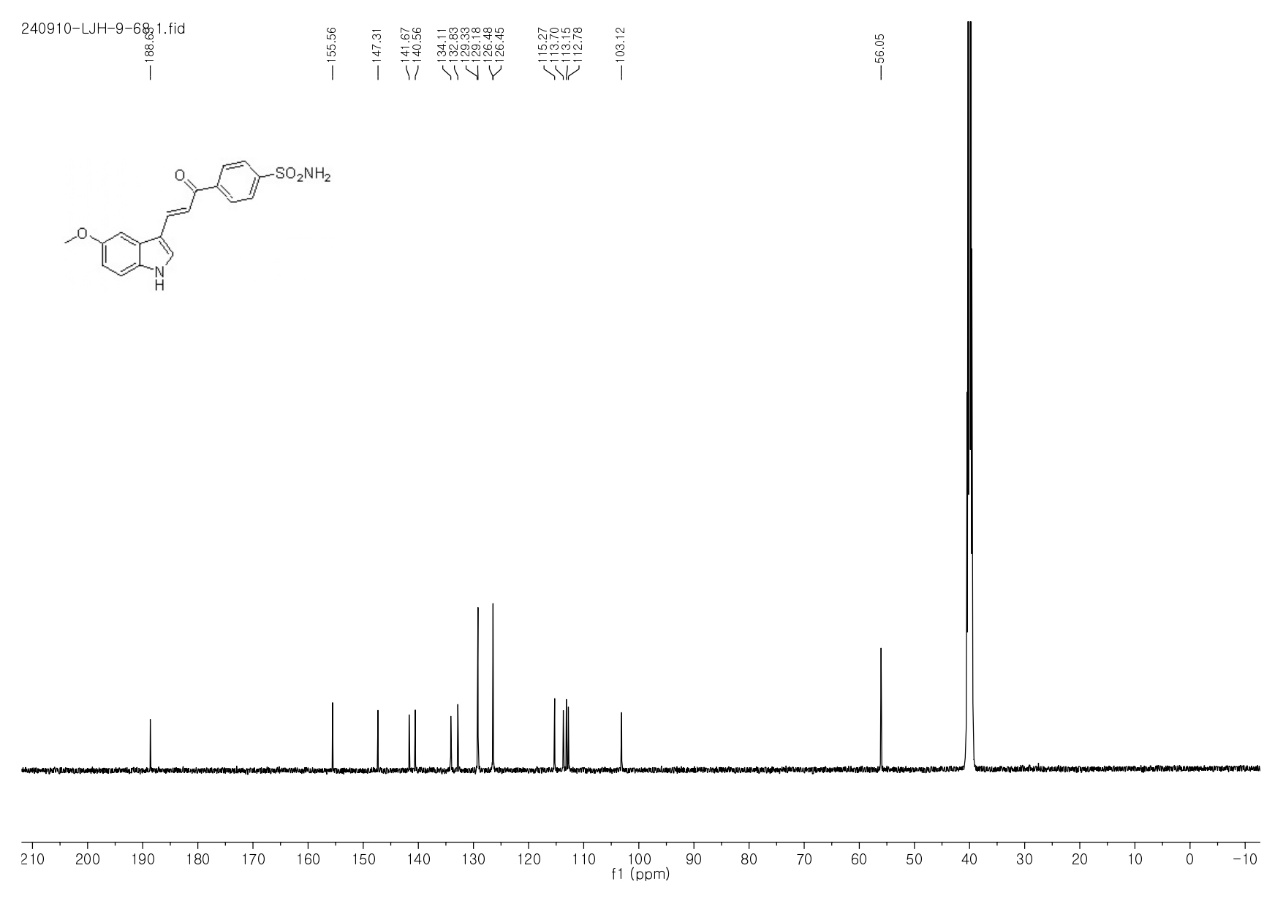


^13^C NMR spectrum of **15b**


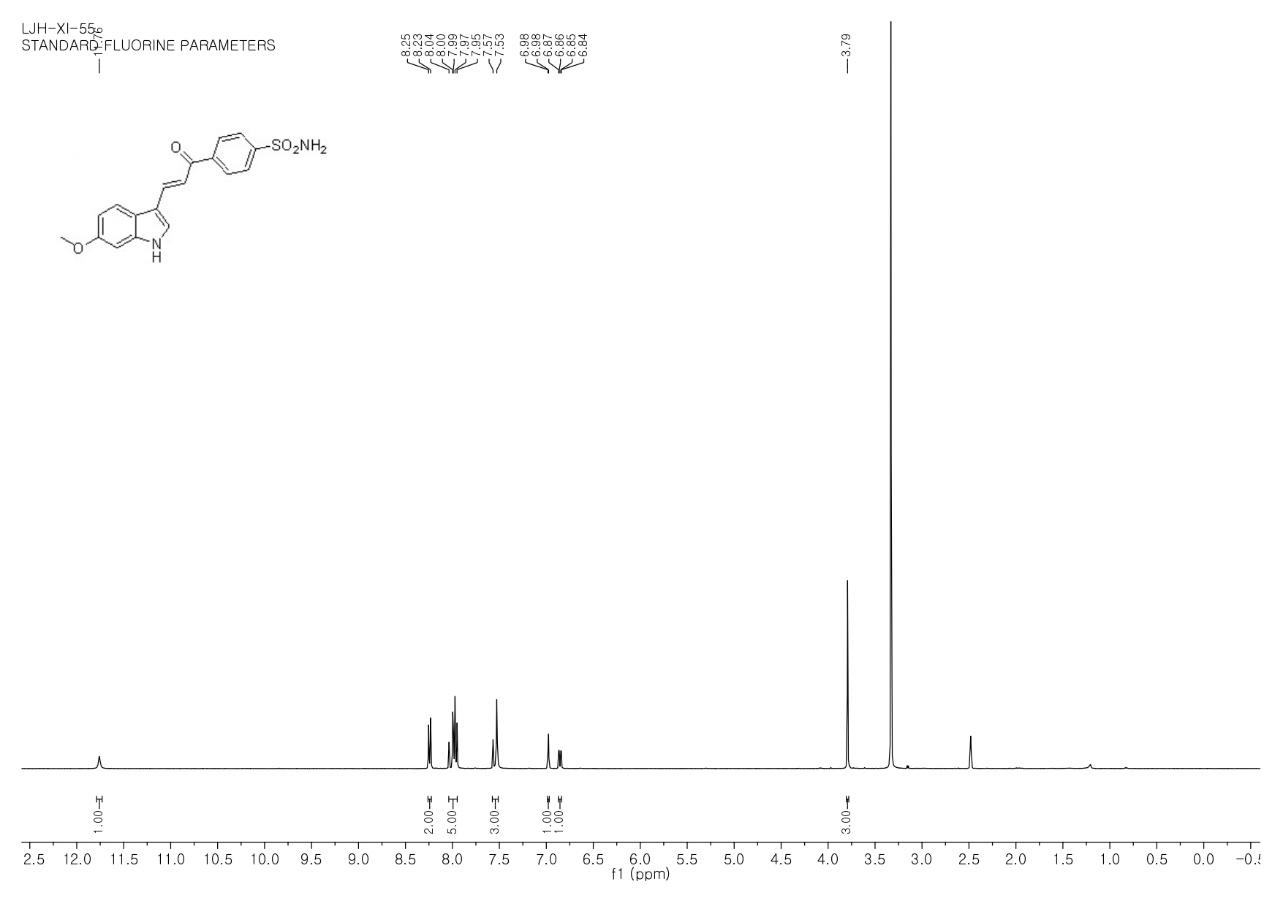


^1^H NMR spectrum of **15c**


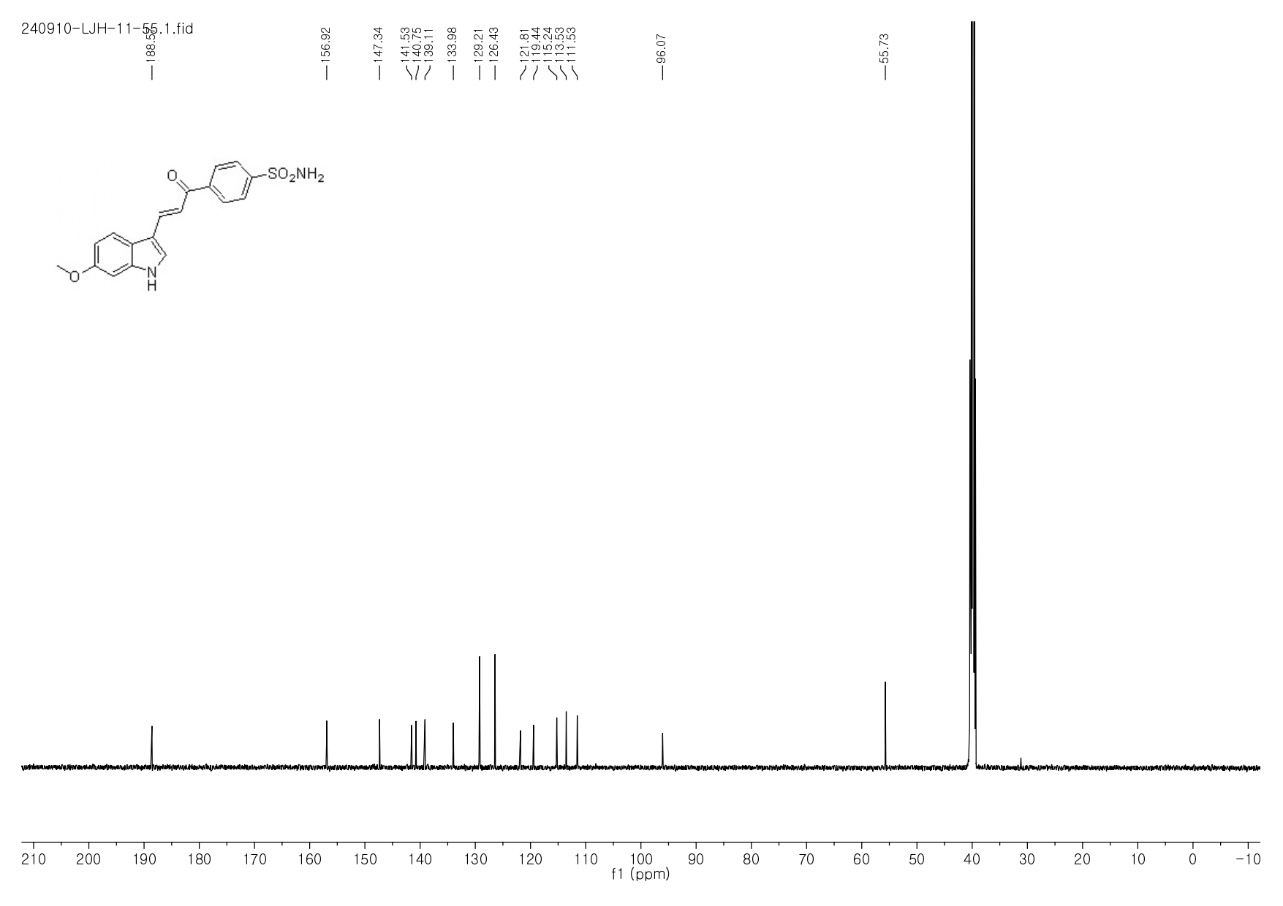


^13^C NMR spectrum of **15c**


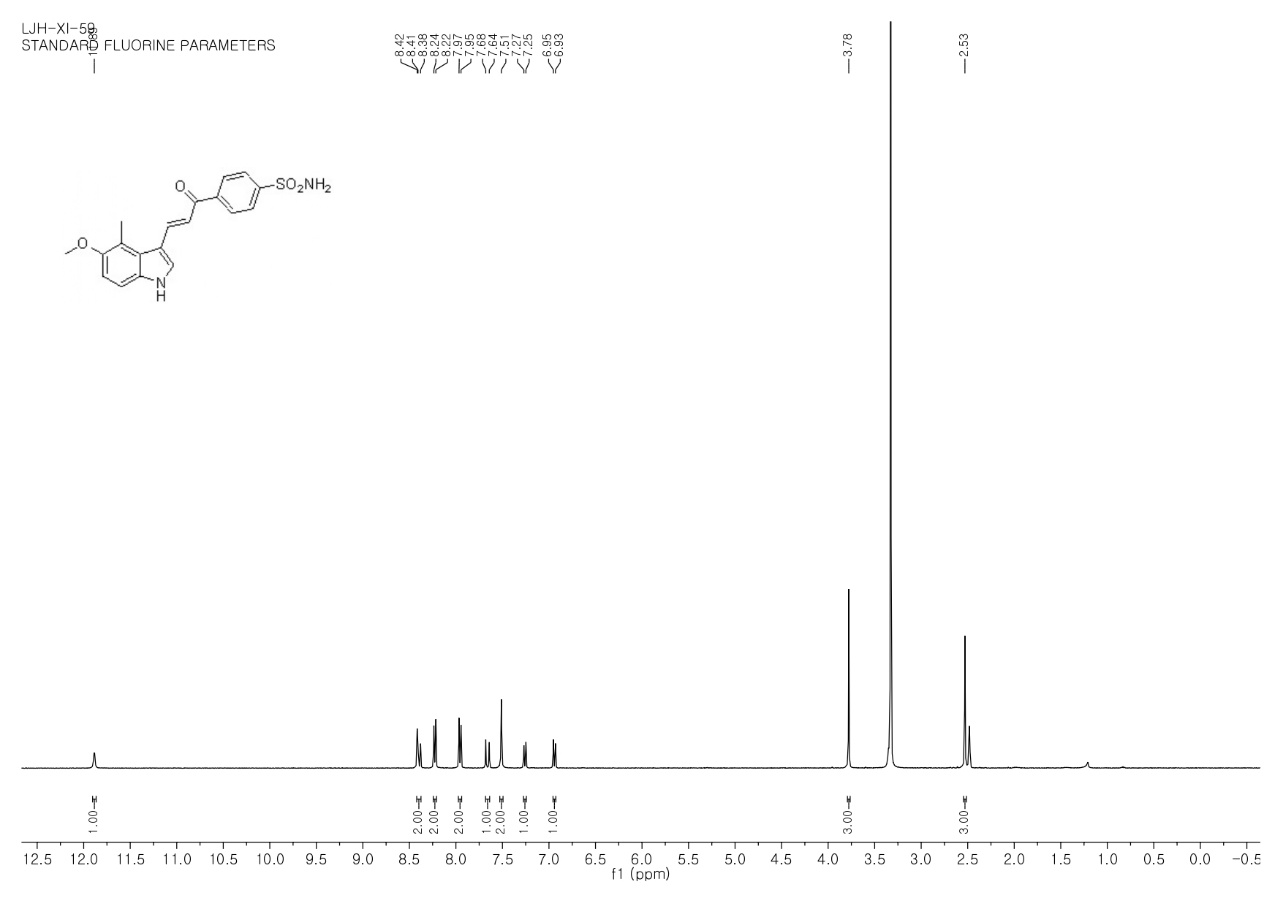


^1^H NMR spectrum of **15d**


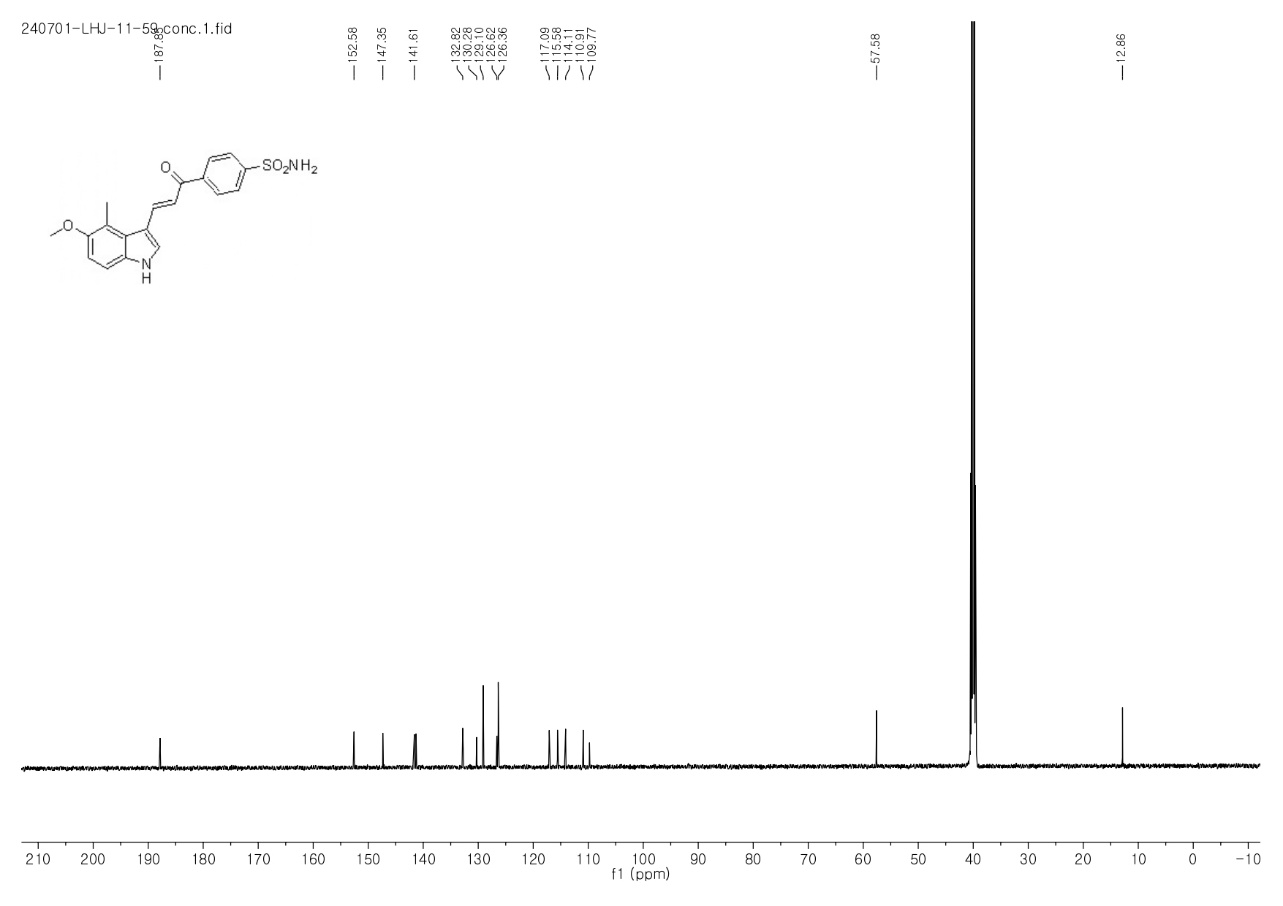


^13^C NMR spectrum of **15d**


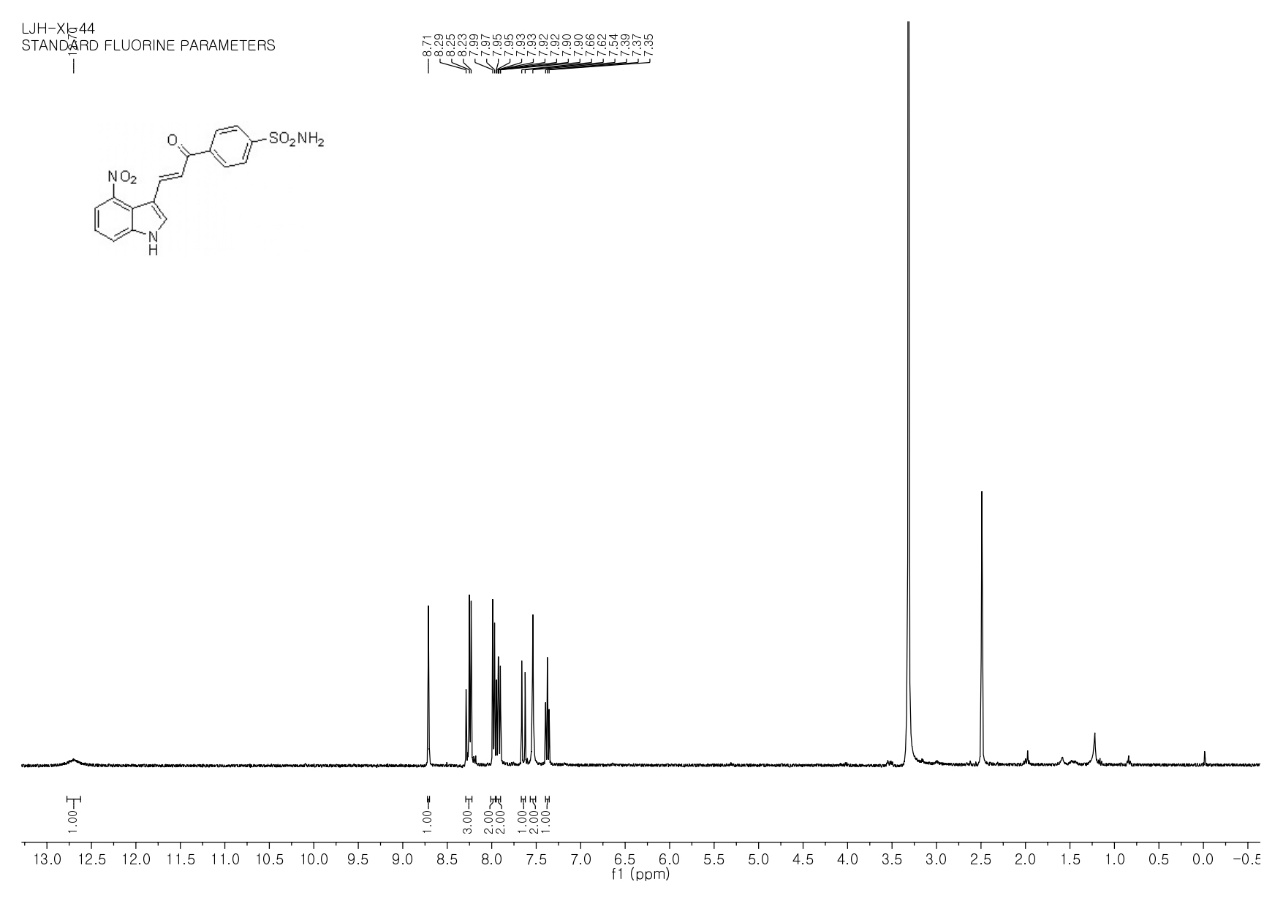


^1^H NMR spectrum of **15e**


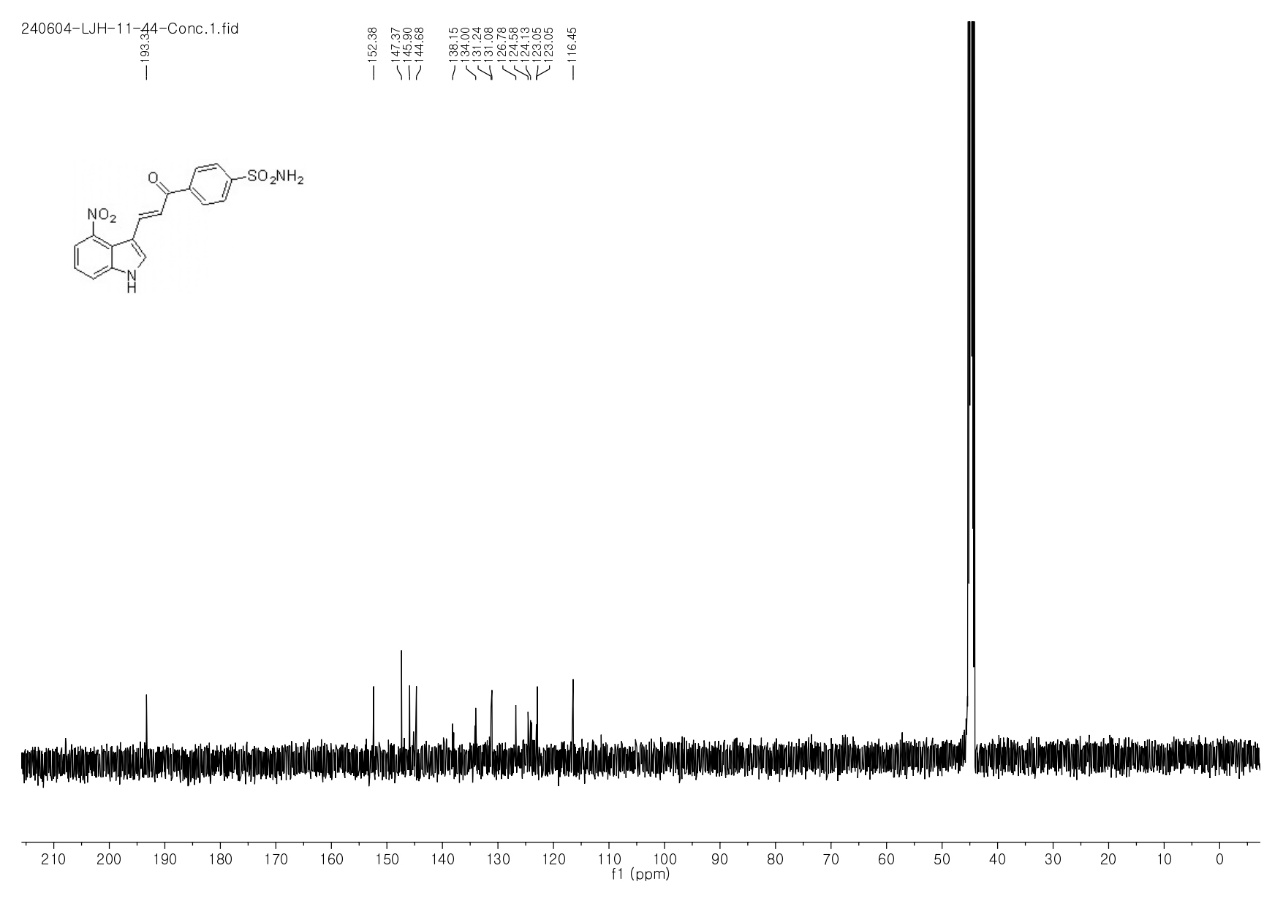


^13^C NMR spectrum of **15e**


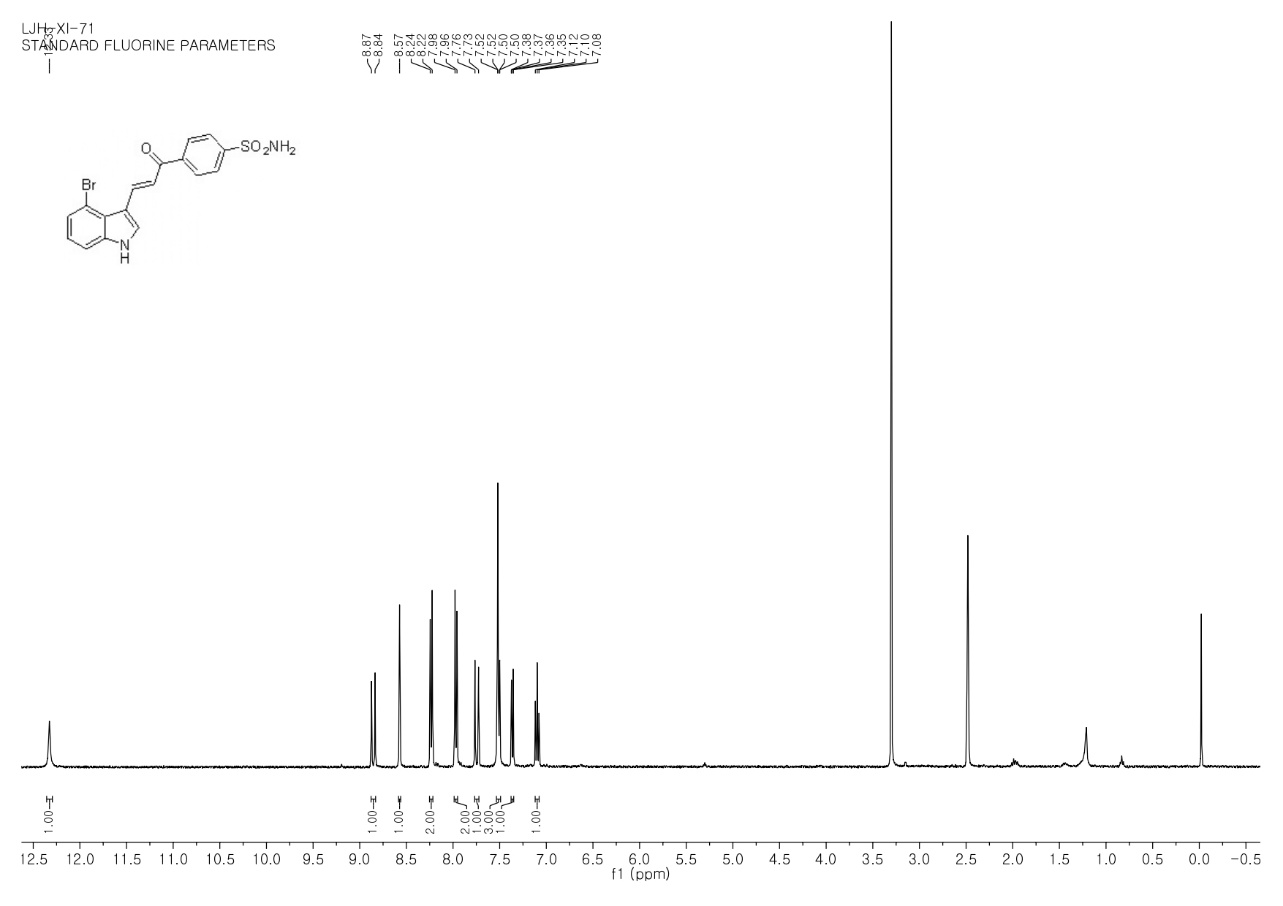


^1^H NMR spectrum of **15f**


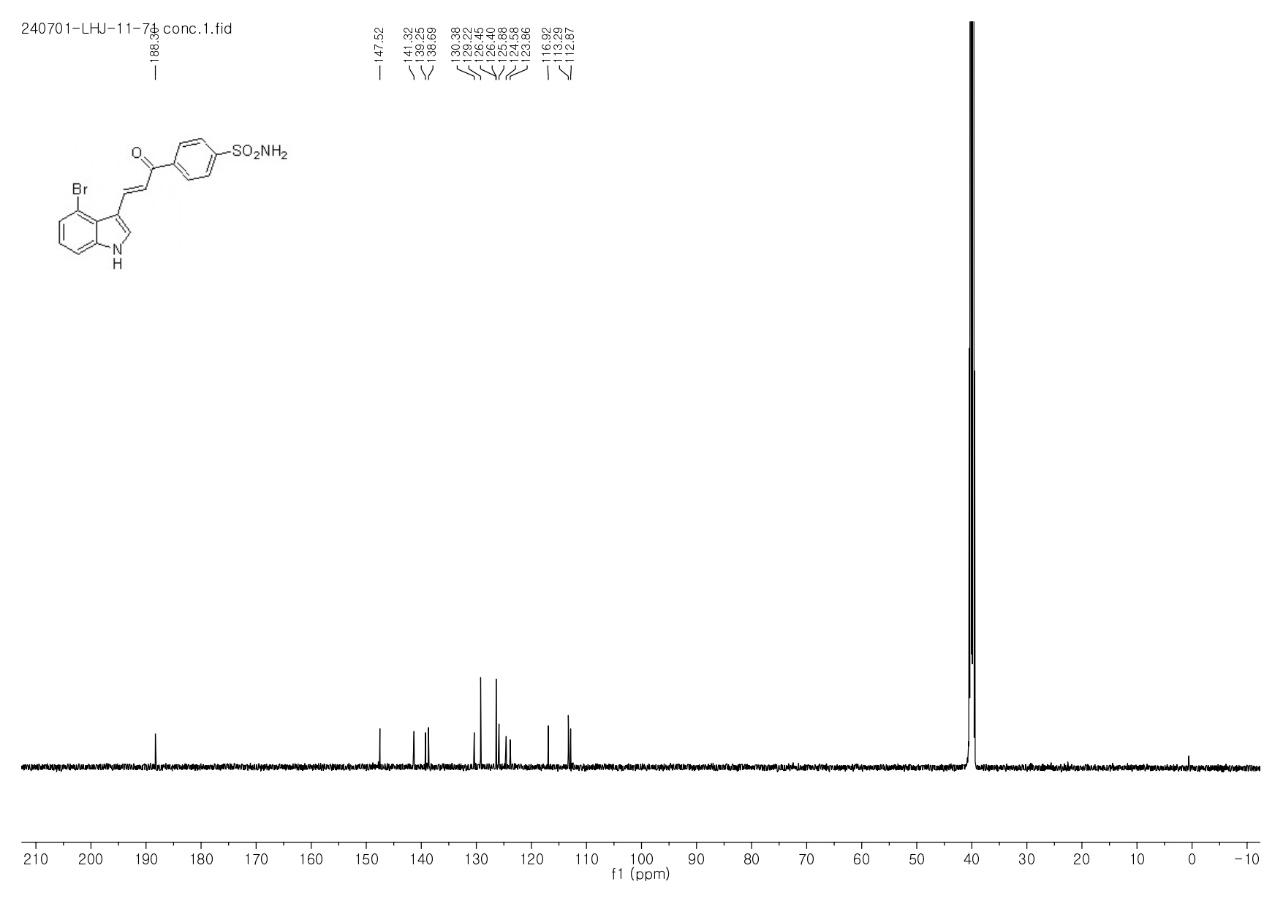


^13^C NMR spectrum of **15f**


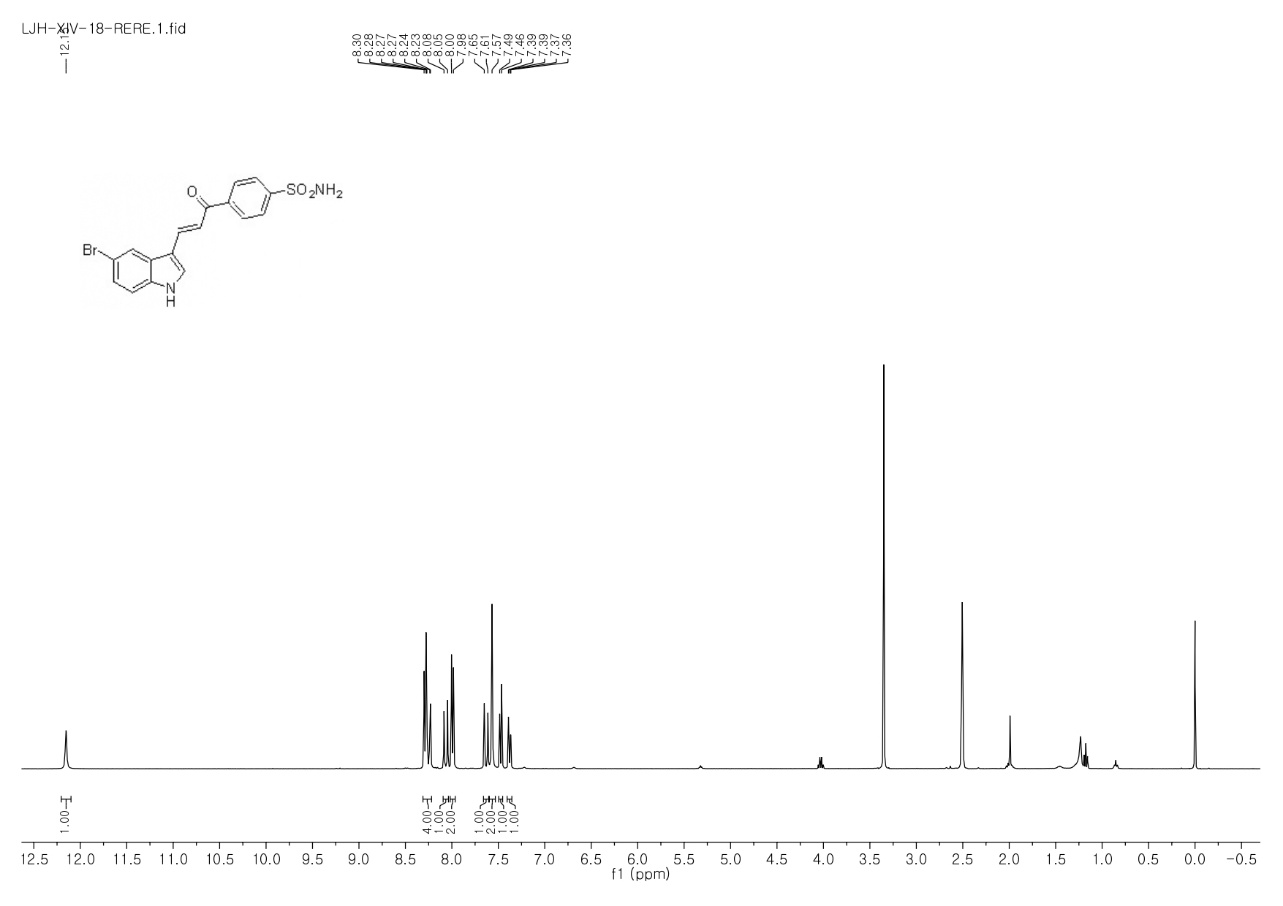


^1^H NMR spectrum of **15g**


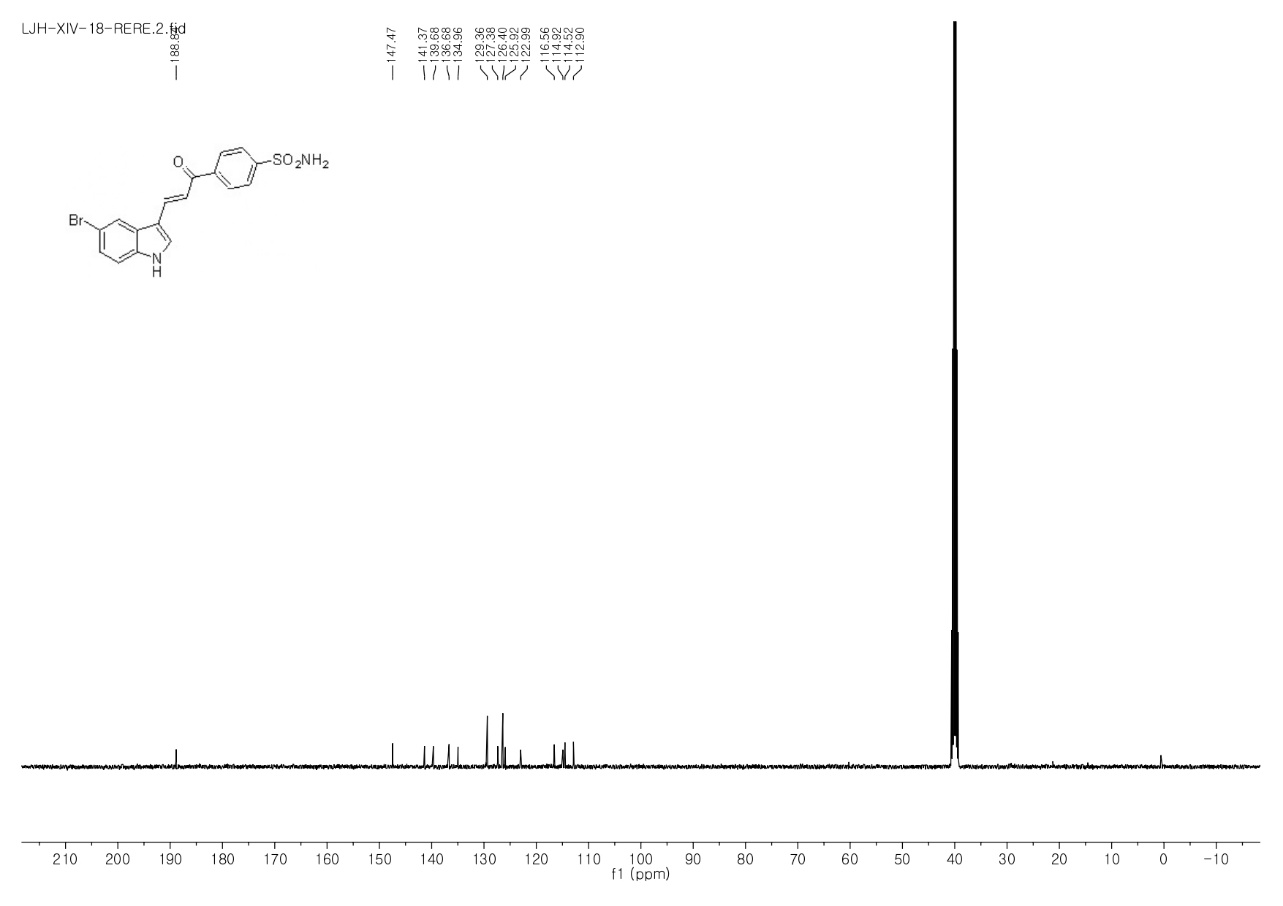


^13^C NMR spectrum of **15g**


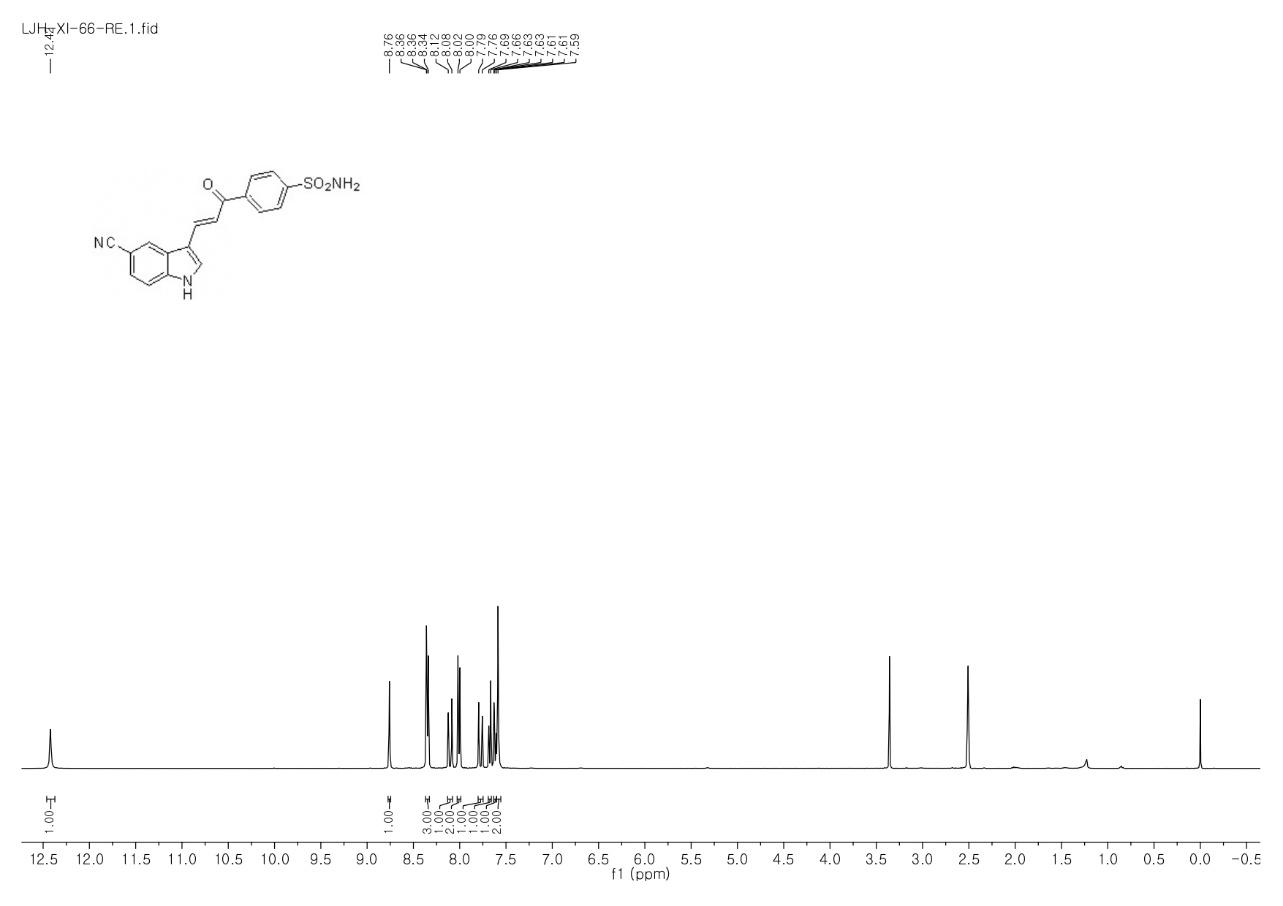


^1^H NMR spectrum of **15h**


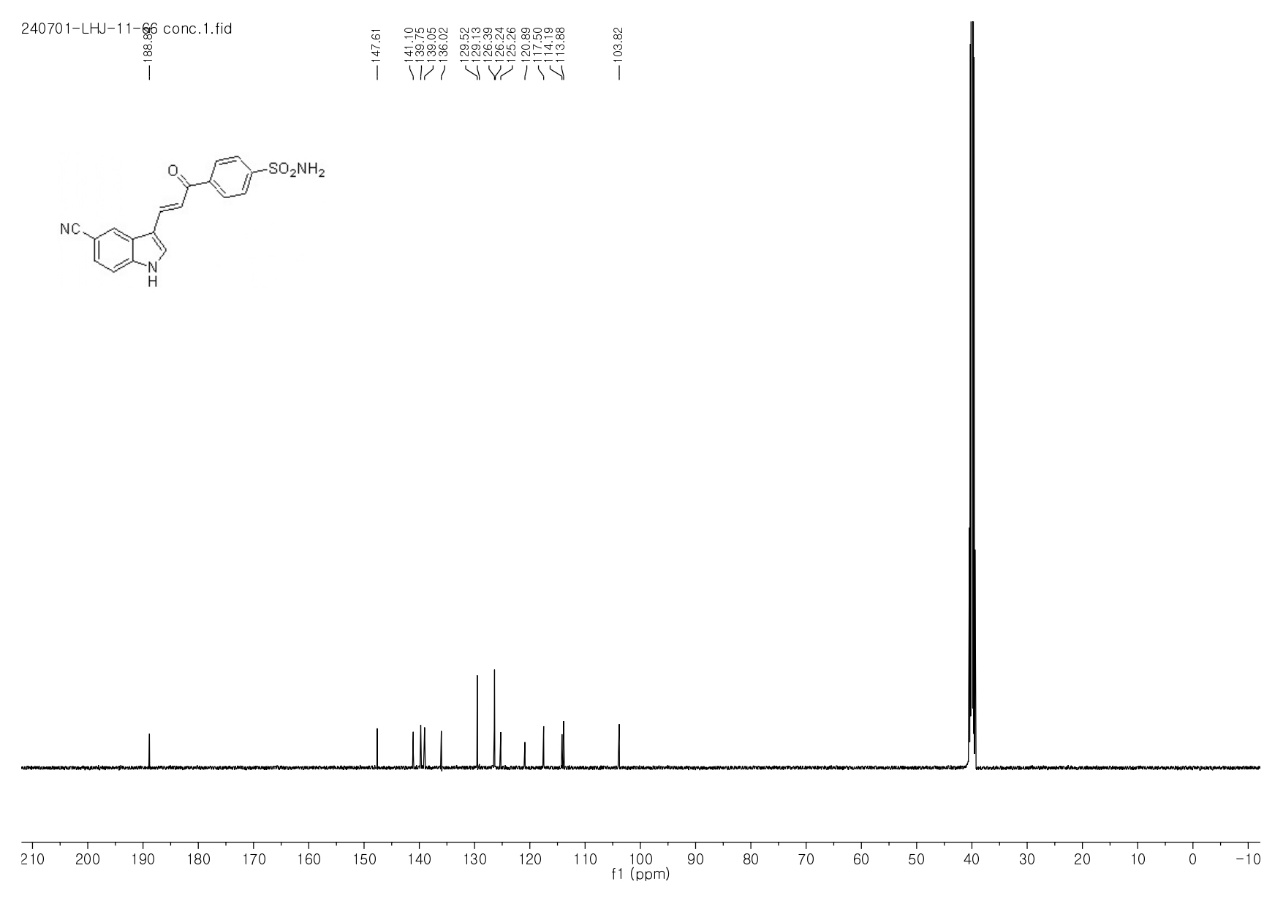


^13^C NMR spectrum of **15h**


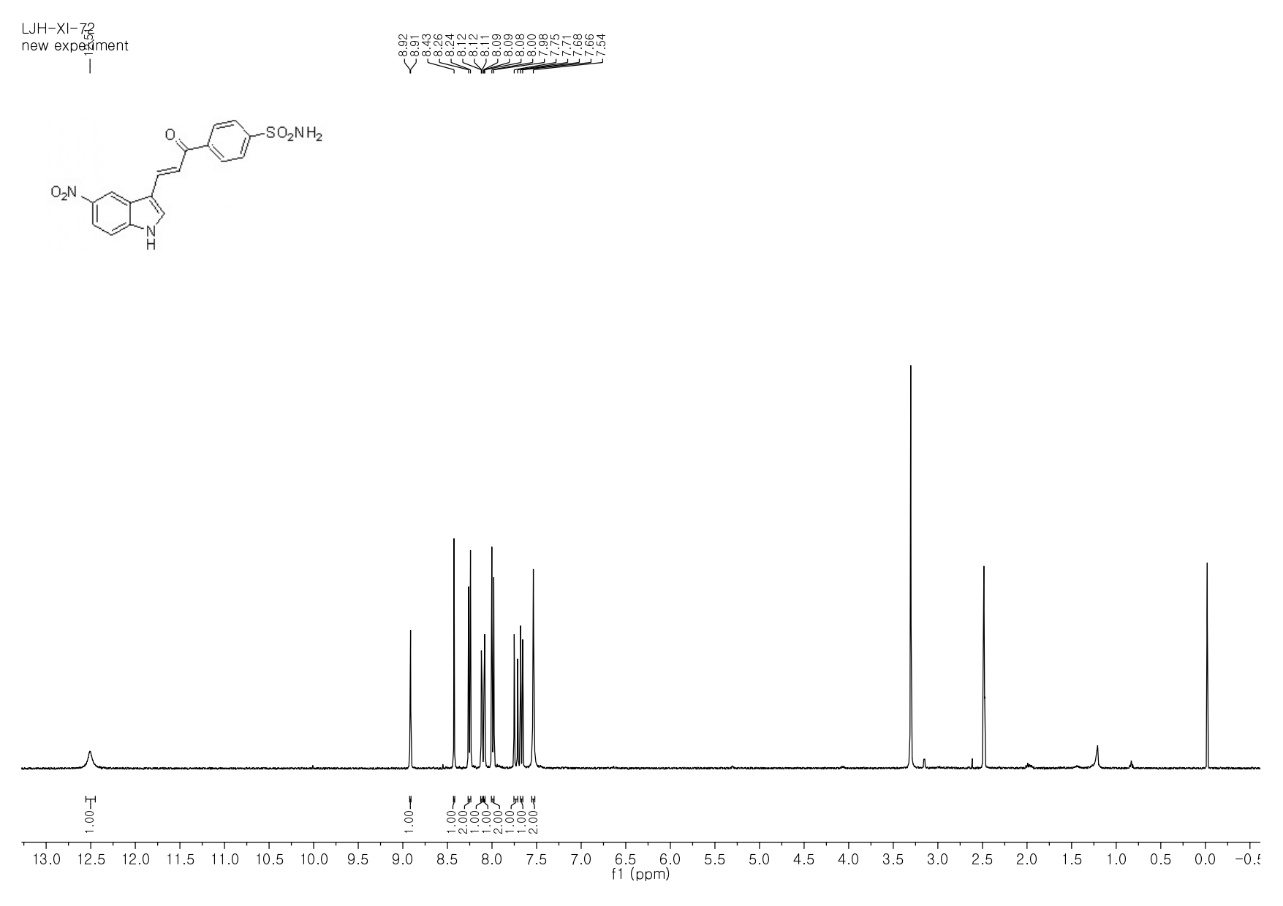


^1^H NMR spectrum of **15i**


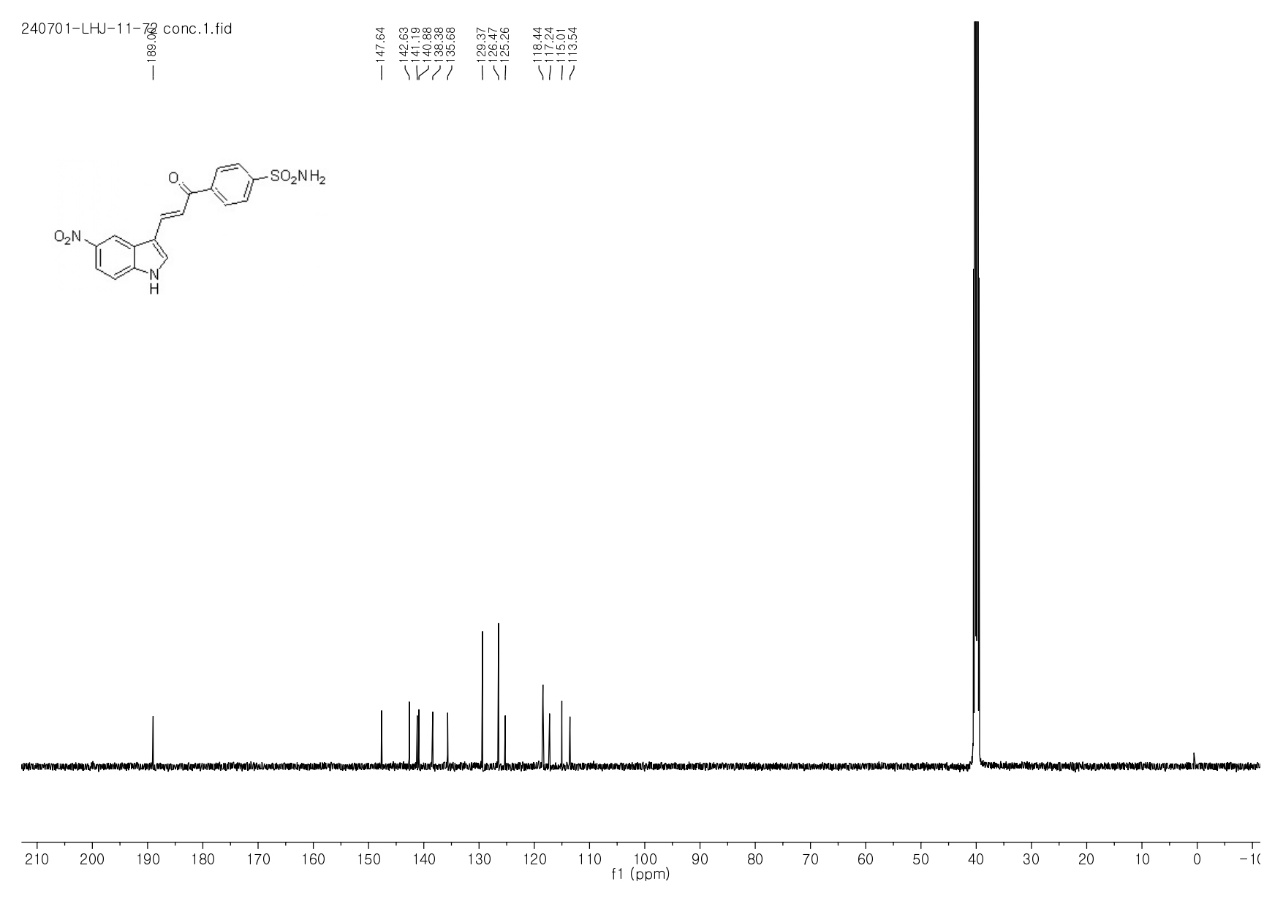


^13^C NMR spectrum of **15i**


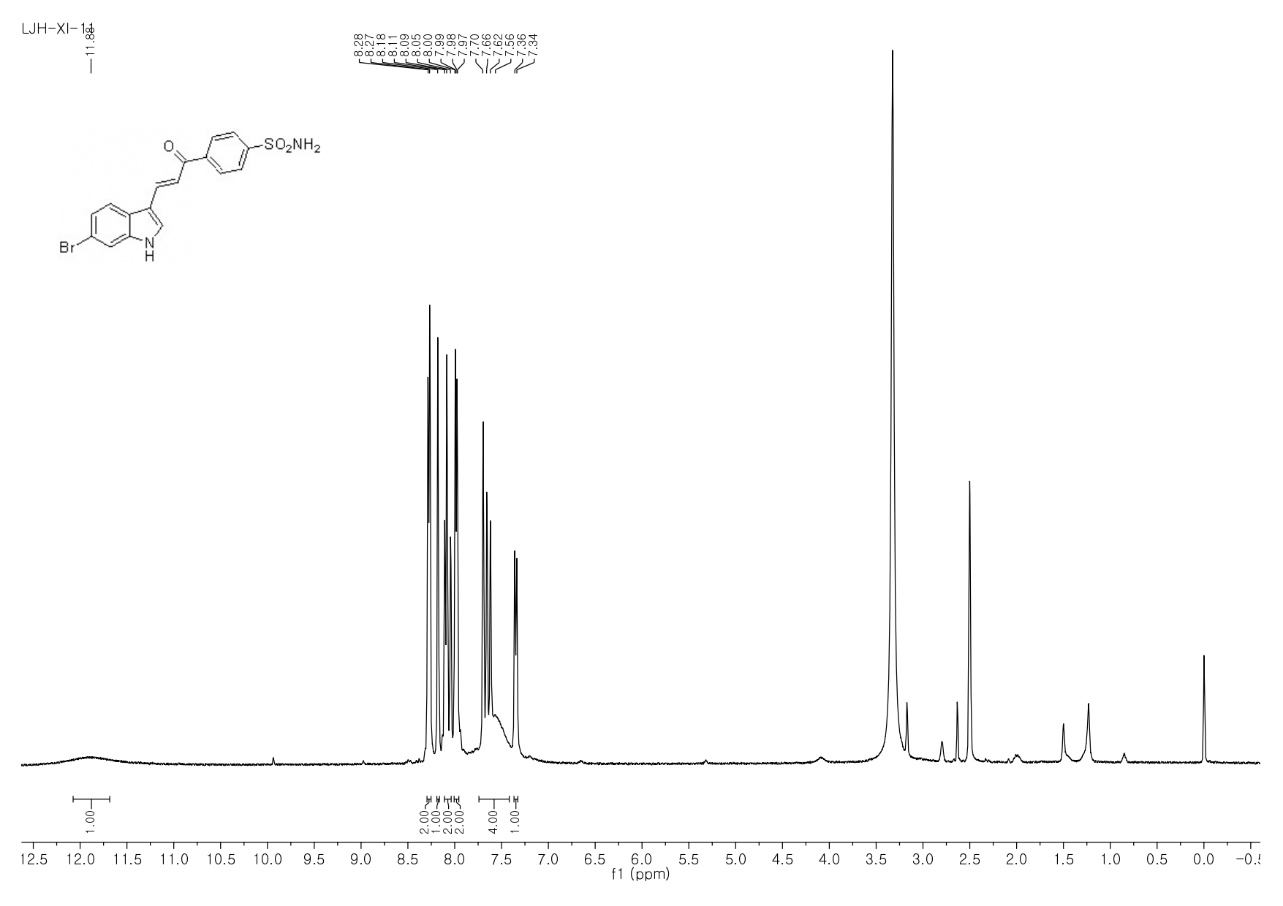


^1^H NMR spectrum of **15j**


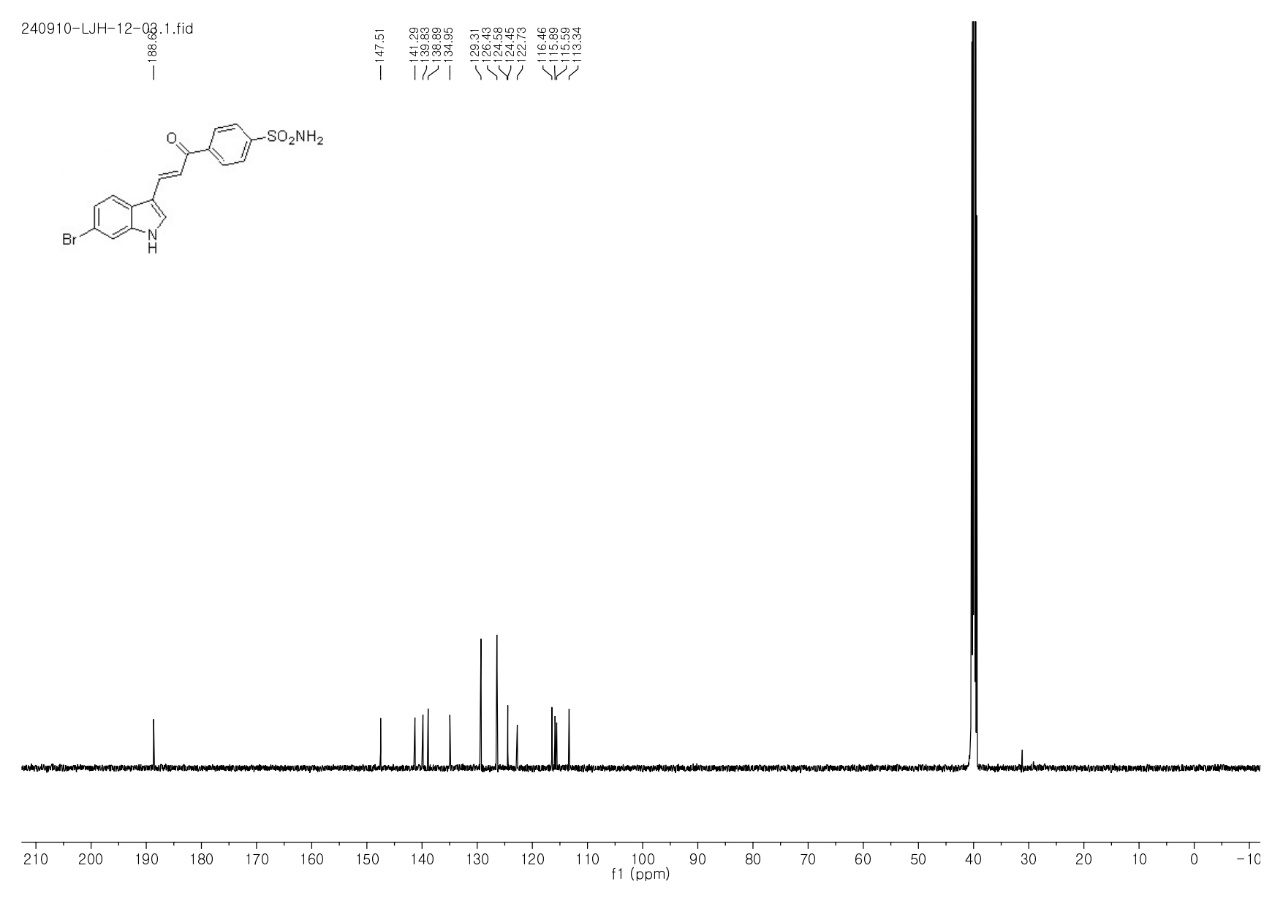


^13^C NMR spectrum of **15j**


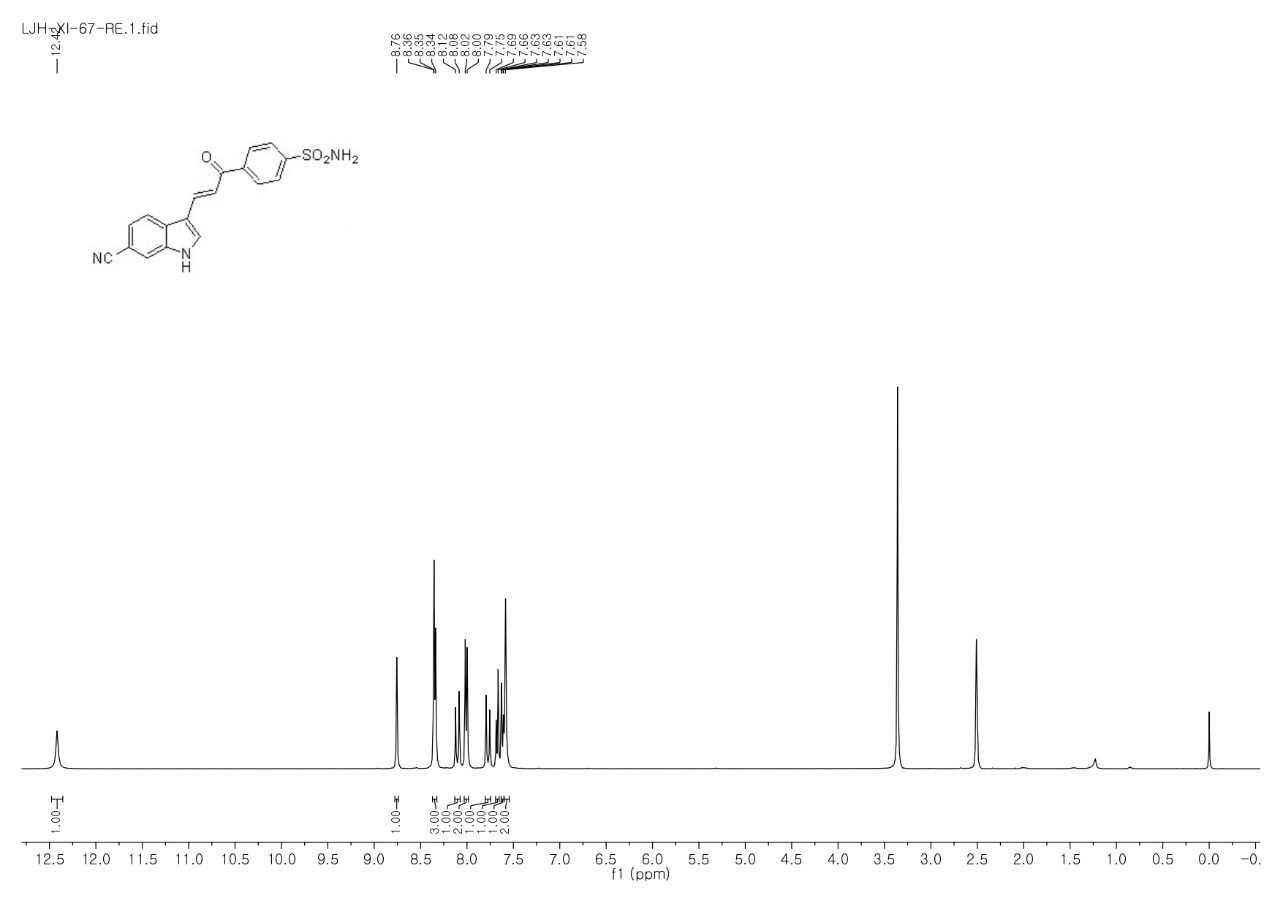


^1^H NMR spectrum of **15k**


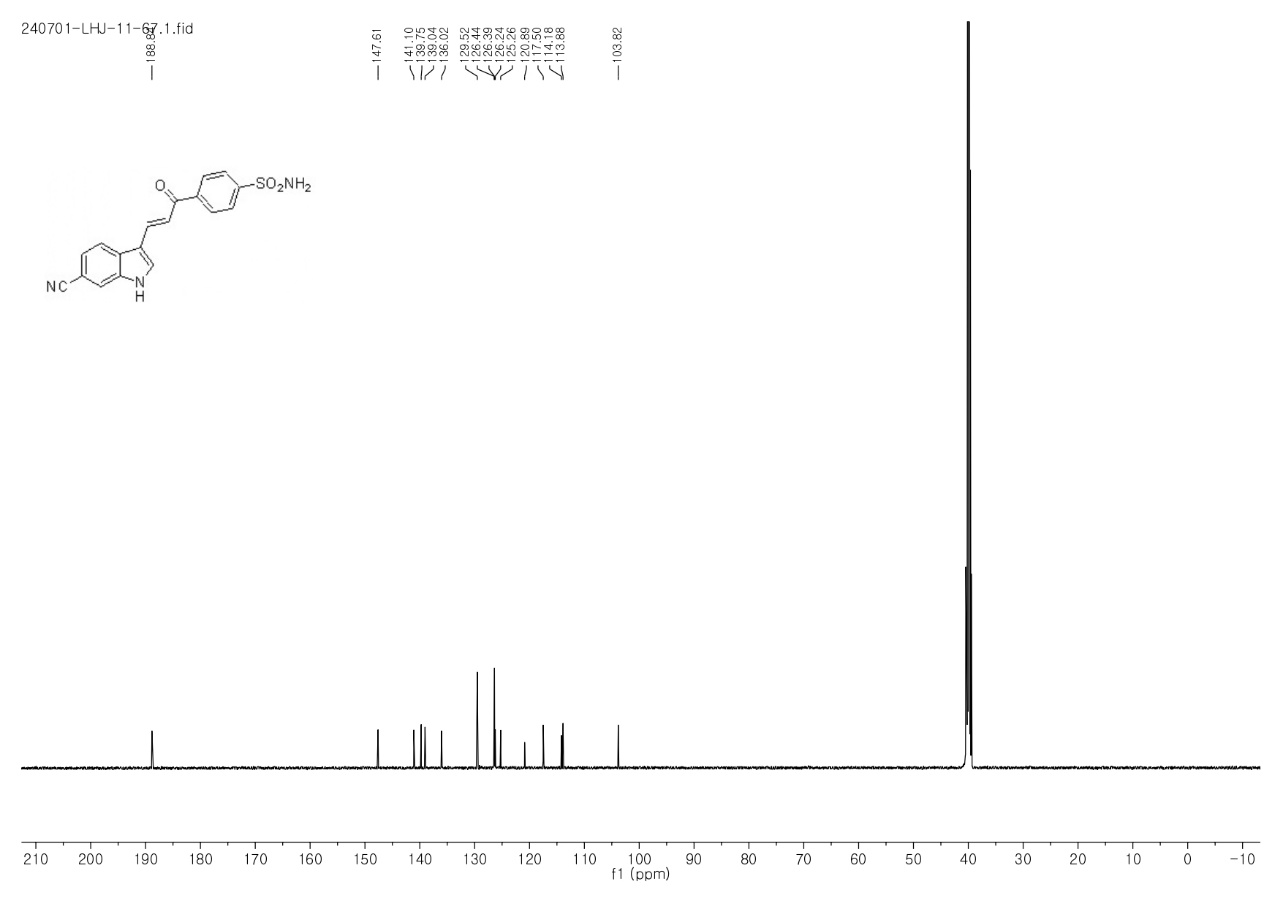


^13^C NMR spectrum of **15k**


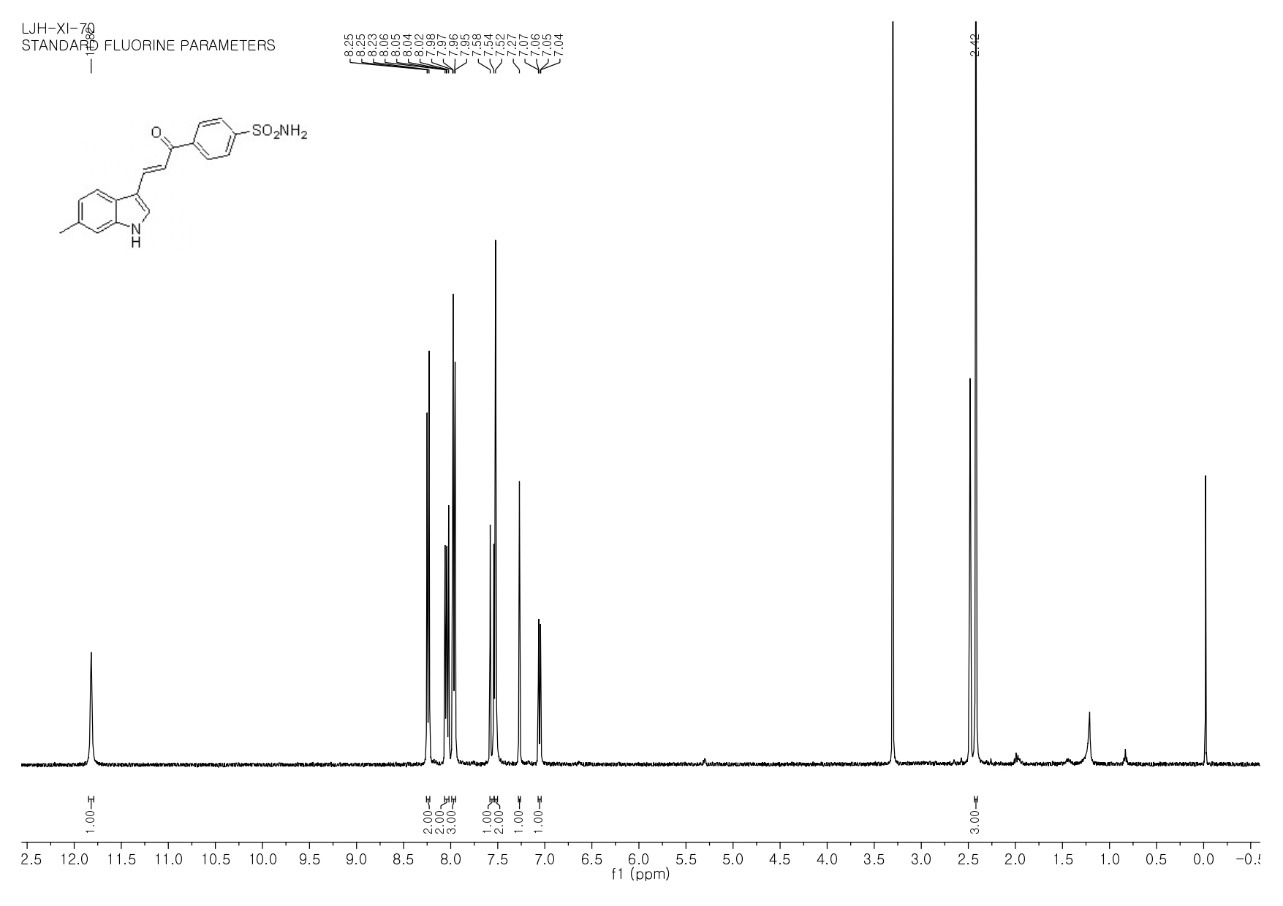


^1^H NMR spectrum of **15l**


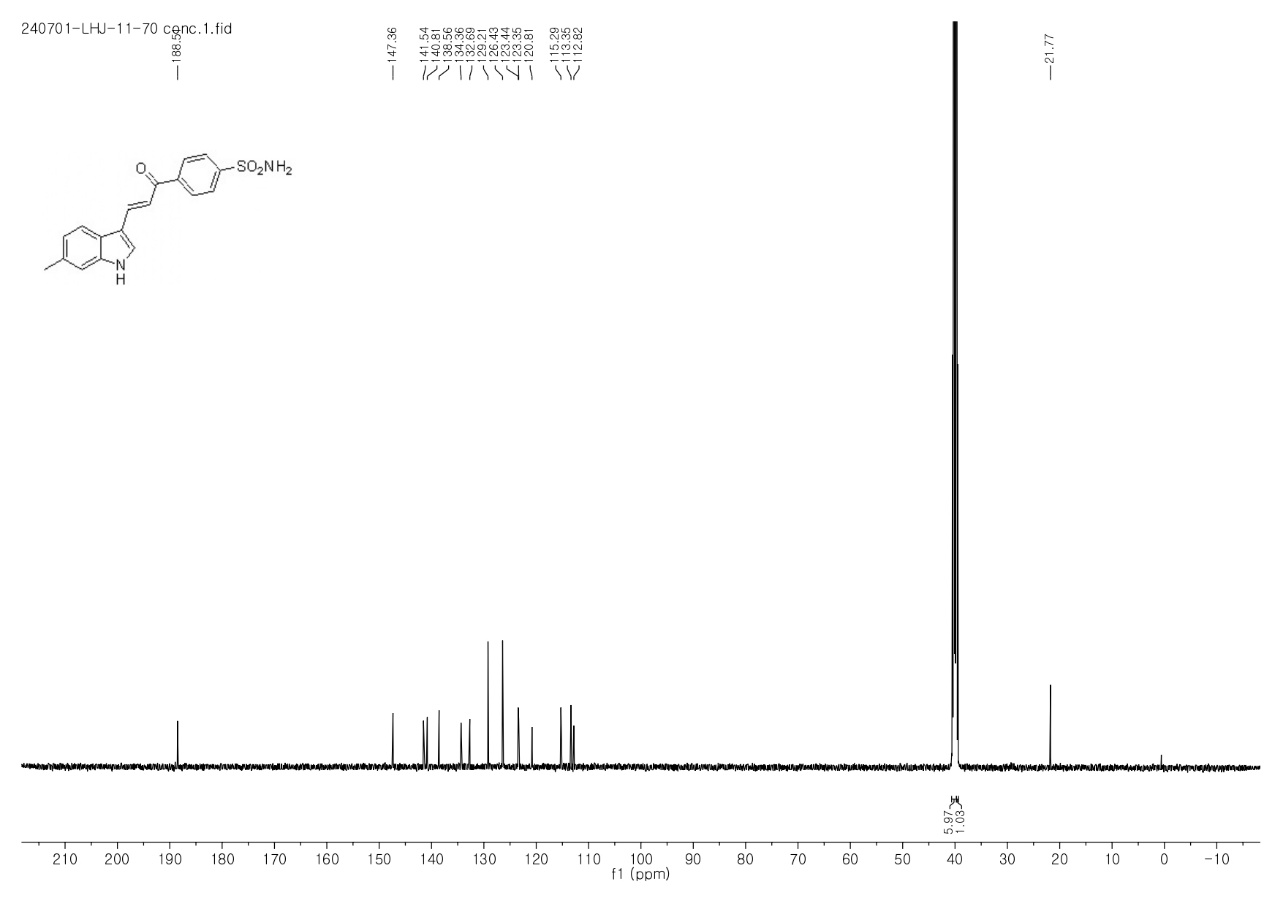


^13^C NMR spectrum of **15l**


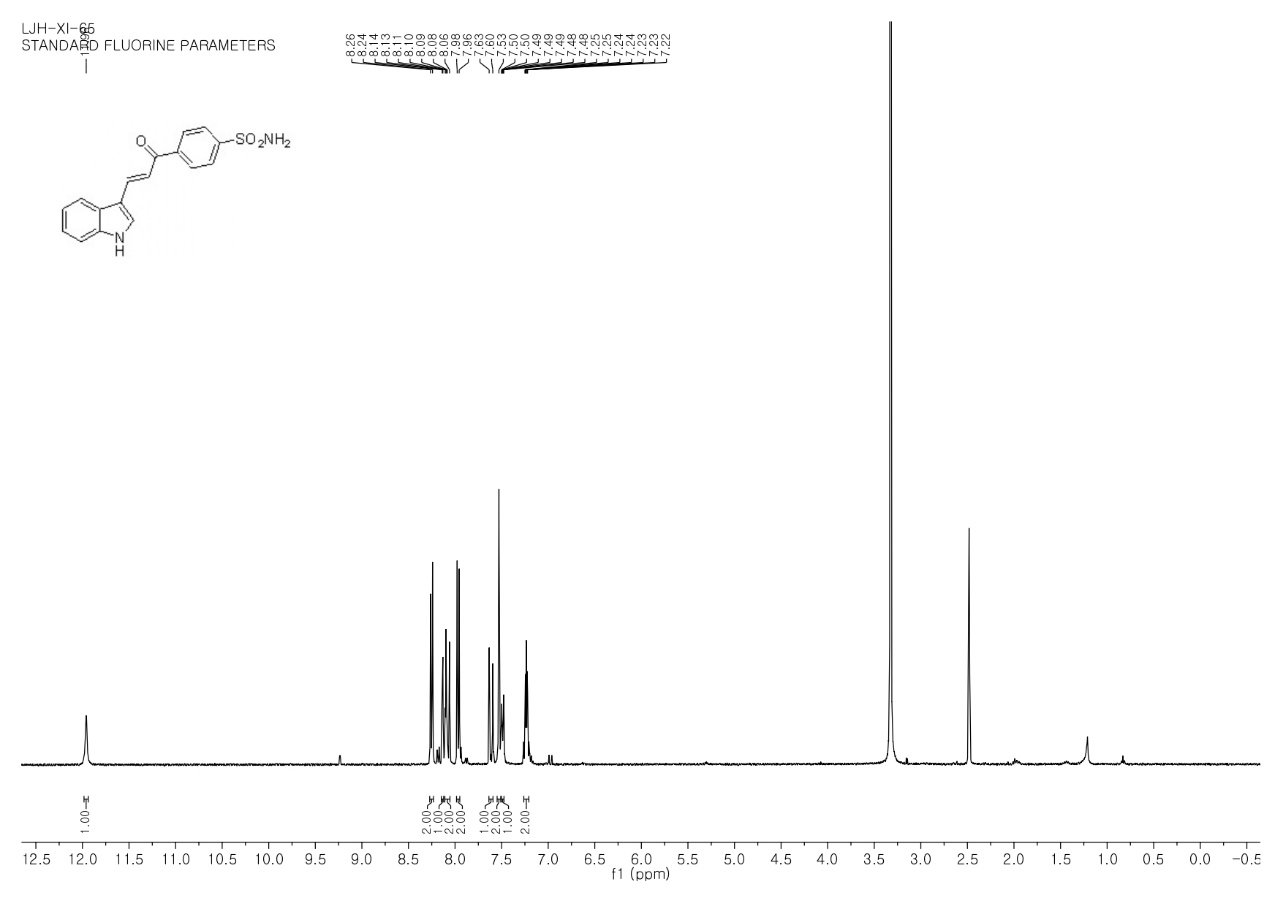


^1^H NMR spectrum of **15m**


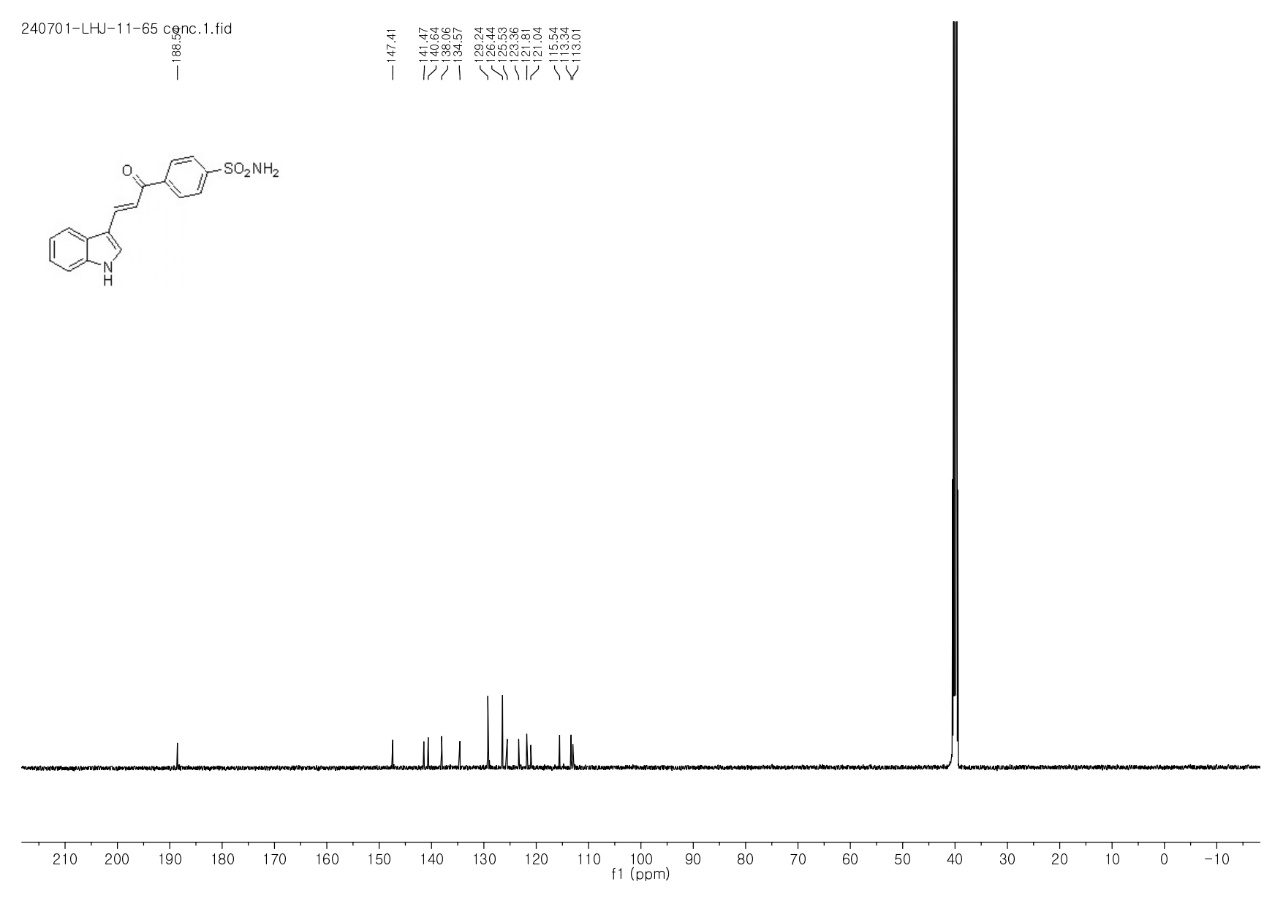


^13^C NMR spectrum of **15m**


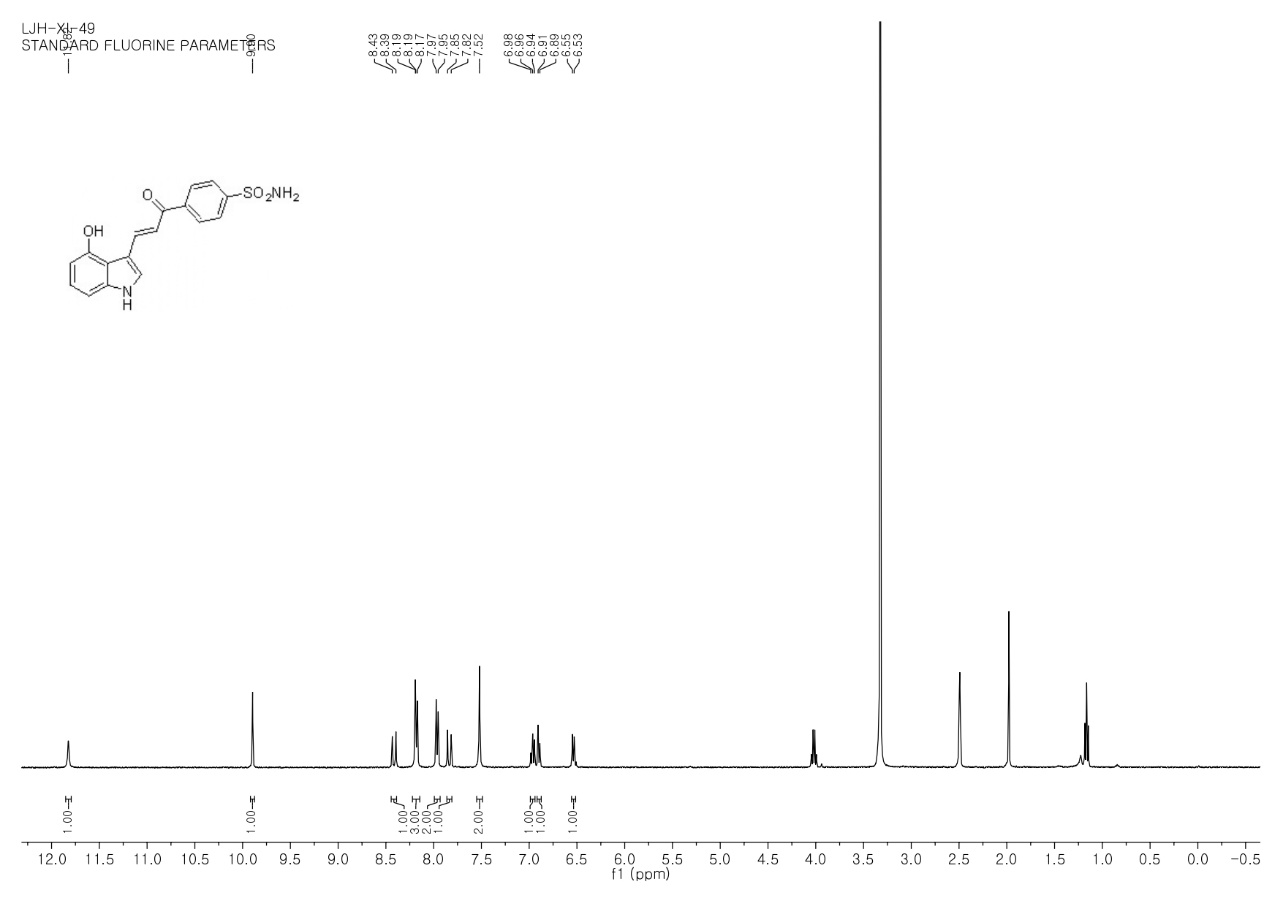


^1^H NMR spectrum of **16a**


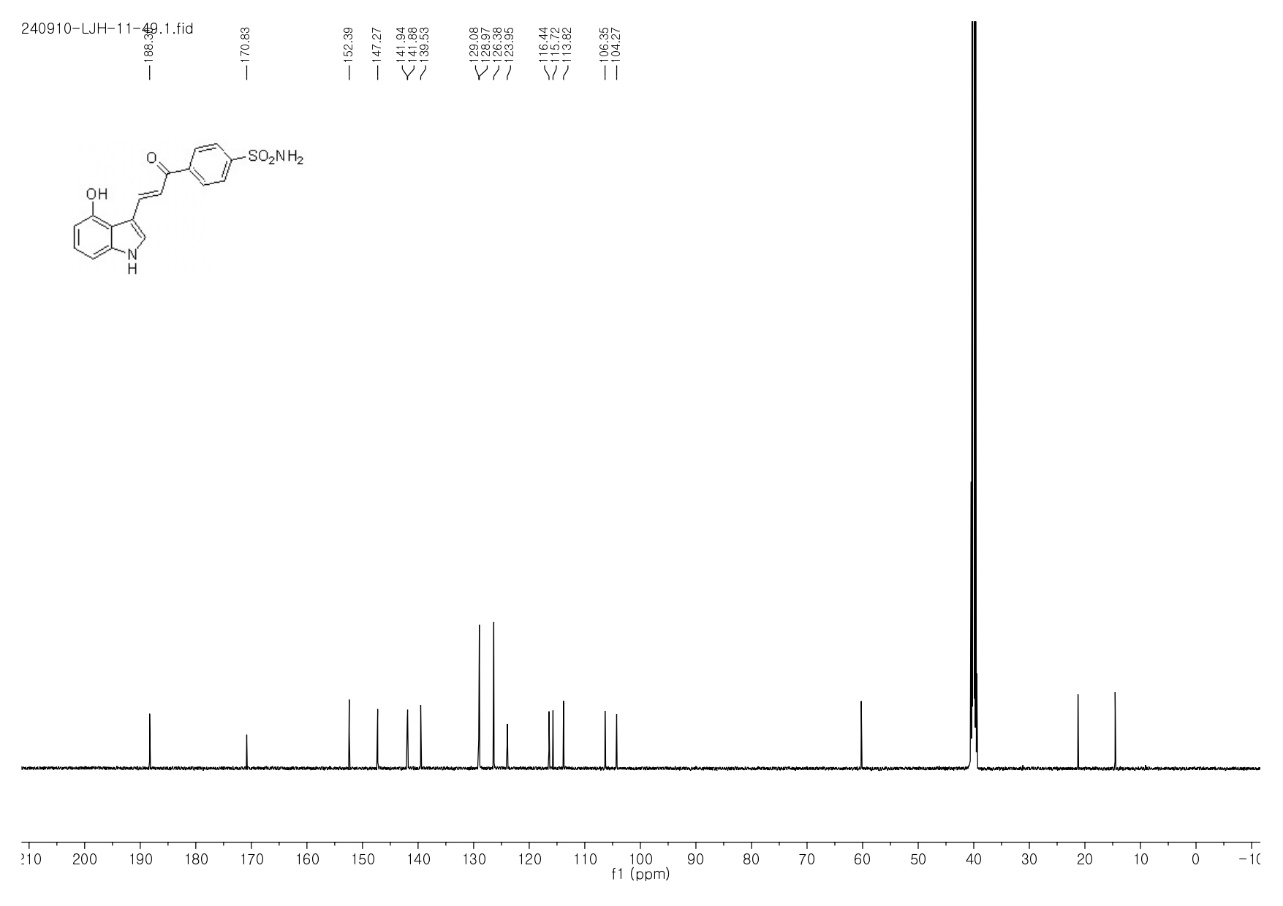


^13^C NMR spectrum of **16a**


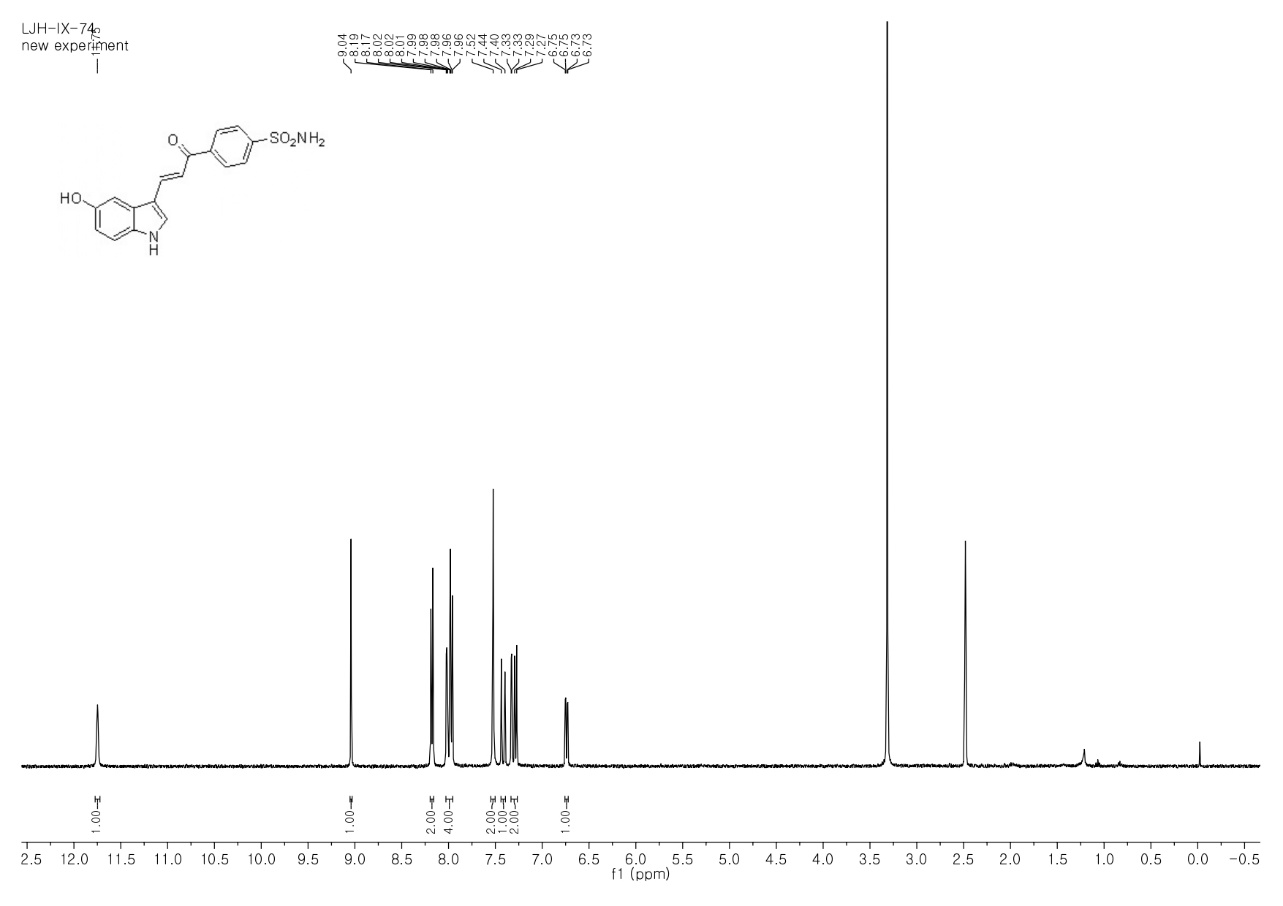


^1^H NMR spectrum of **16b**


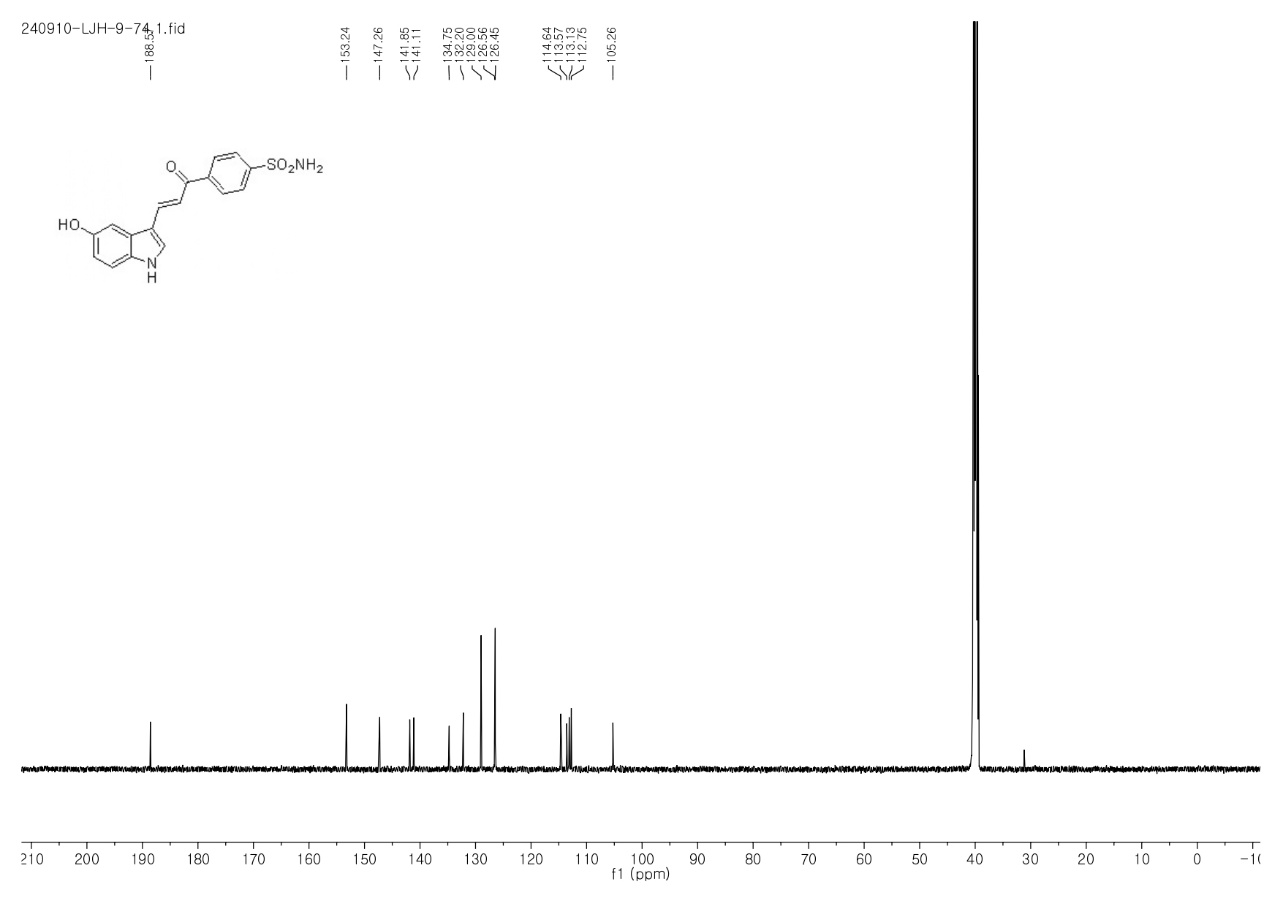


^13^C NMR spectrum of **16b**


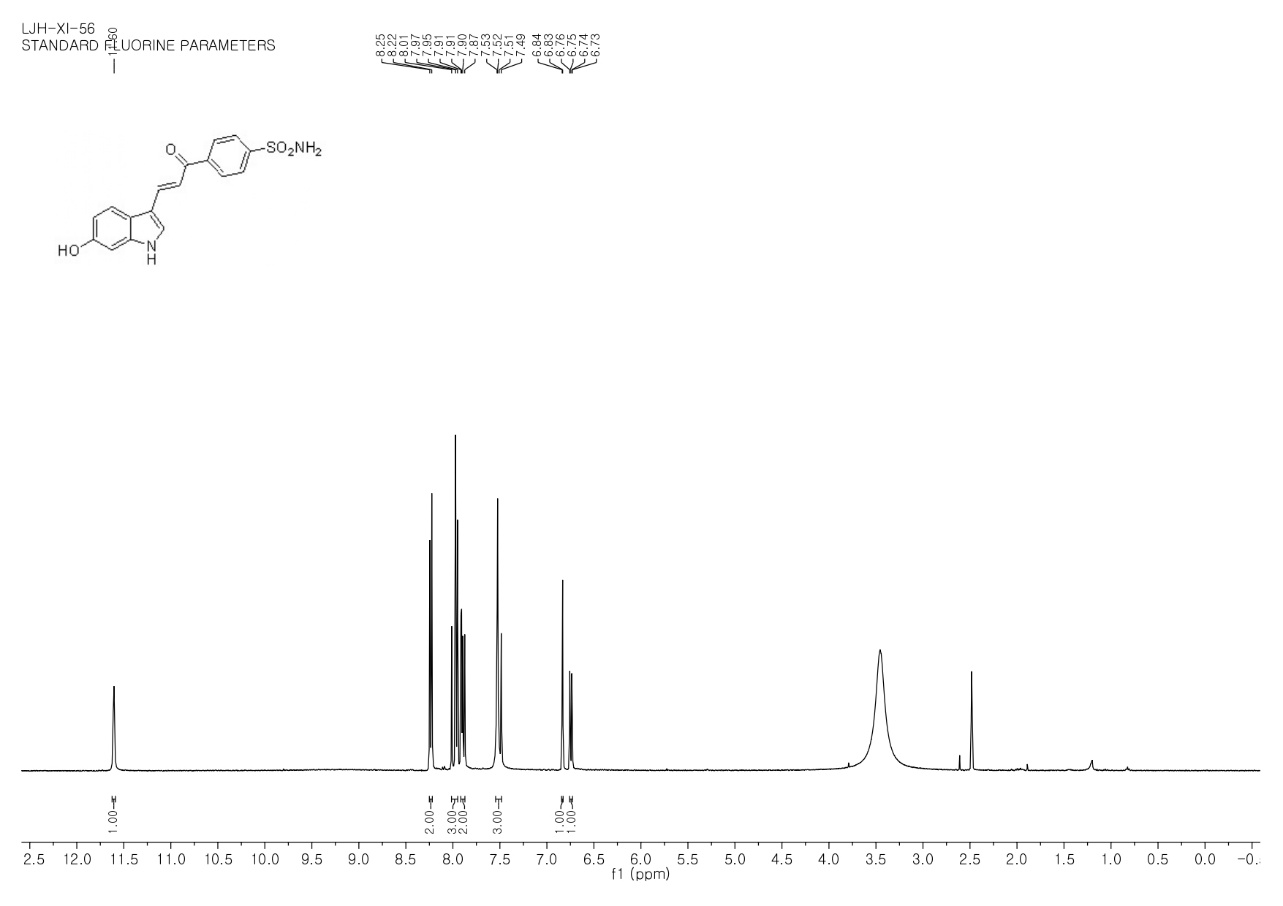


^1^H NMR spectrum of **16c**


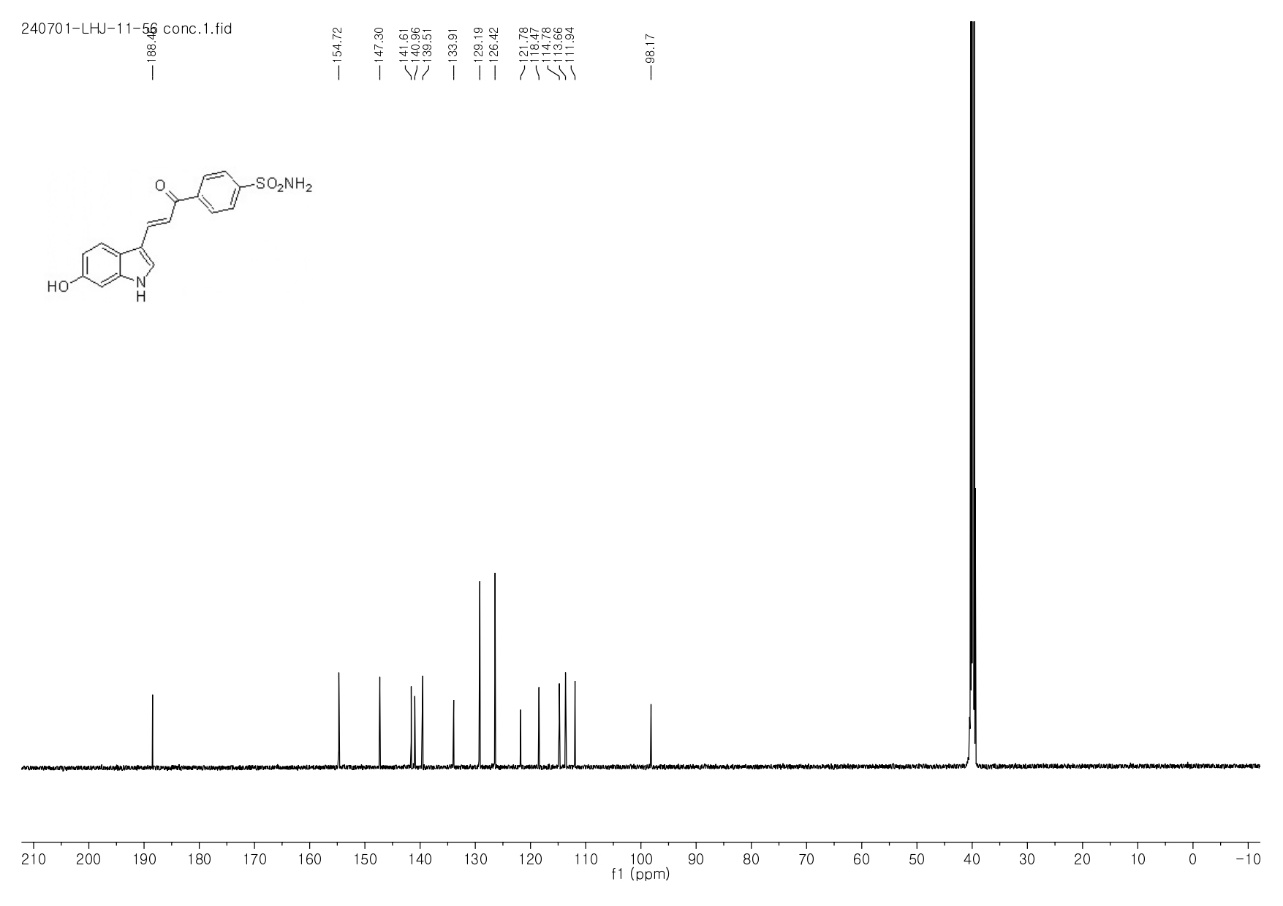


^13^C NMR spectrum of **16c**


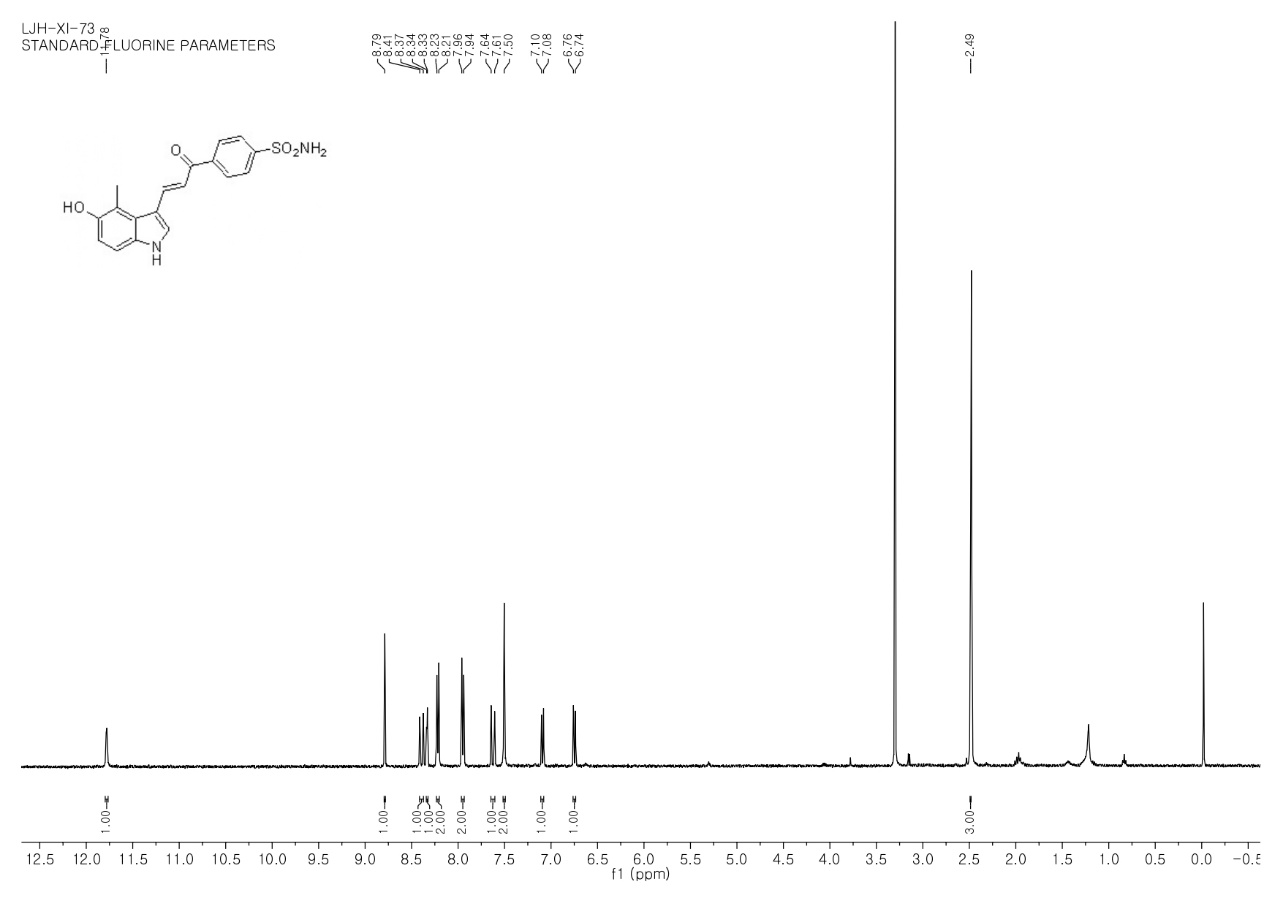


^1^H NMR spectrum of **16d**


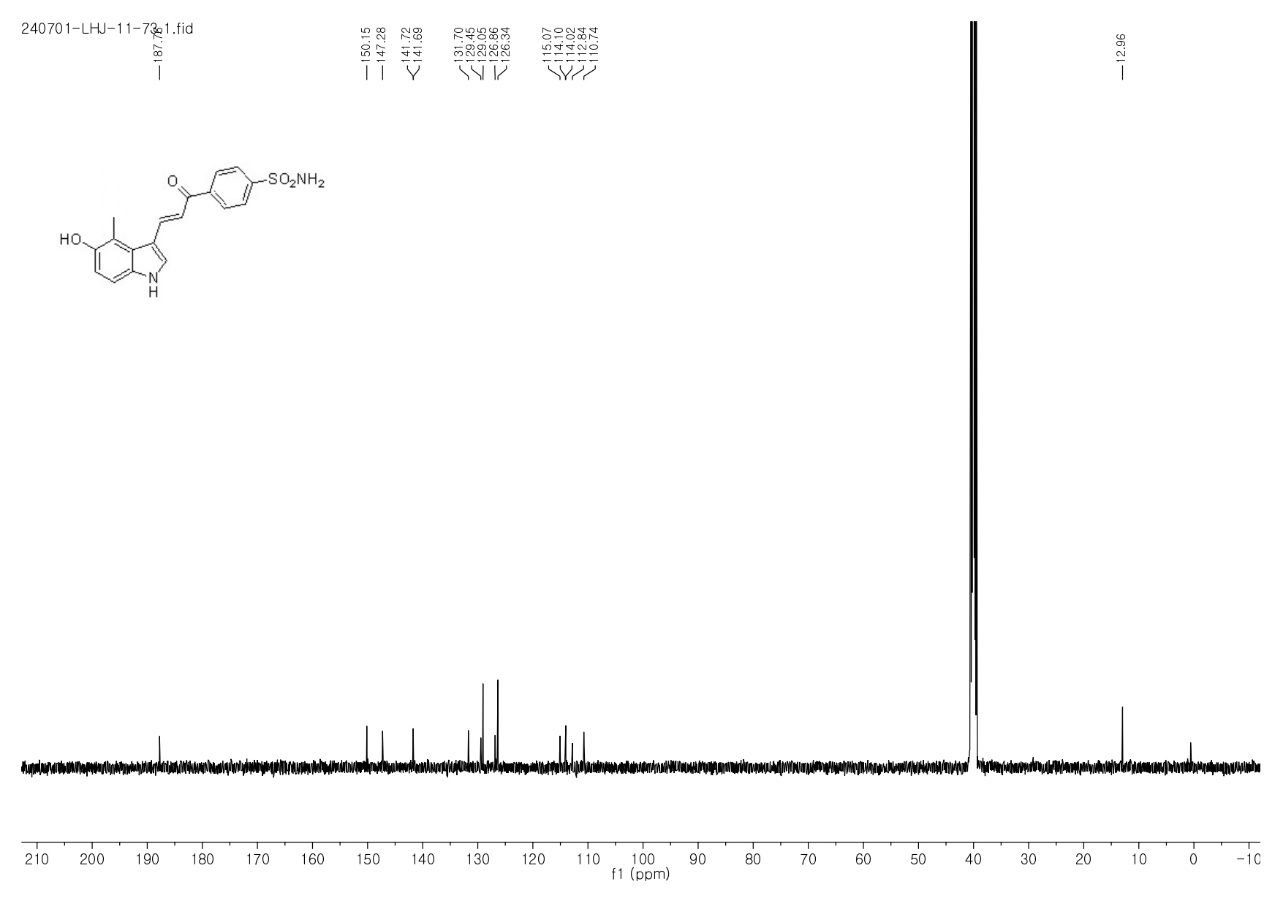


^13^C NMR spectrum of **16d**

**2. HPLC spectrum**


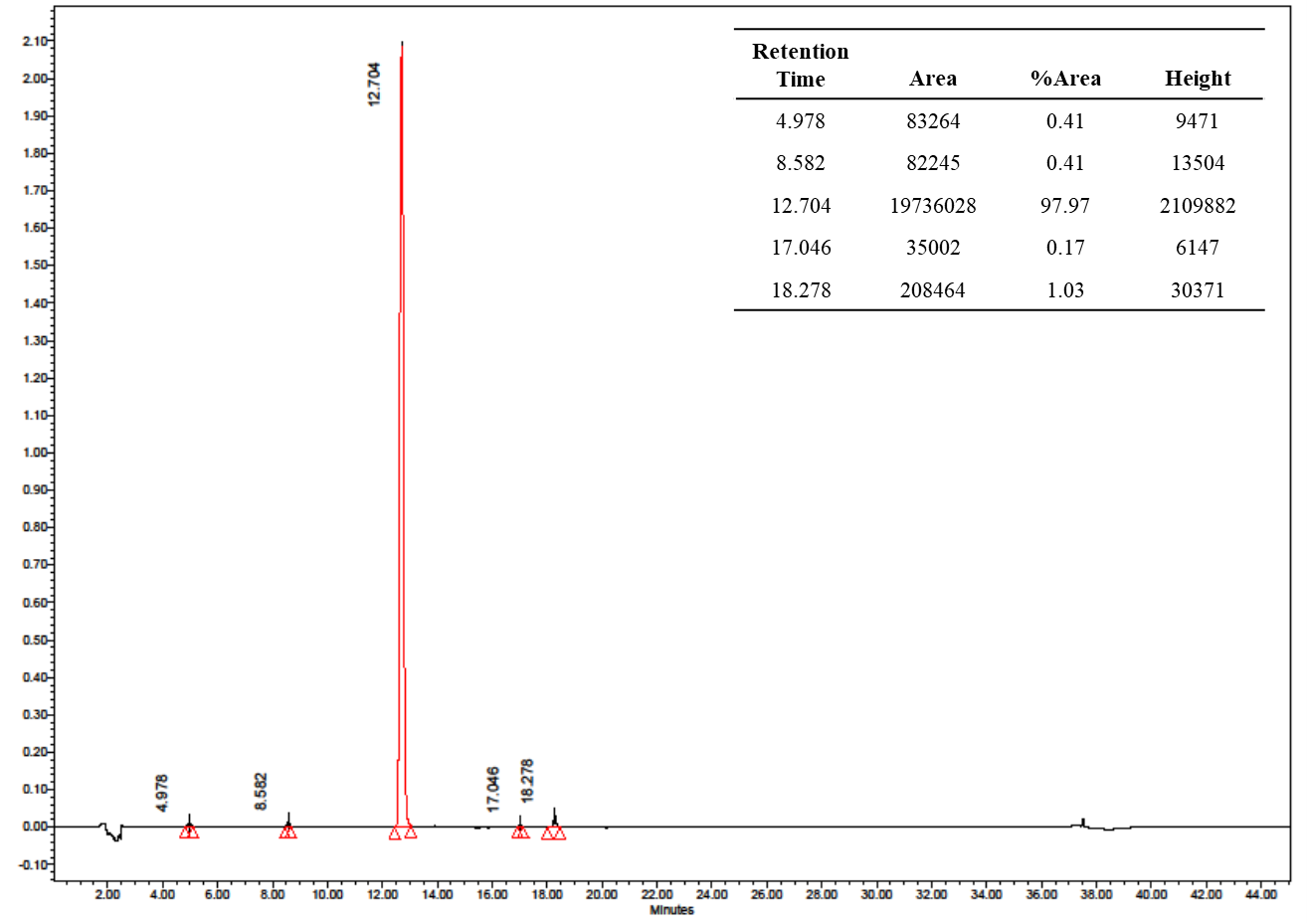


HPLC spectrum of **15a**


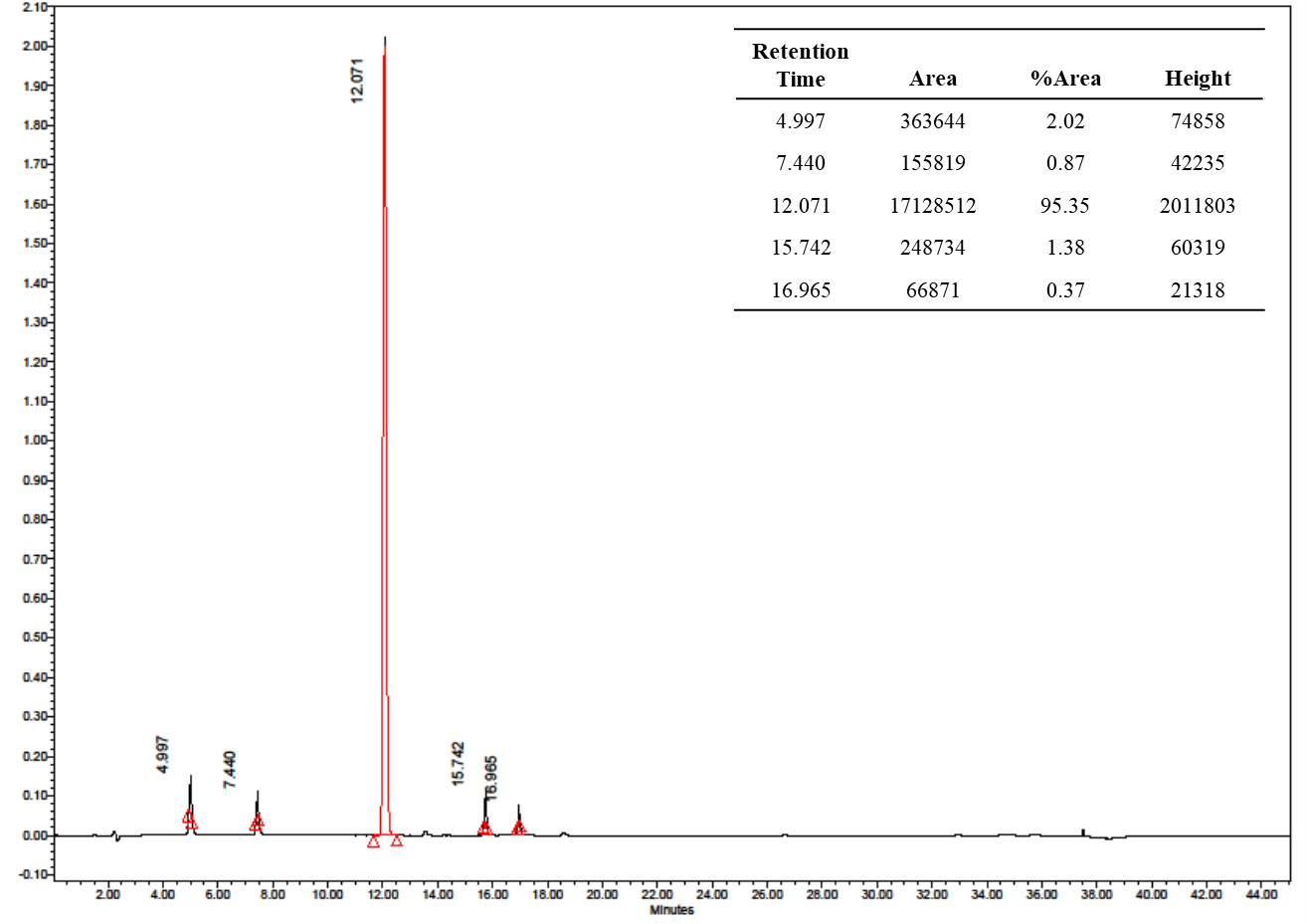


HPLC spectrum of **15b**


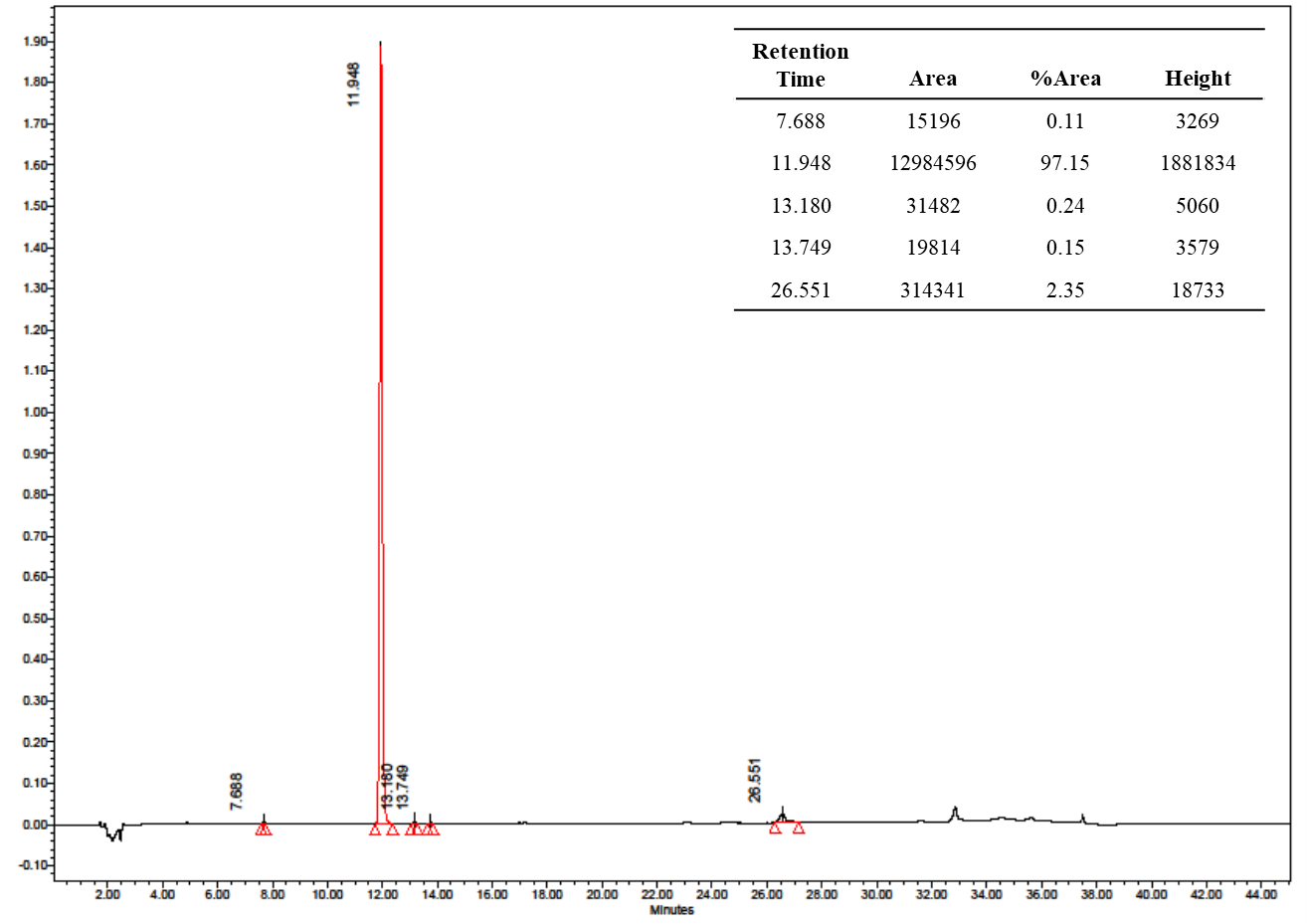


HPLC spectrum of **15c**


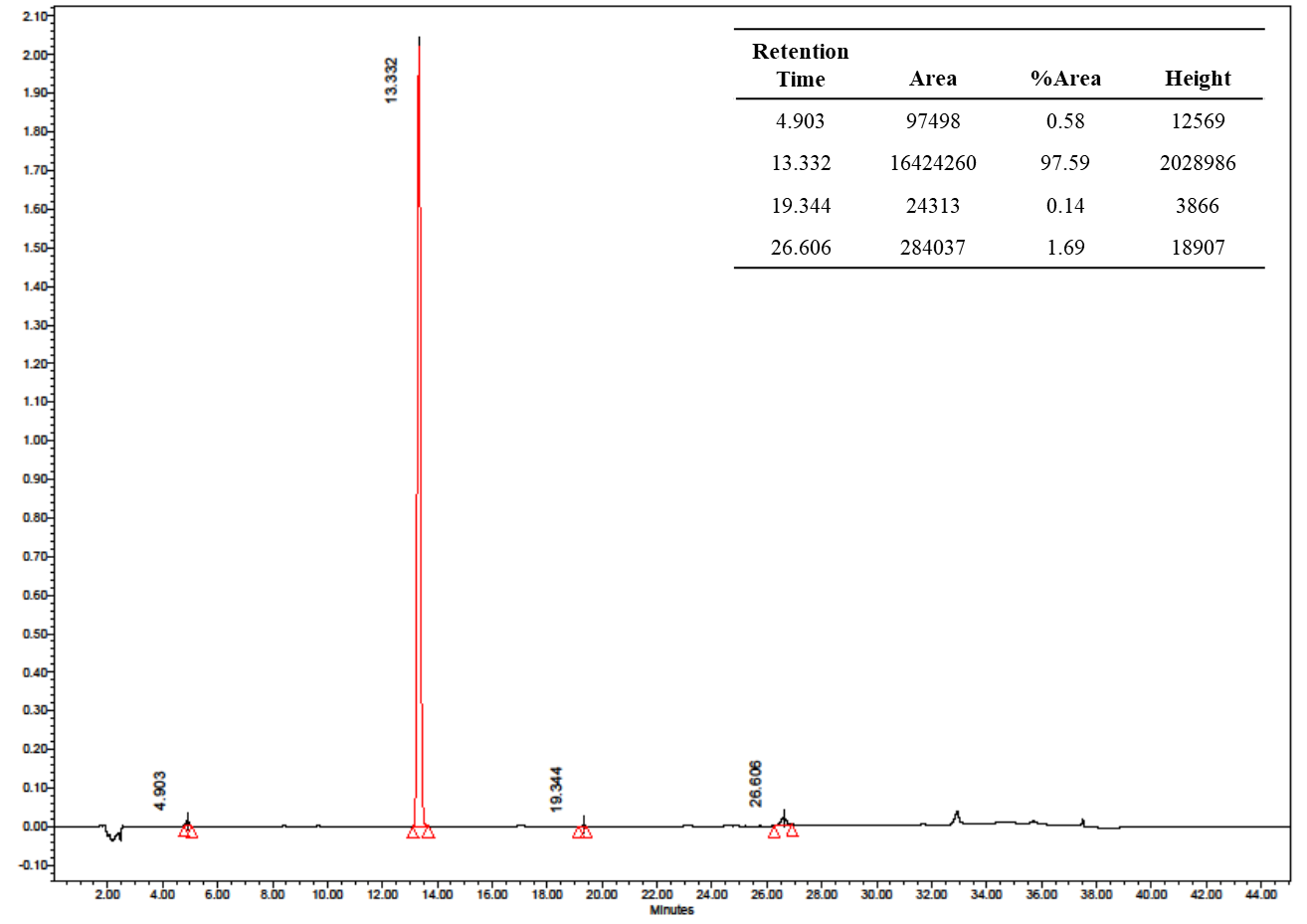


HPLC spectrum of **15d**


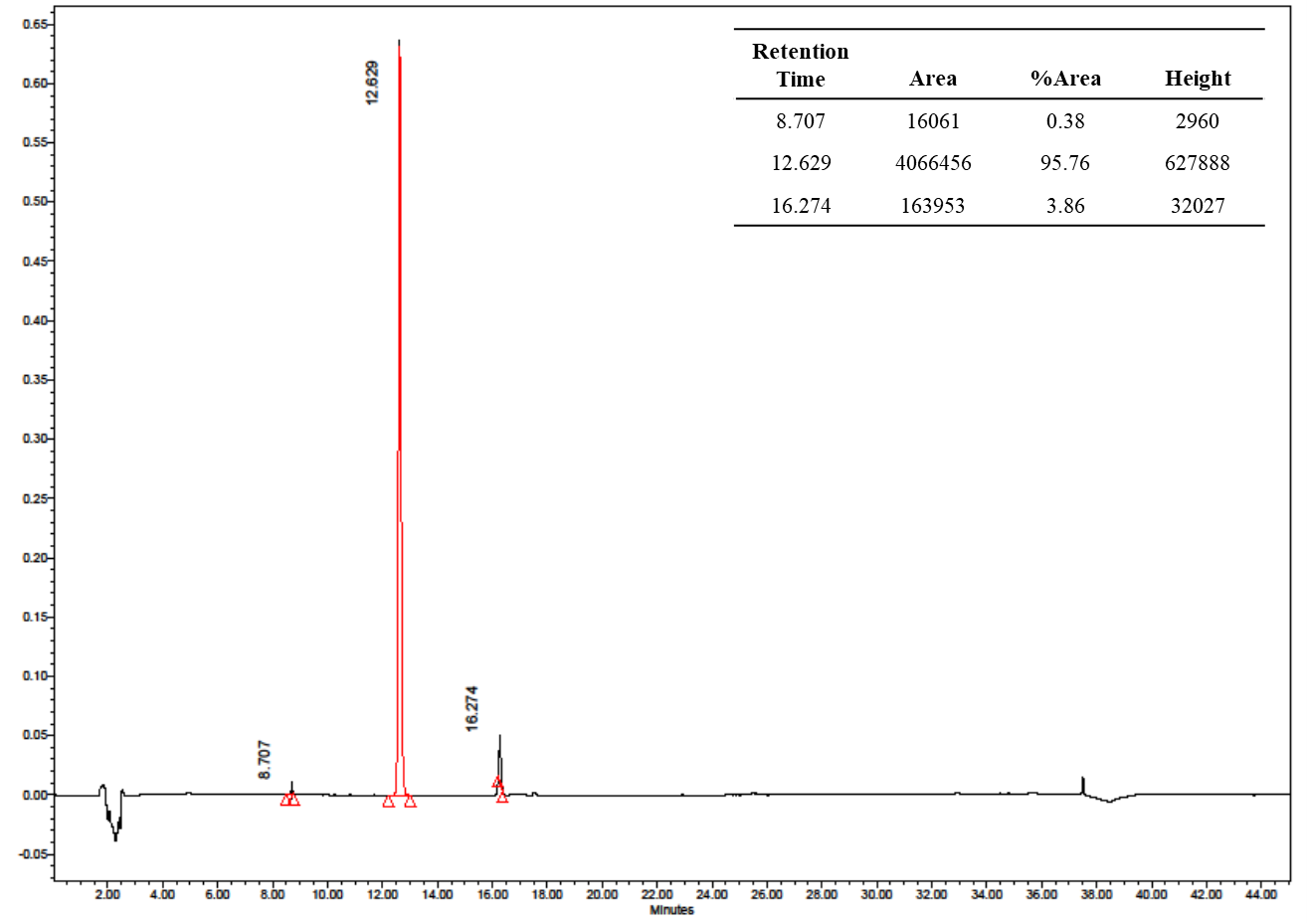


HPLC spectrum of **15e**


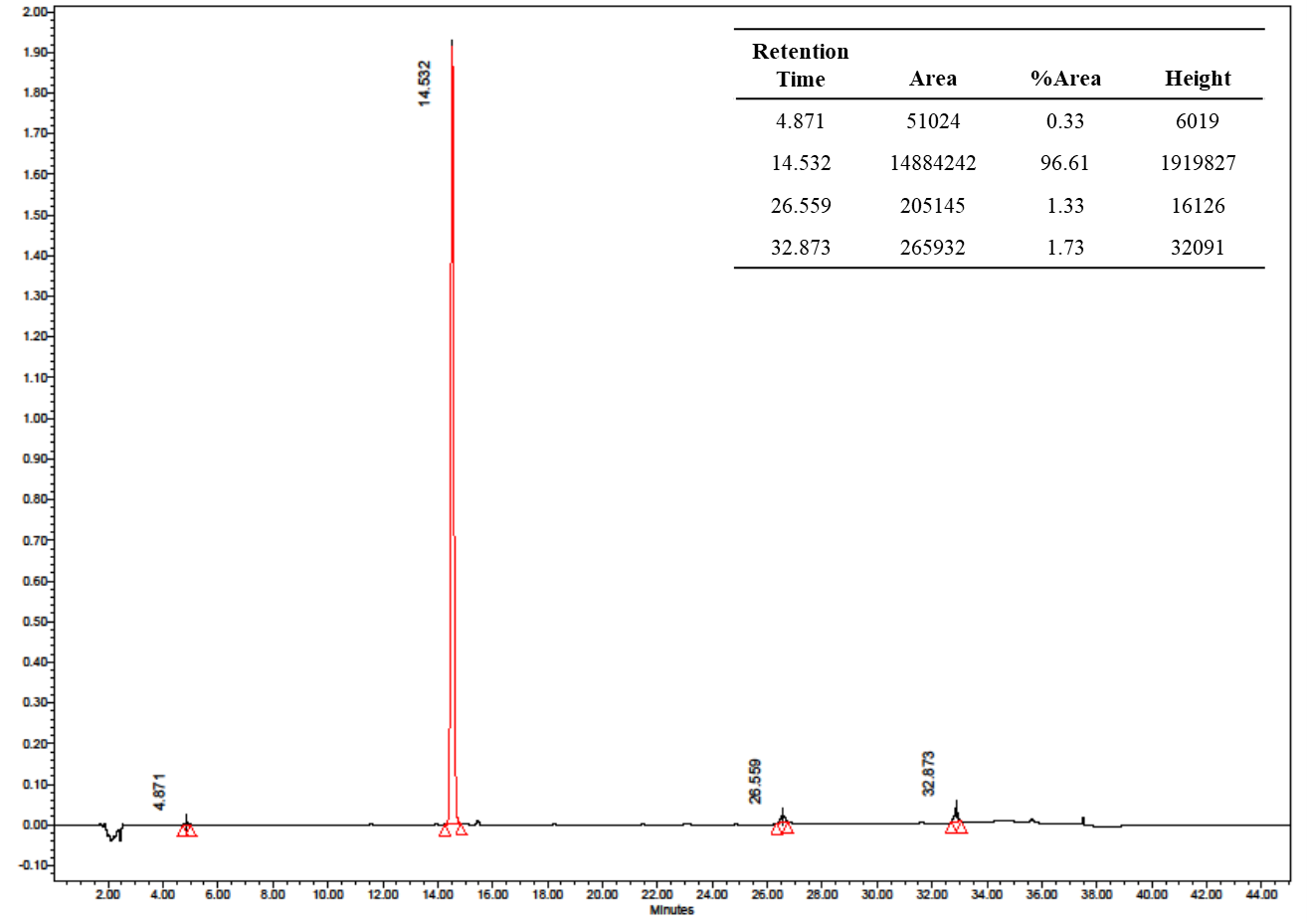


HPLC spectrum of **15f**


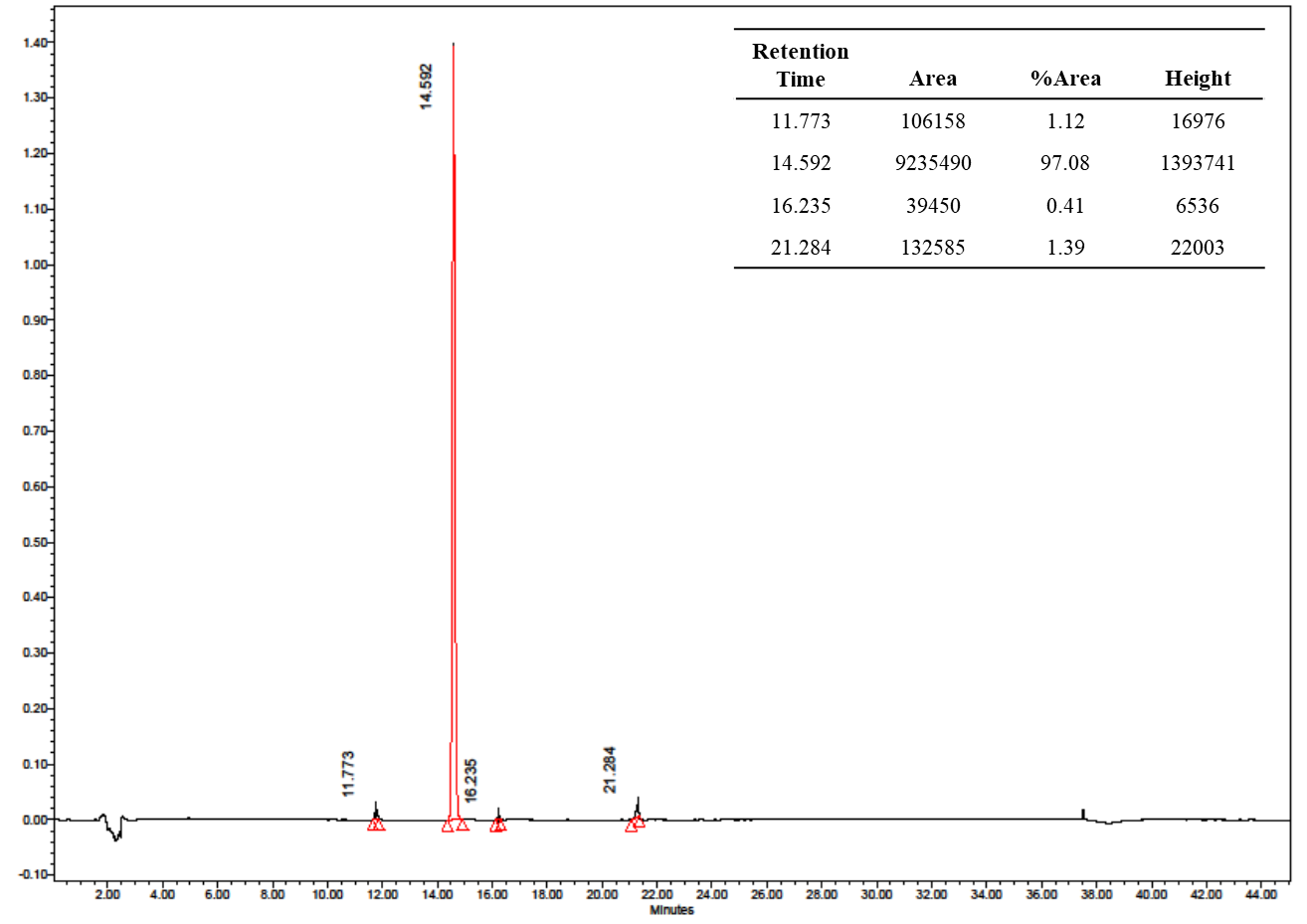


HPLC spectrum of **15g**


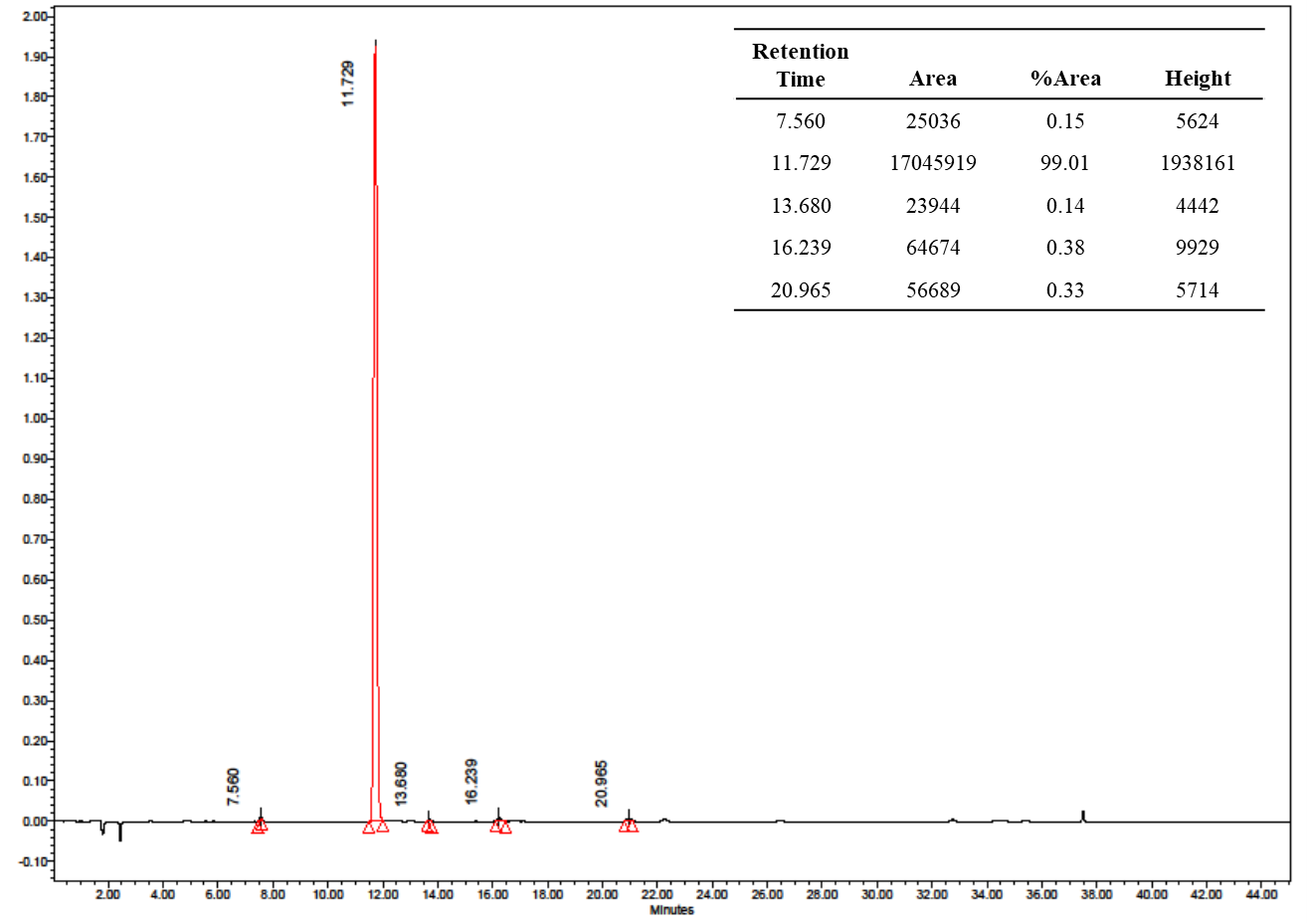


HPLC spectrum of **15h**


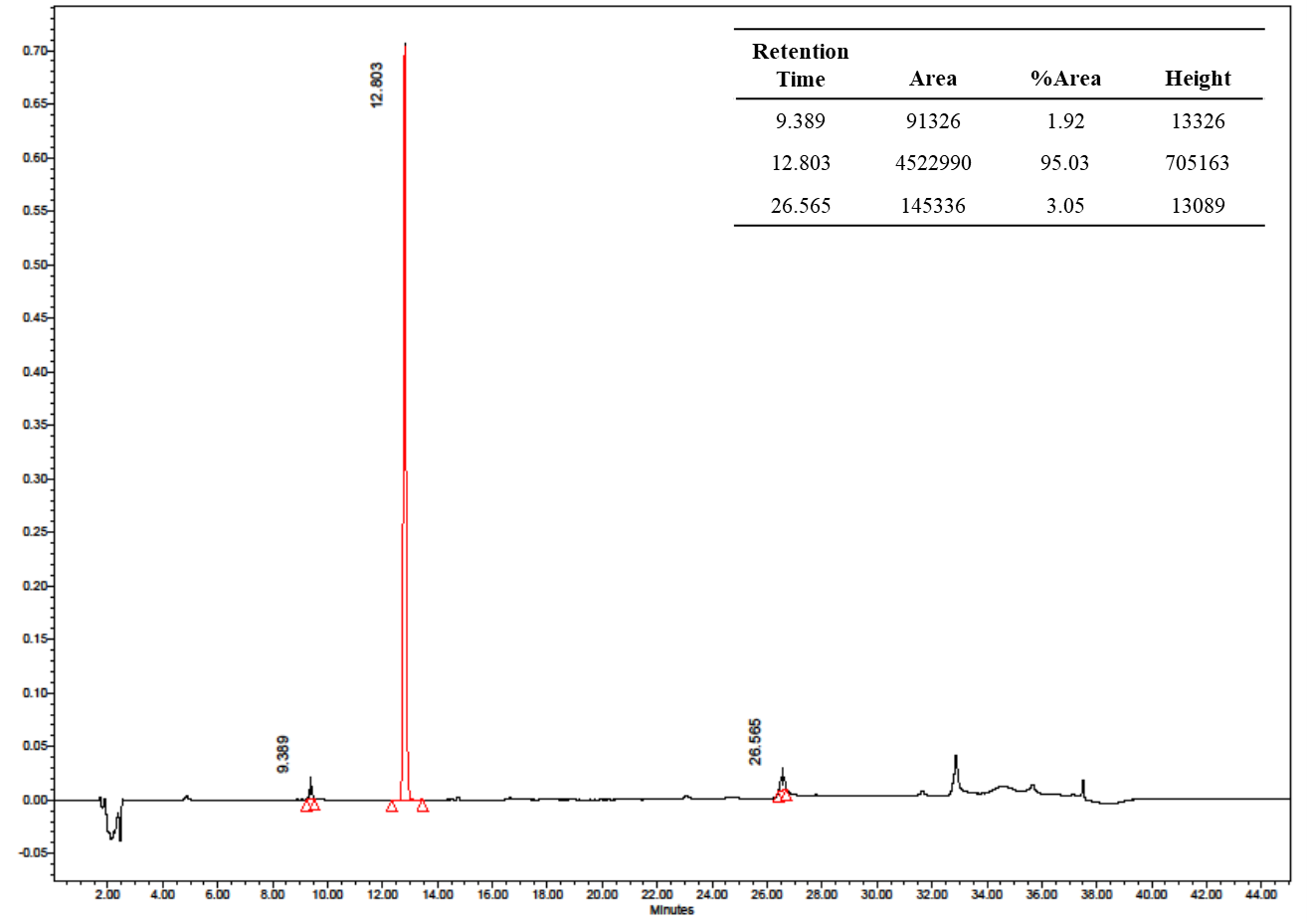


HPLC spectrum of **15i**


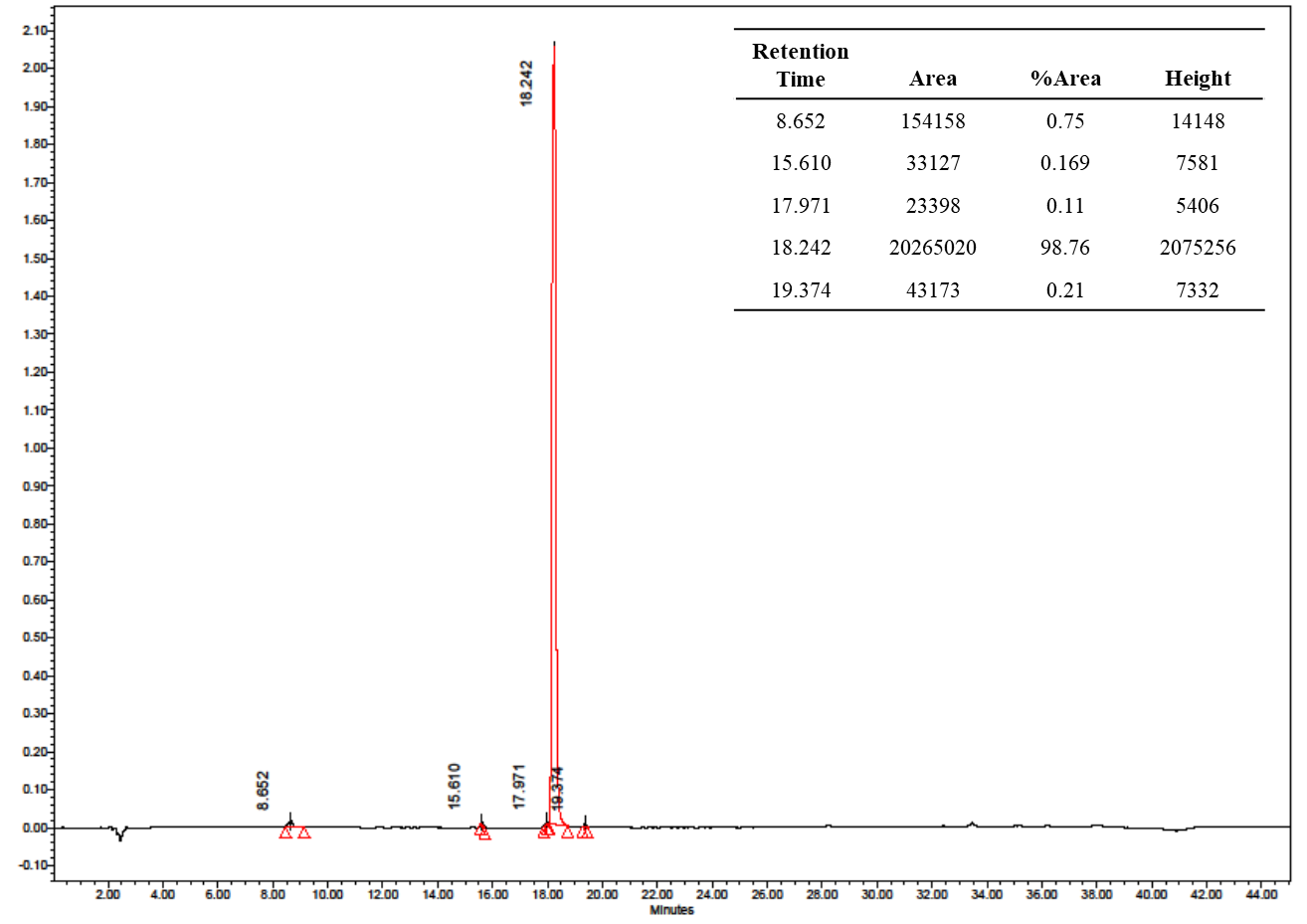


HPLC spectrum of **15j**


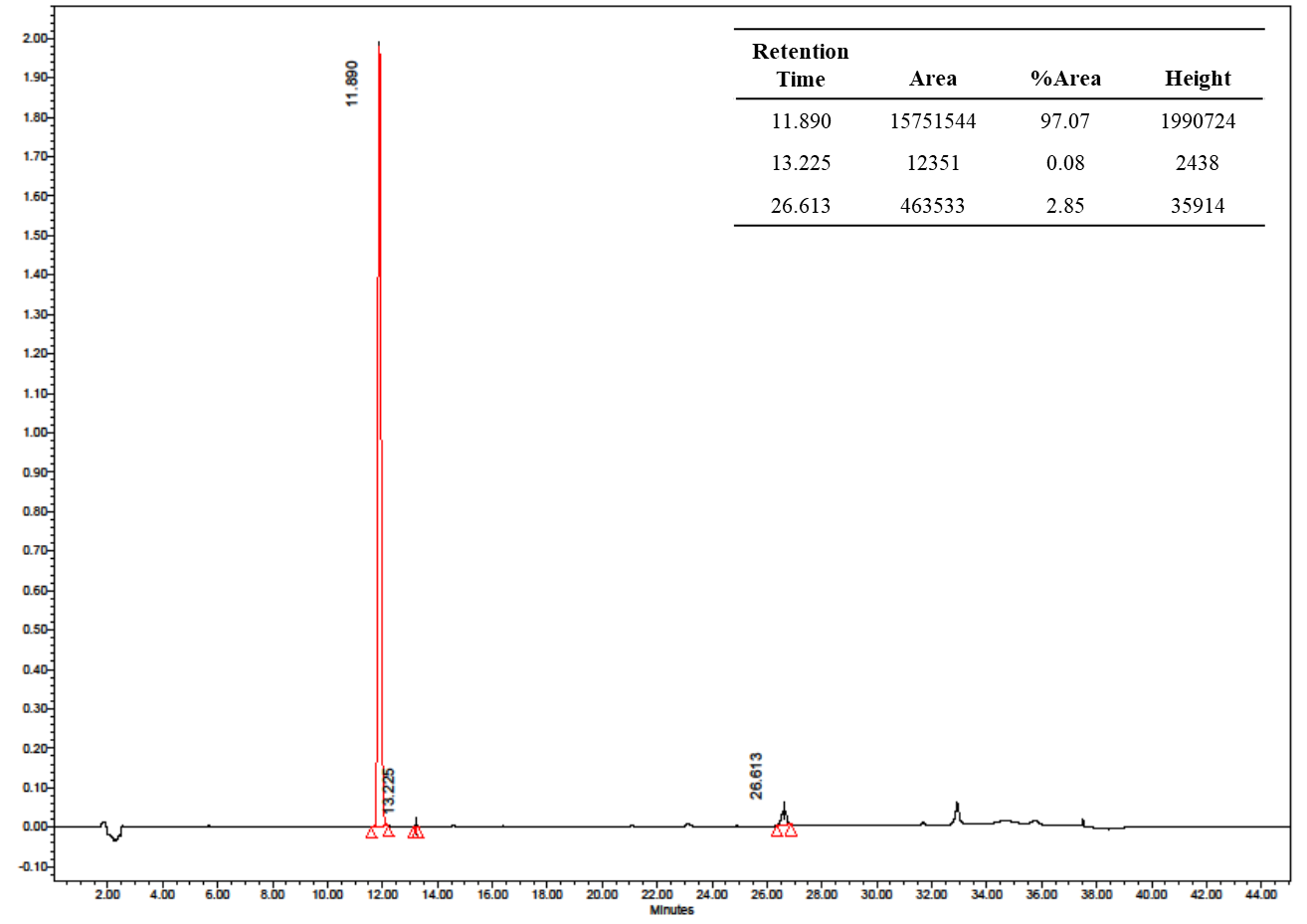


HPLC spectrum of **15k**


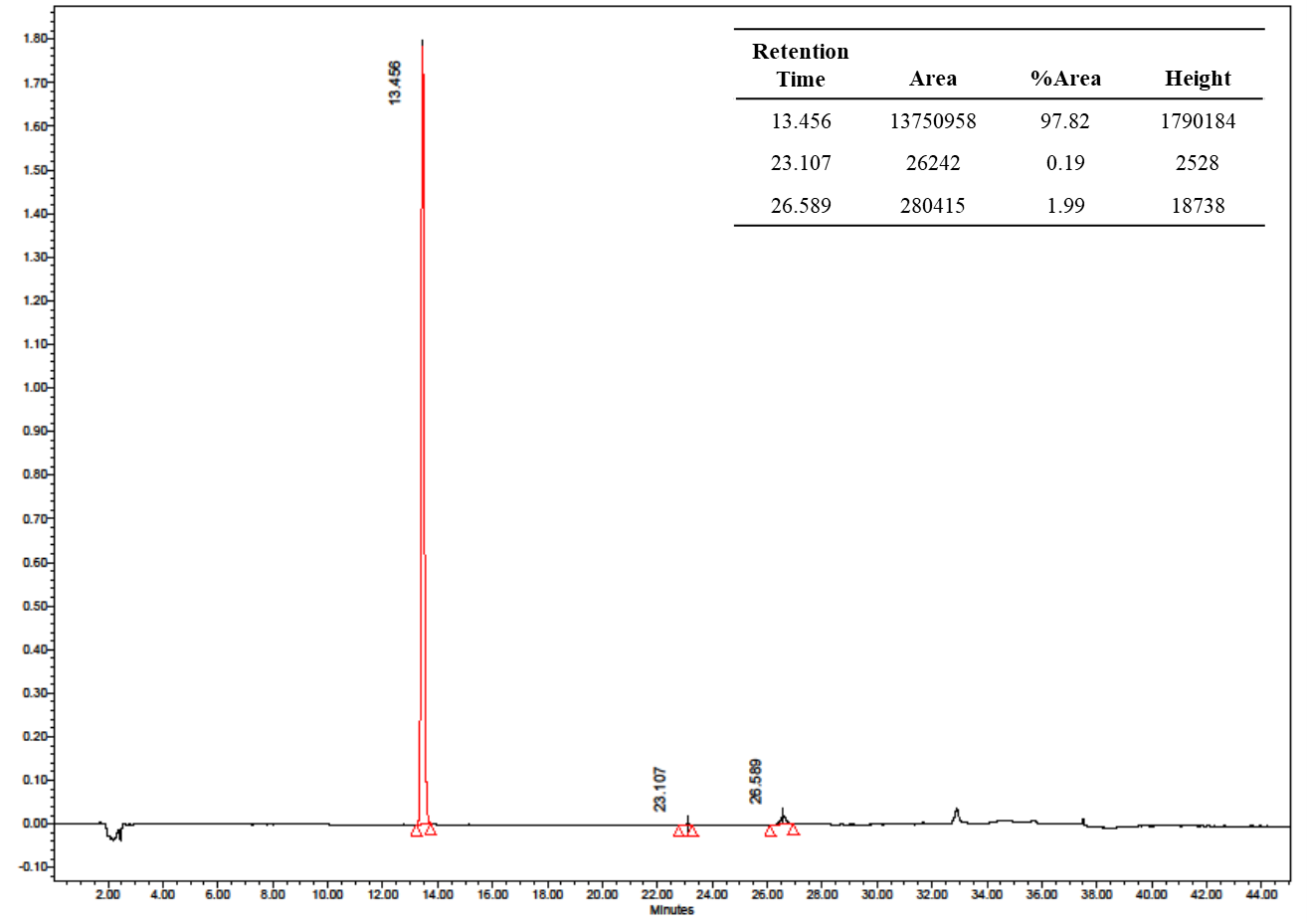


HPLC spectrum of **15l**


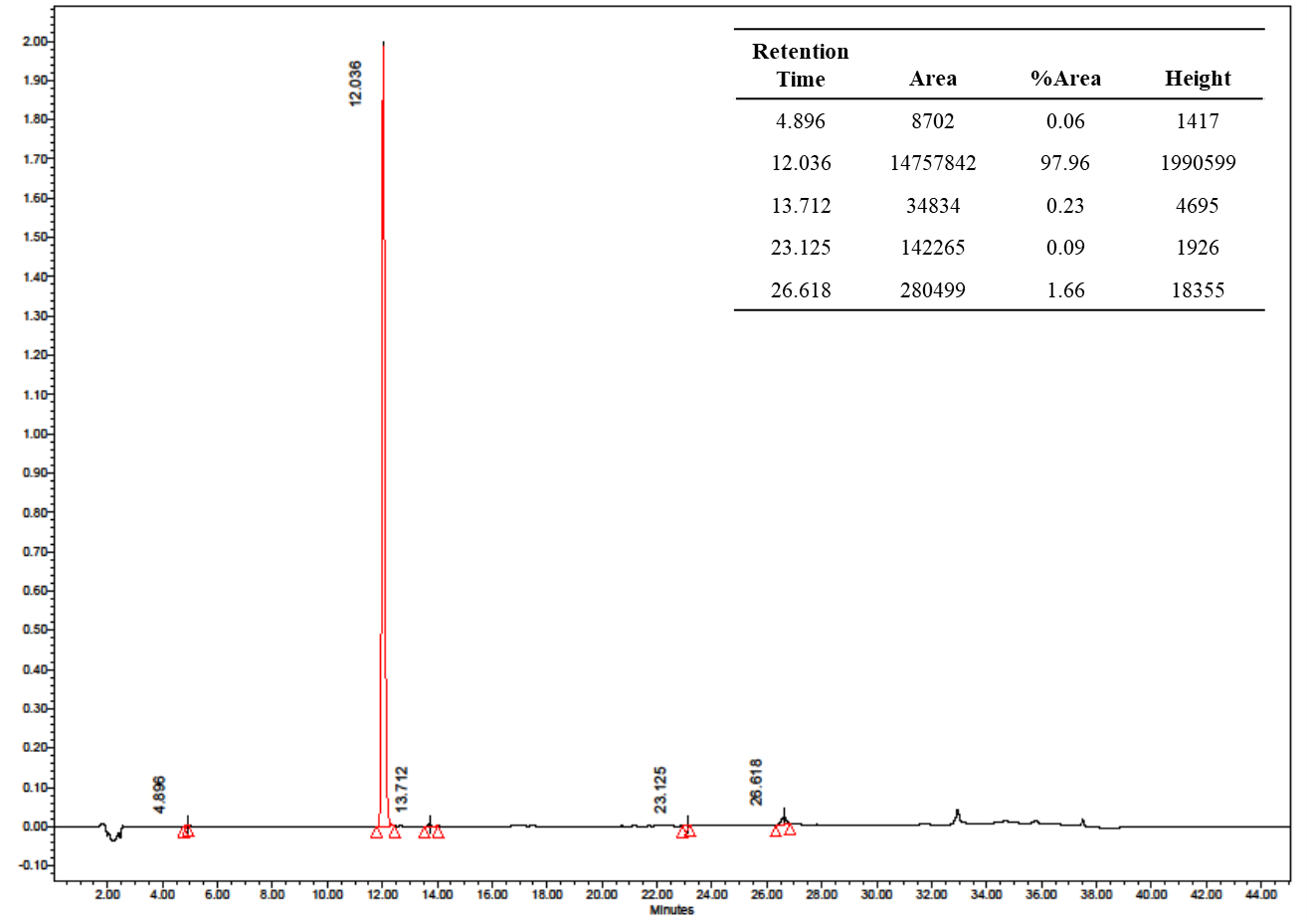


HPLC spectrum of **15m**


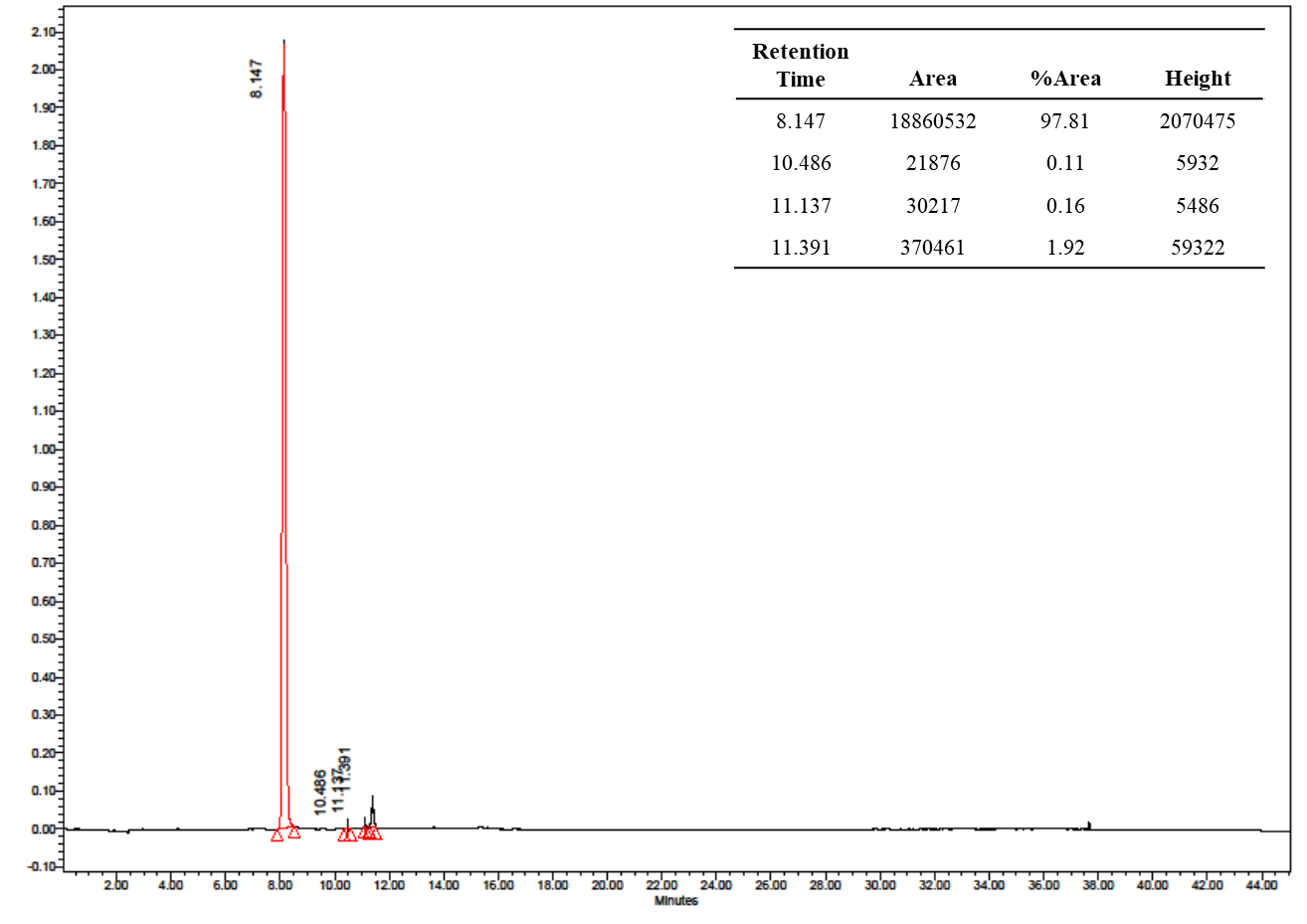


HPLC spectrum of **16a**


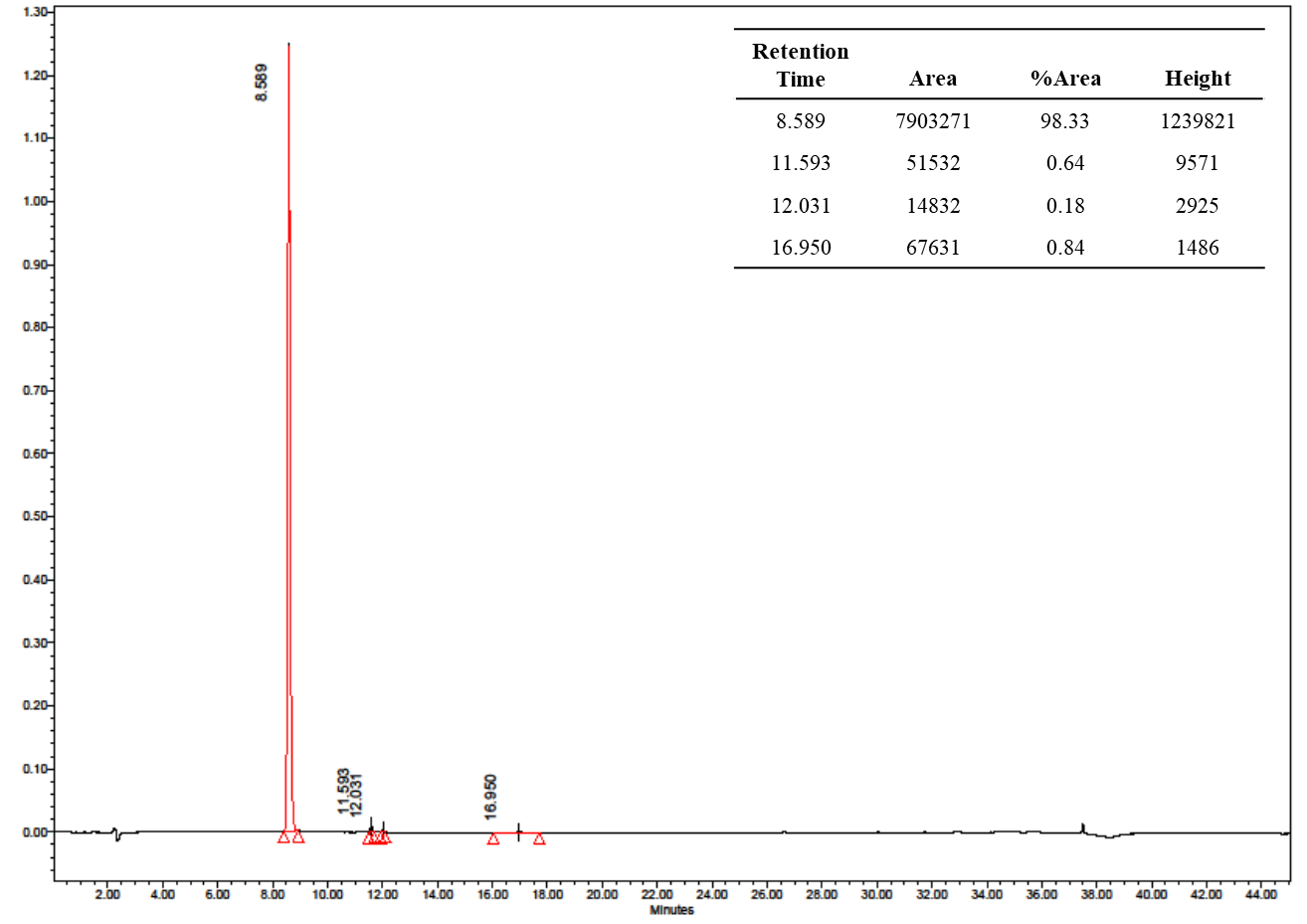


HPLC spectrum of **16b**


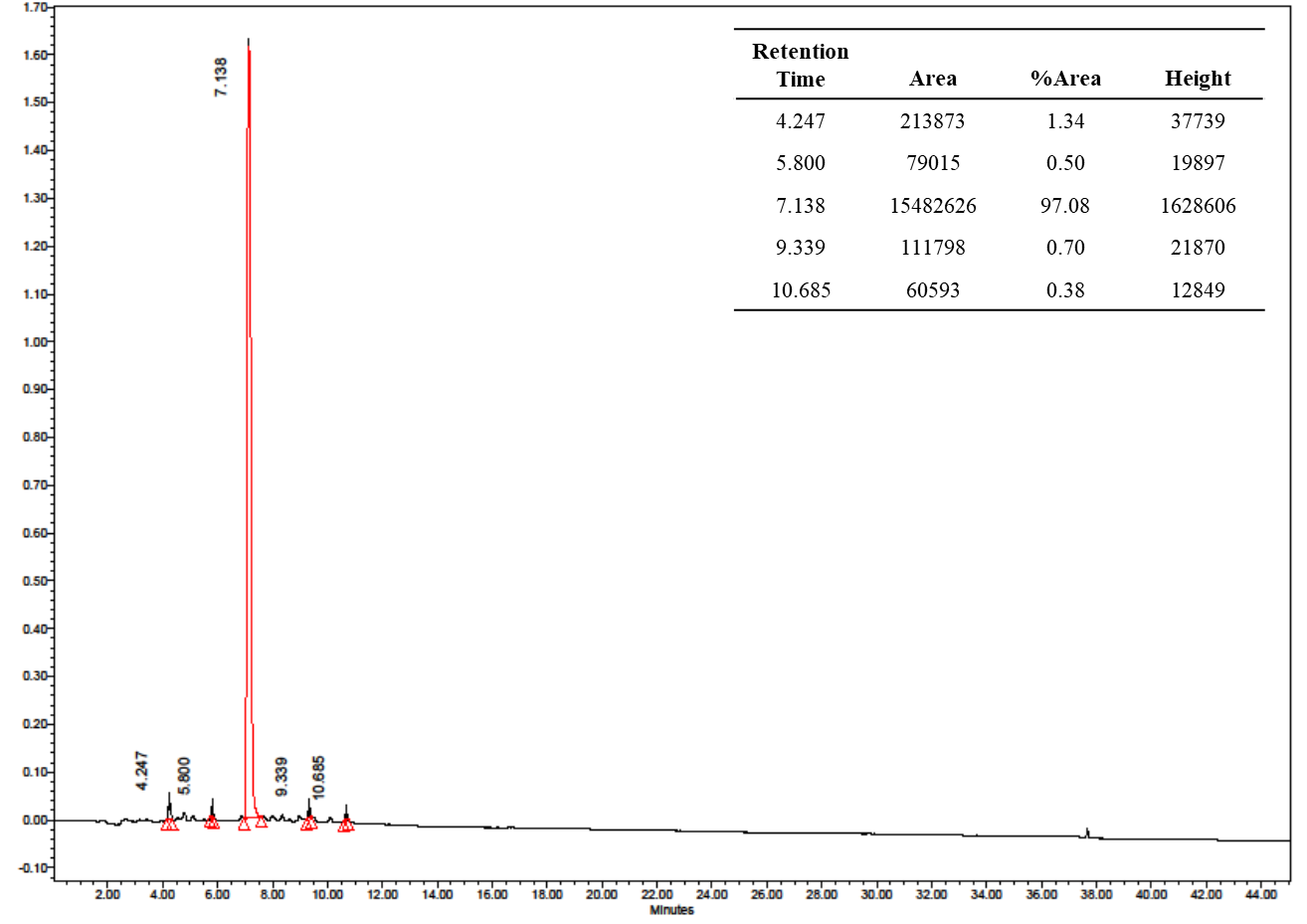


HPLC spectrum of **16c**


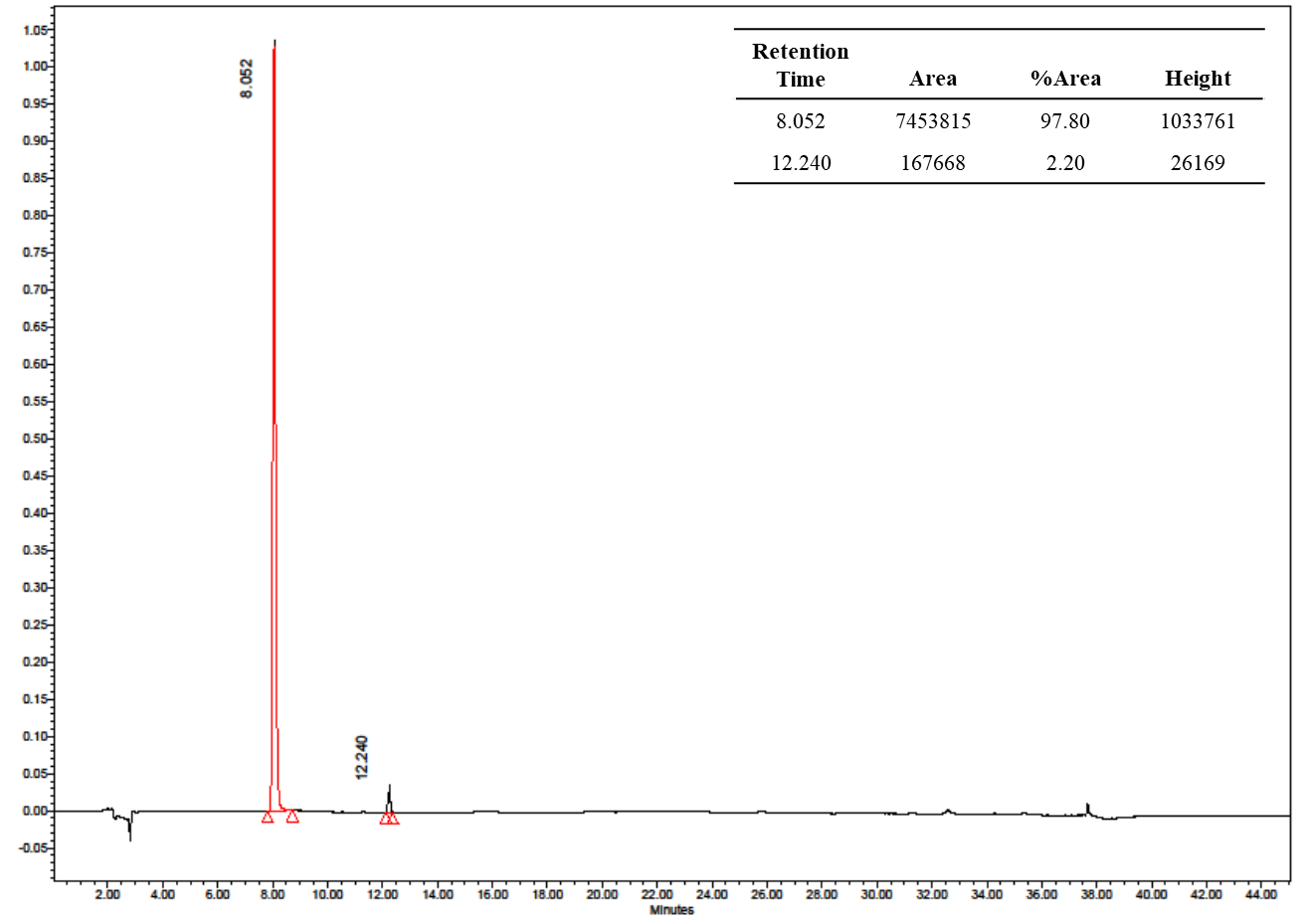


HPLC spectrum of **16d**

**3. HRMS spectrum**


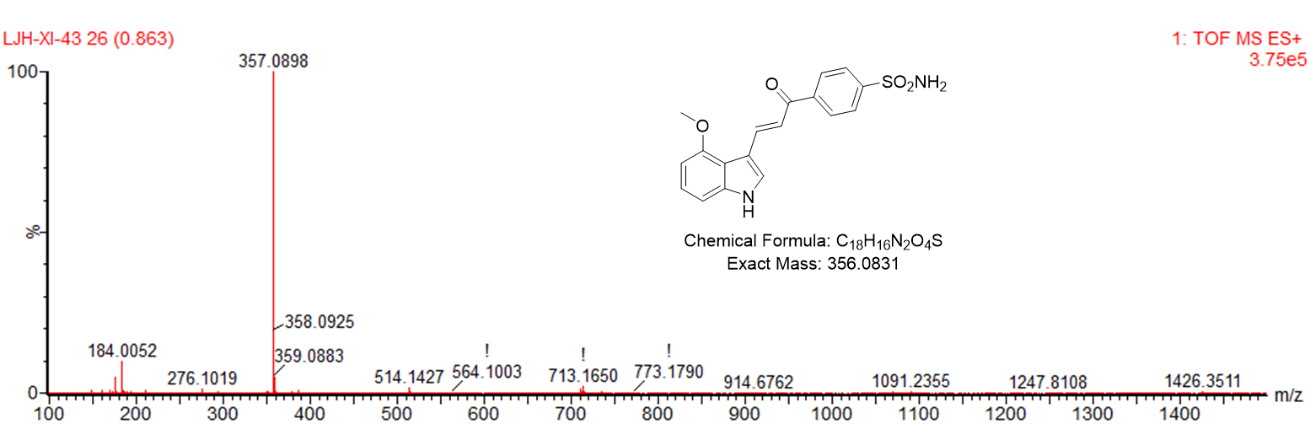


HRMS spectrum of **15a**


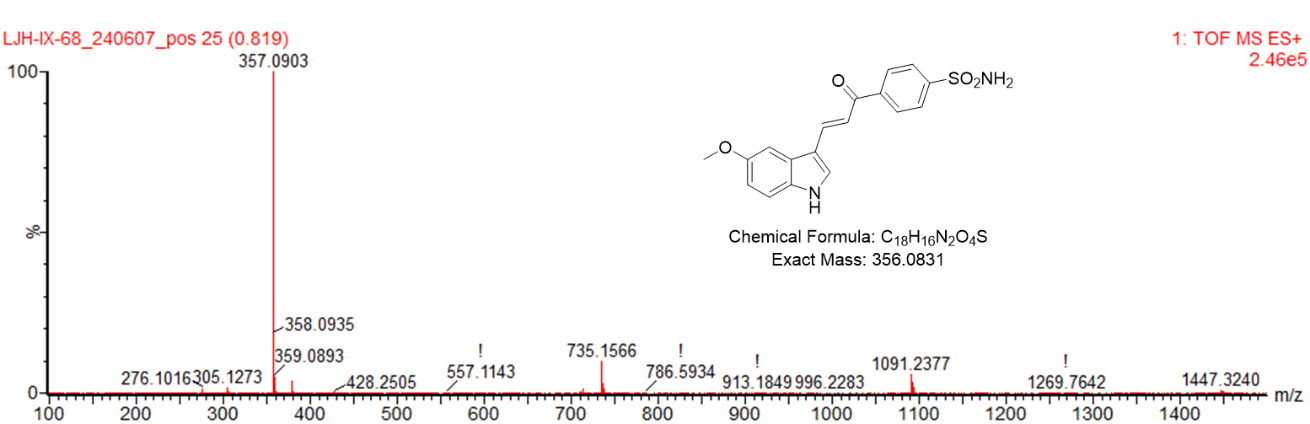


HRMS spectrum of **15b**


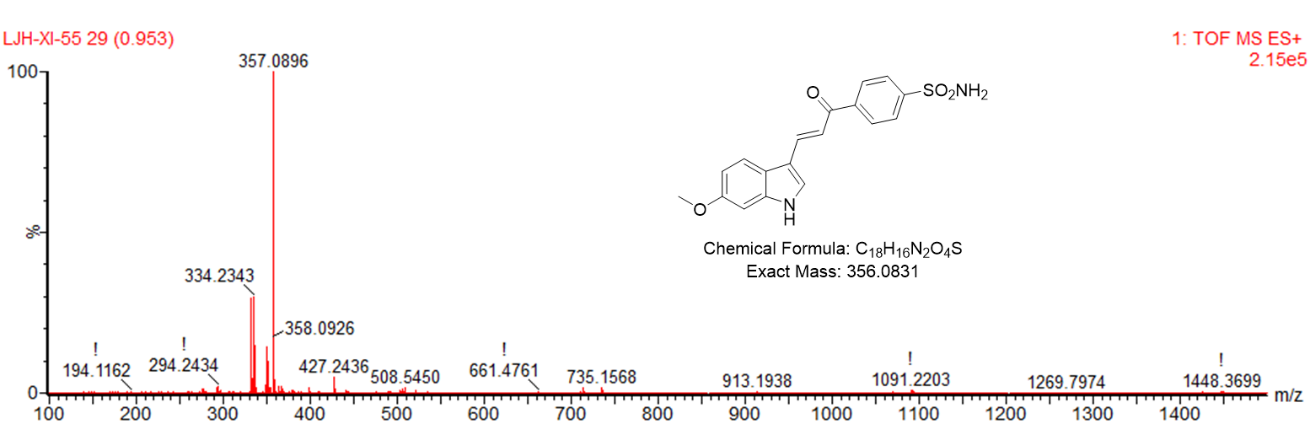


HRMS spectrum of **15c**


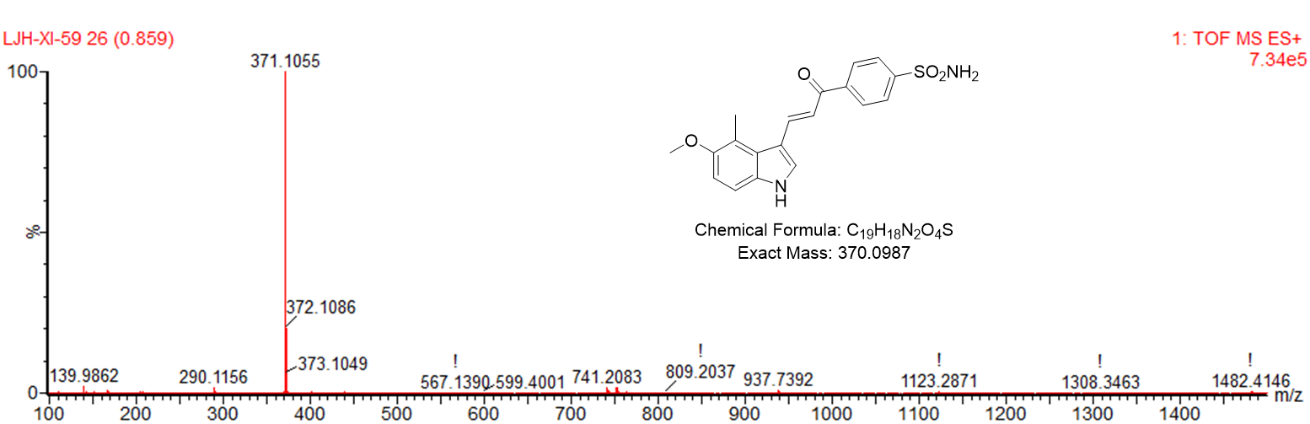


HRMS spectrum of **15d**


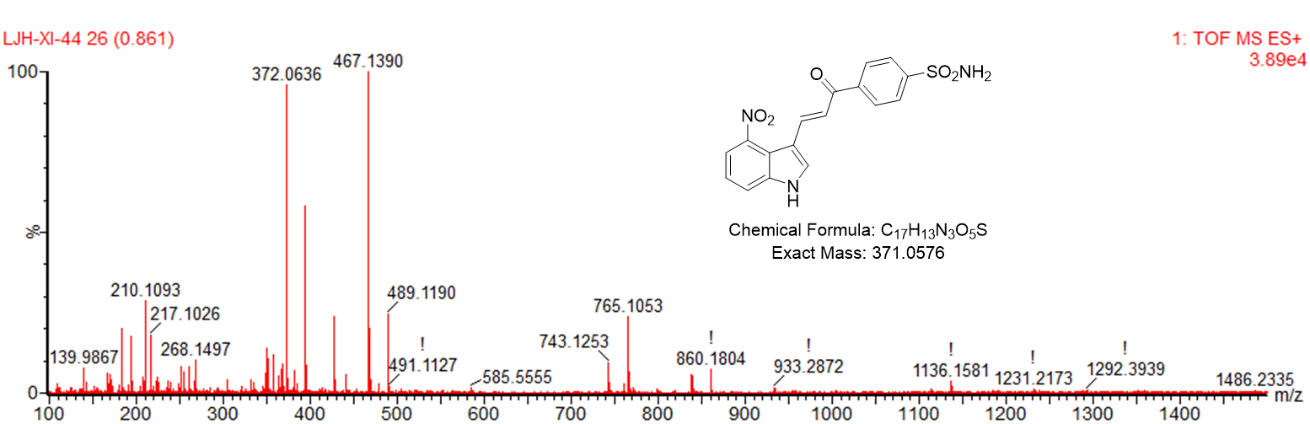


HRMS spectrum of **15e**


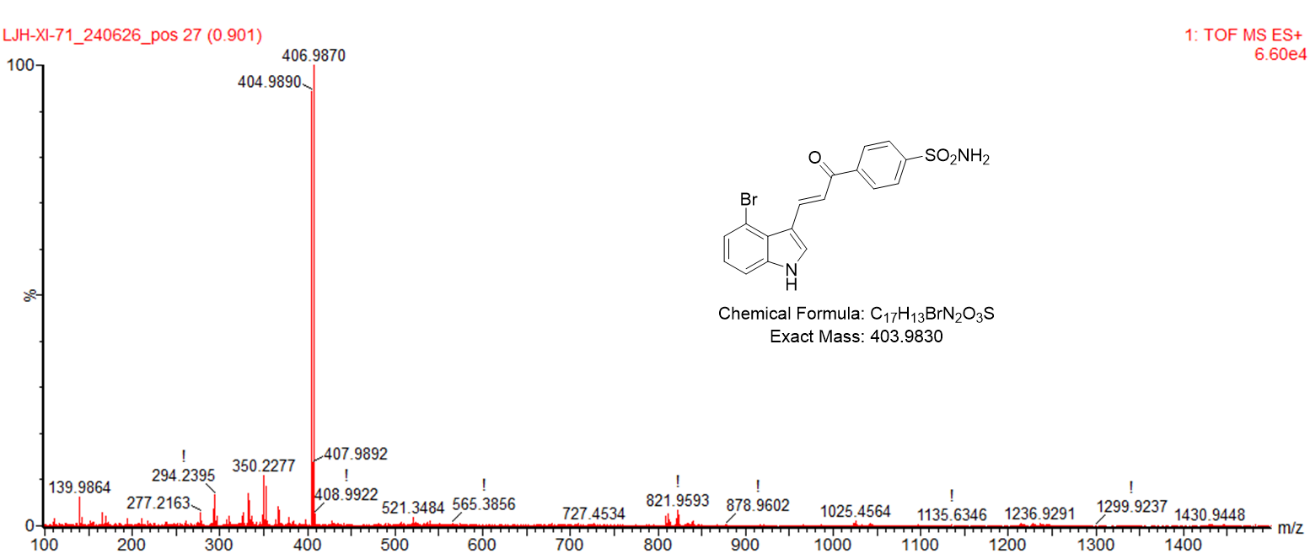


HRMS spectrum of **15f**


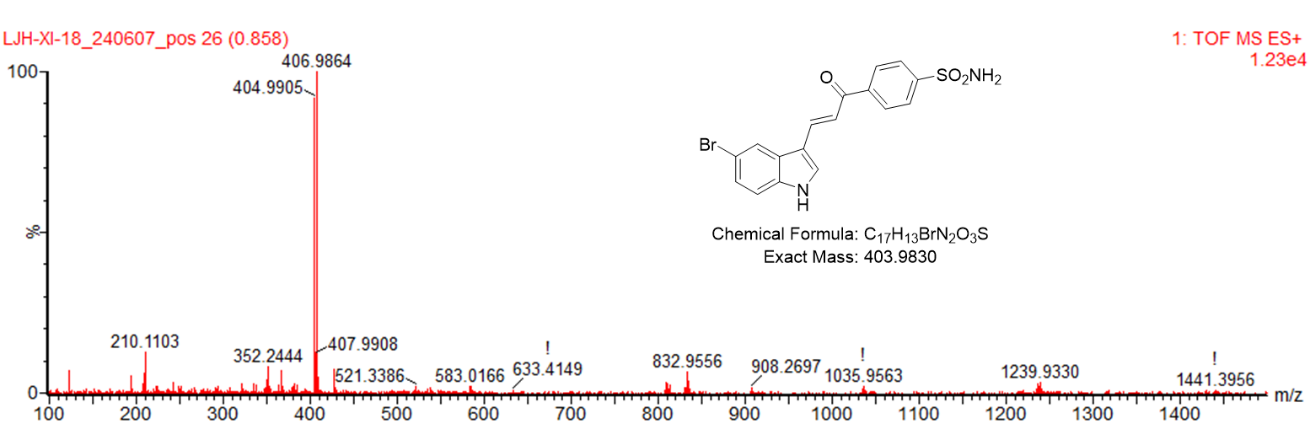


HRMS spectrum of **15g**


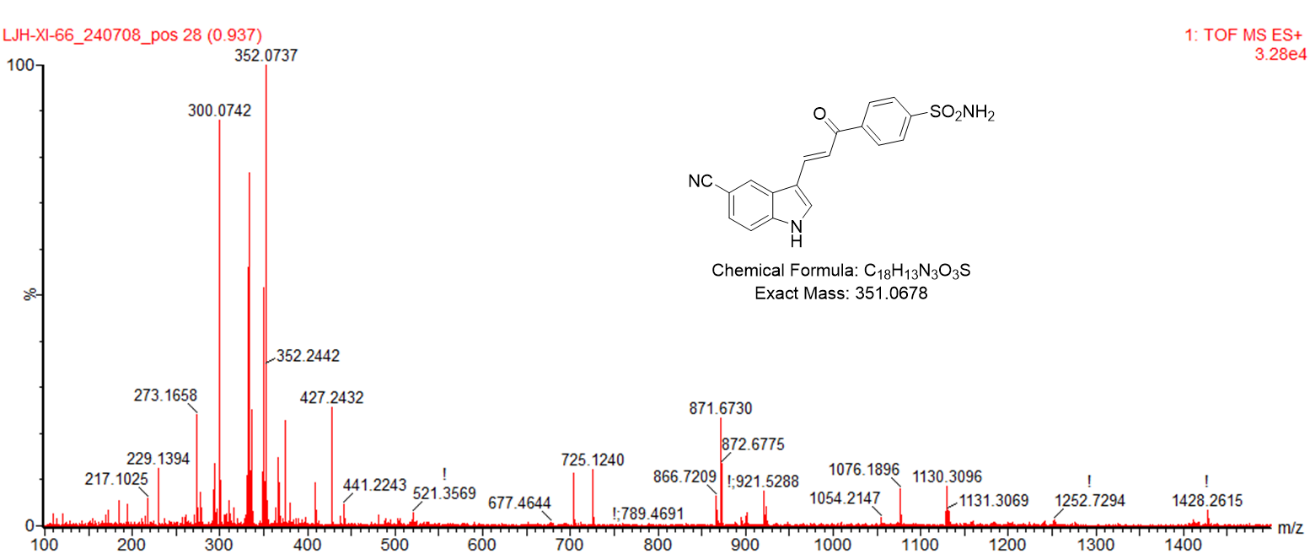


HRMS spectrum of **15h**


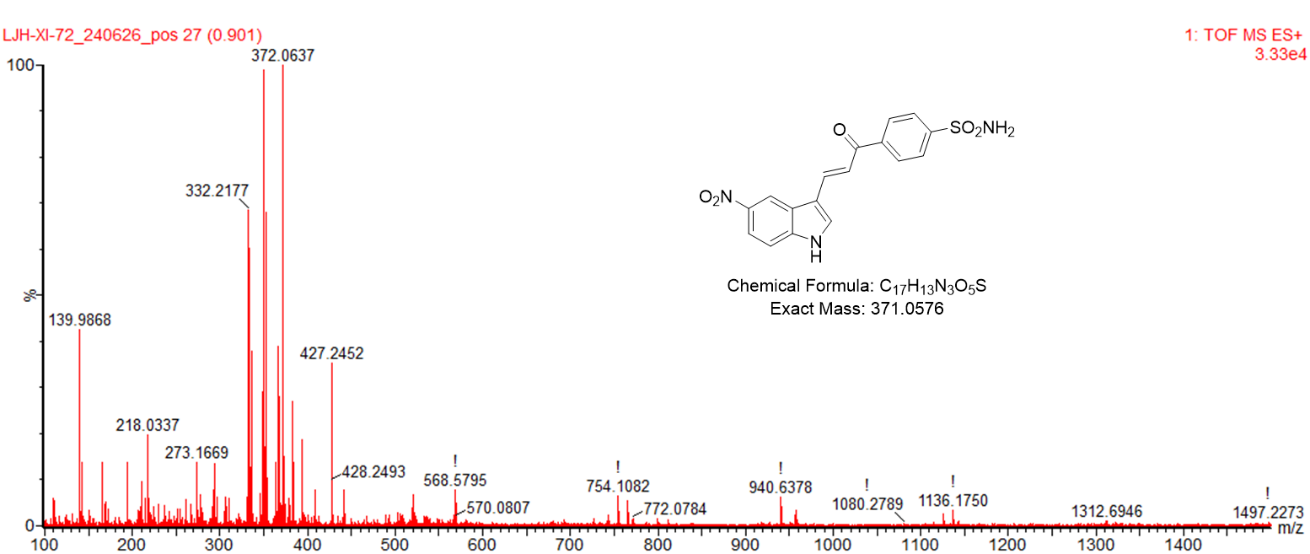


HRMS spectrum of **15i**


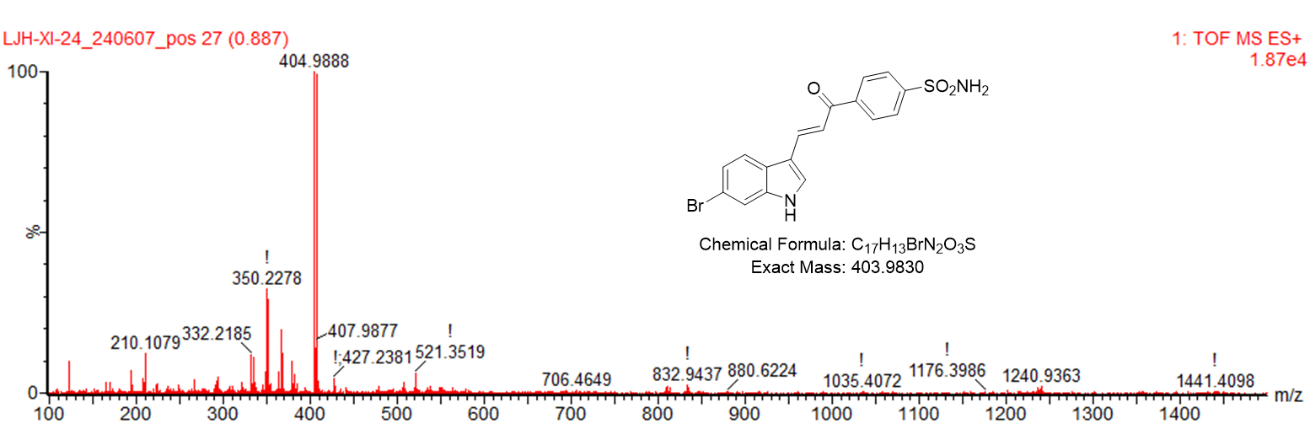


HRMS spectrum of **15j**


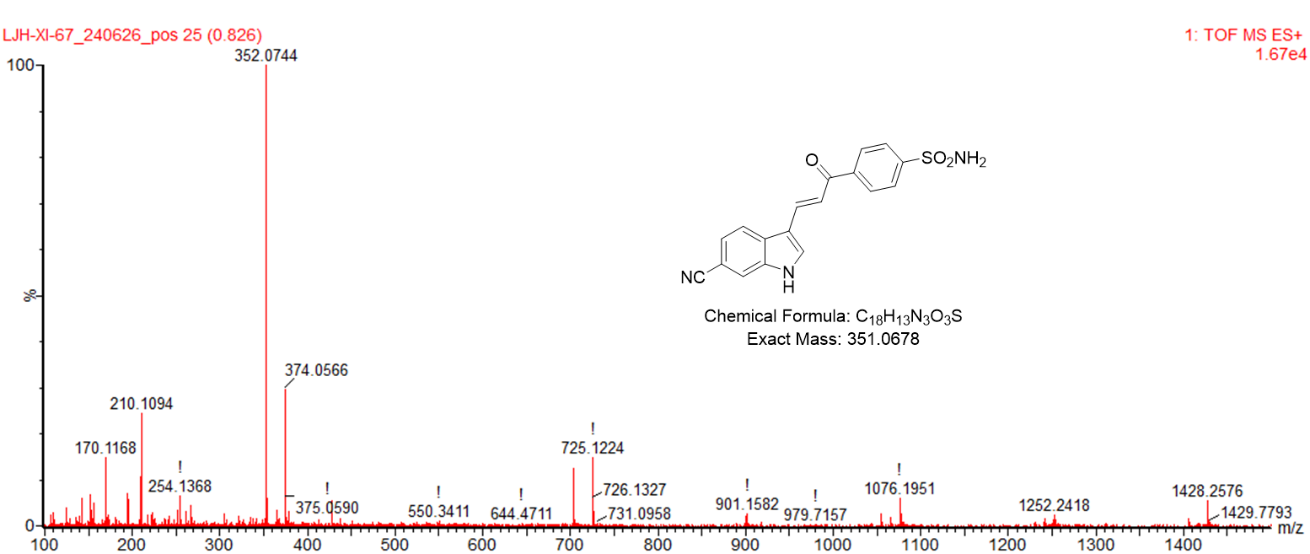


HRMS spectrum of **15k**


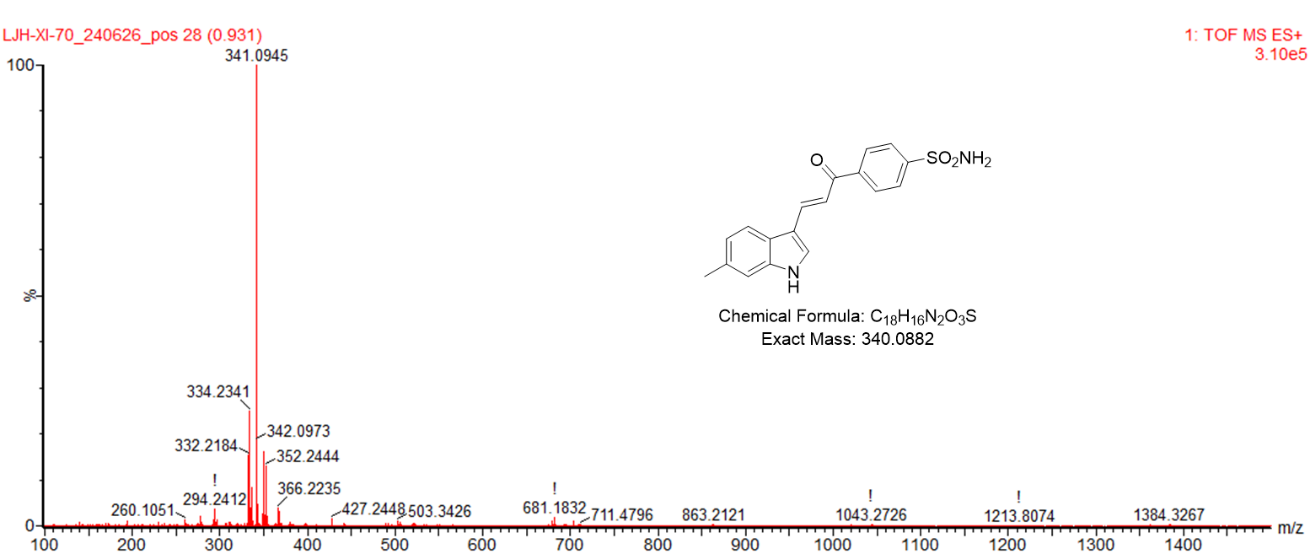


HRMS spectrum of **15l**


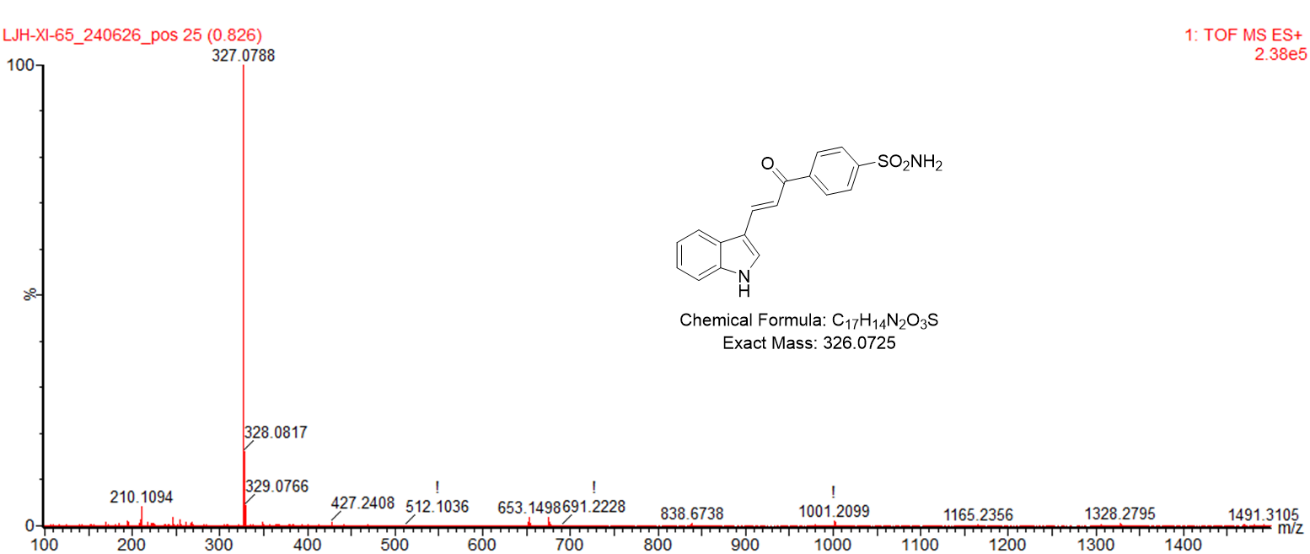


HRMS spectrum of **15m**


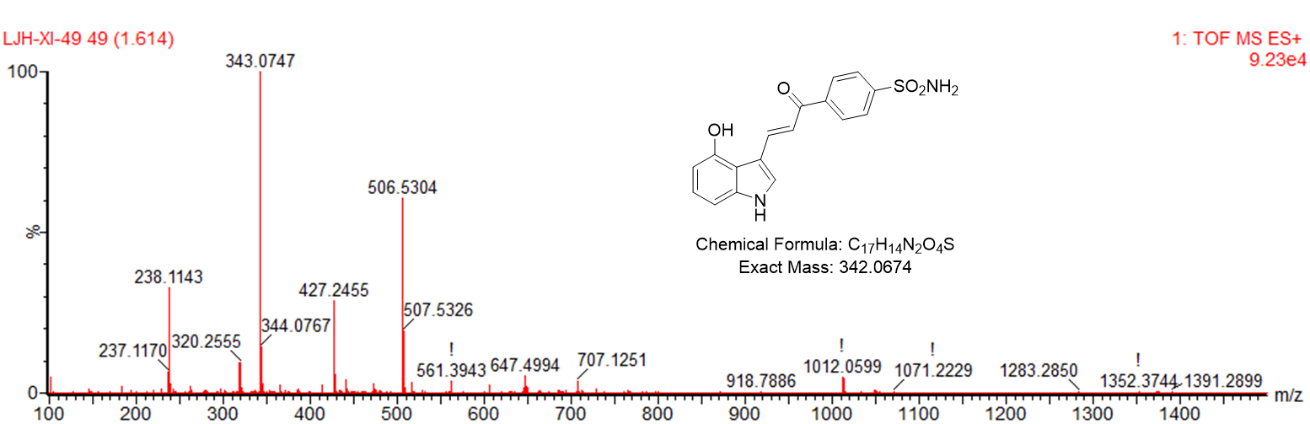


HRMS spectrum of **16a**

**
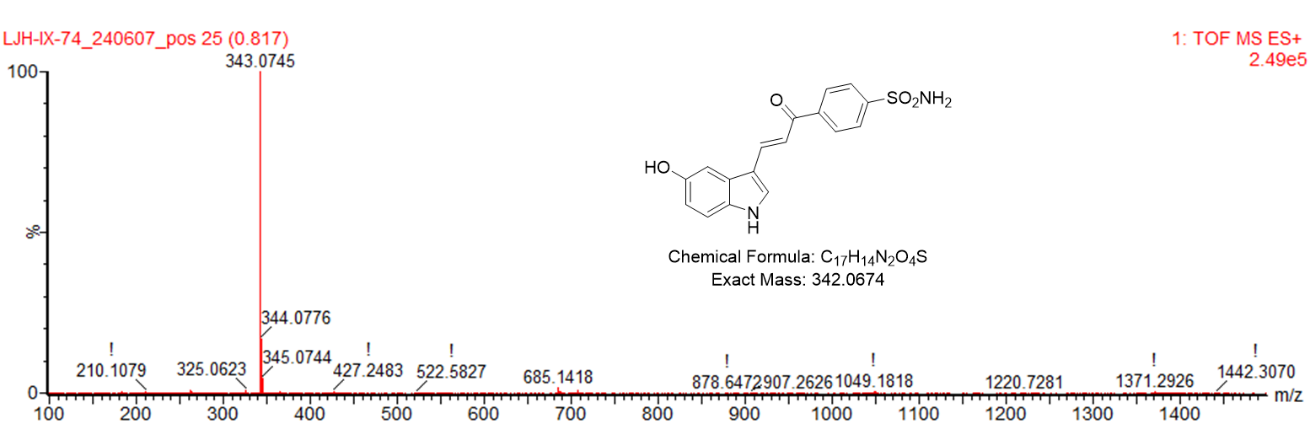
**

HRMS spectrum of **16b**

**
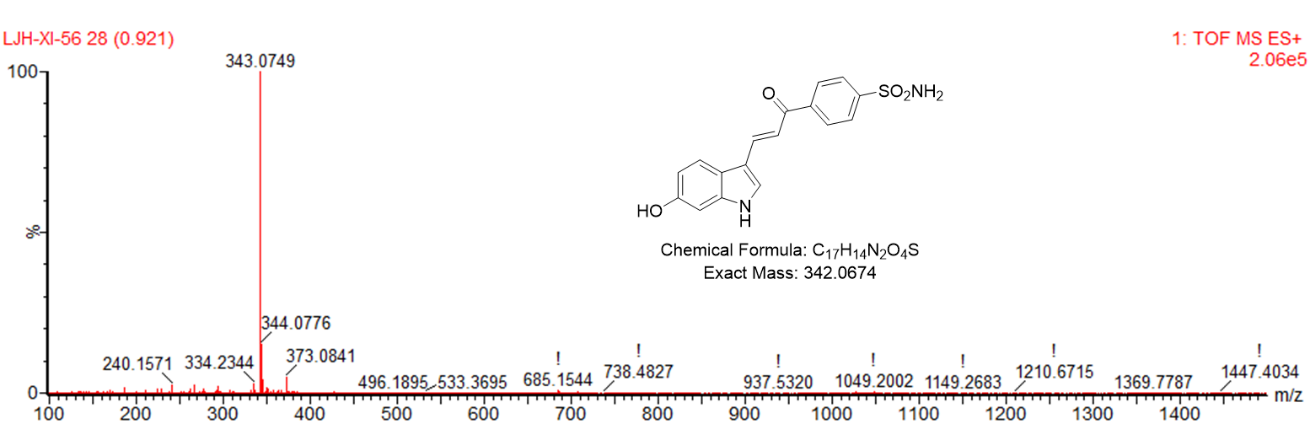
**

HRMS spectrum of **16c**

**
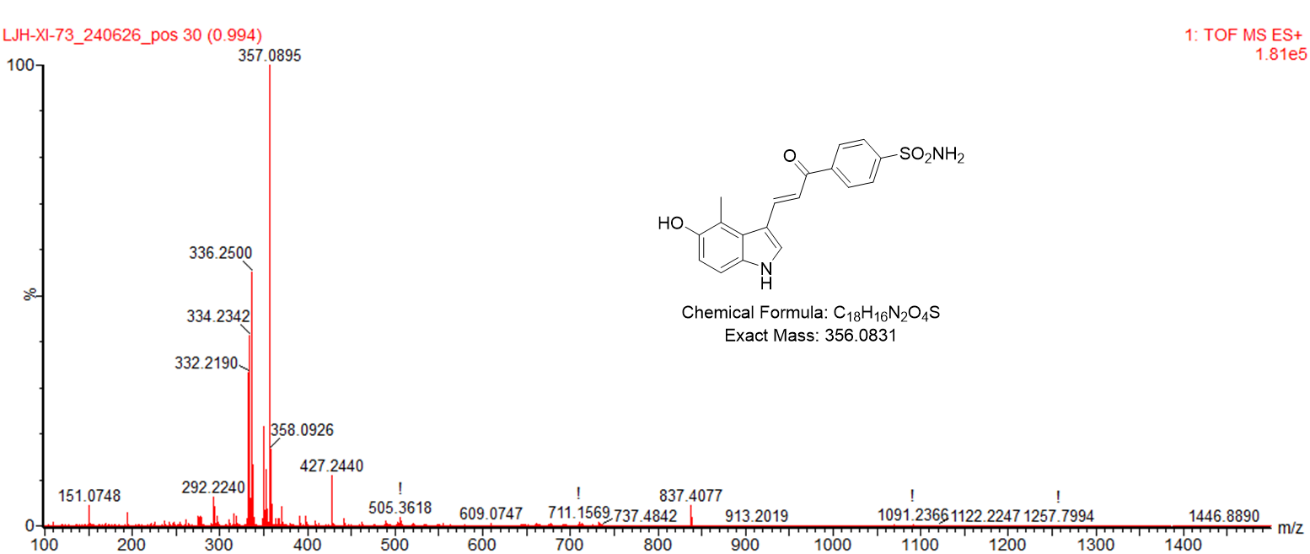
**

HRMS spectrum of **16d**

**4. IC_50_ determination graphs of hCA I, II, IX and XII**

**
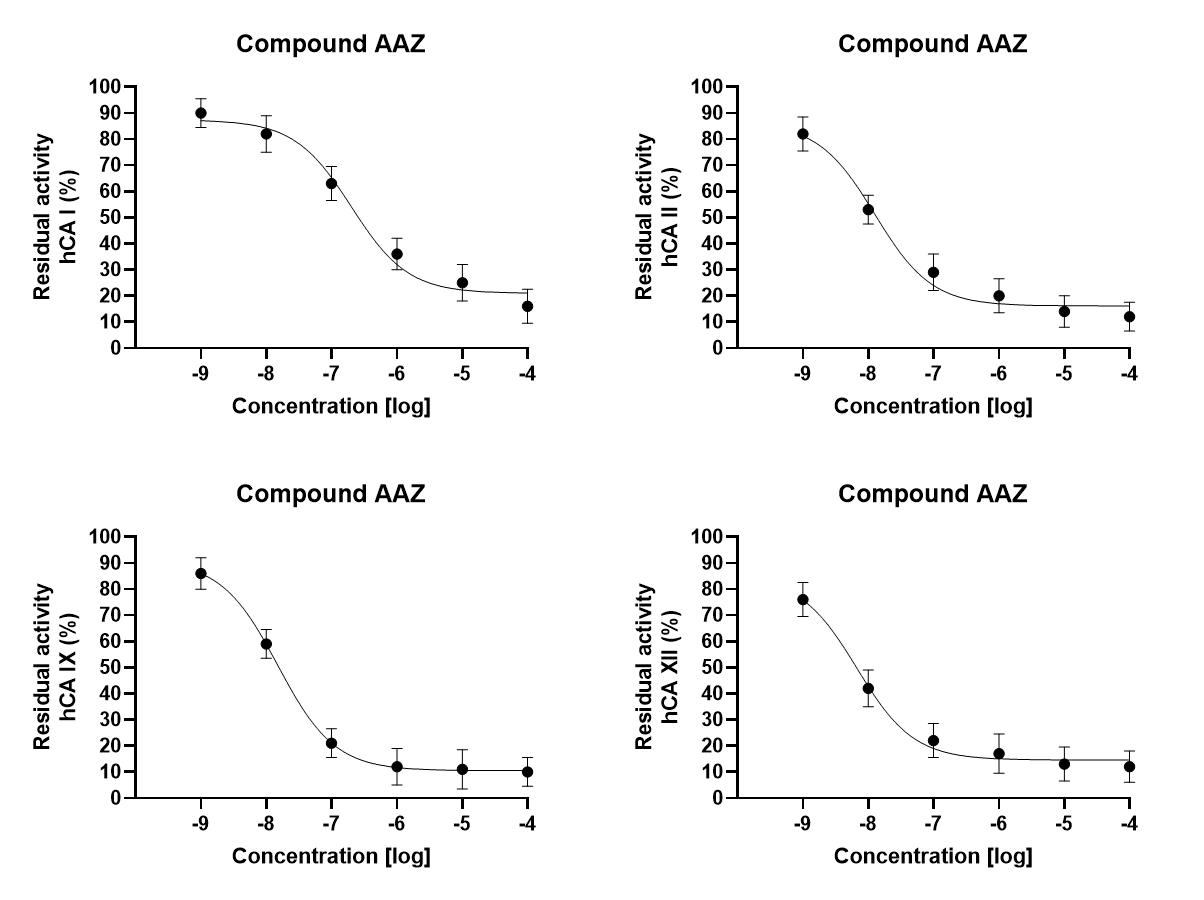
**

IC_50_ determination graphs of hCA I, II, IX and XII for Acetazolamide (AZZ)

**
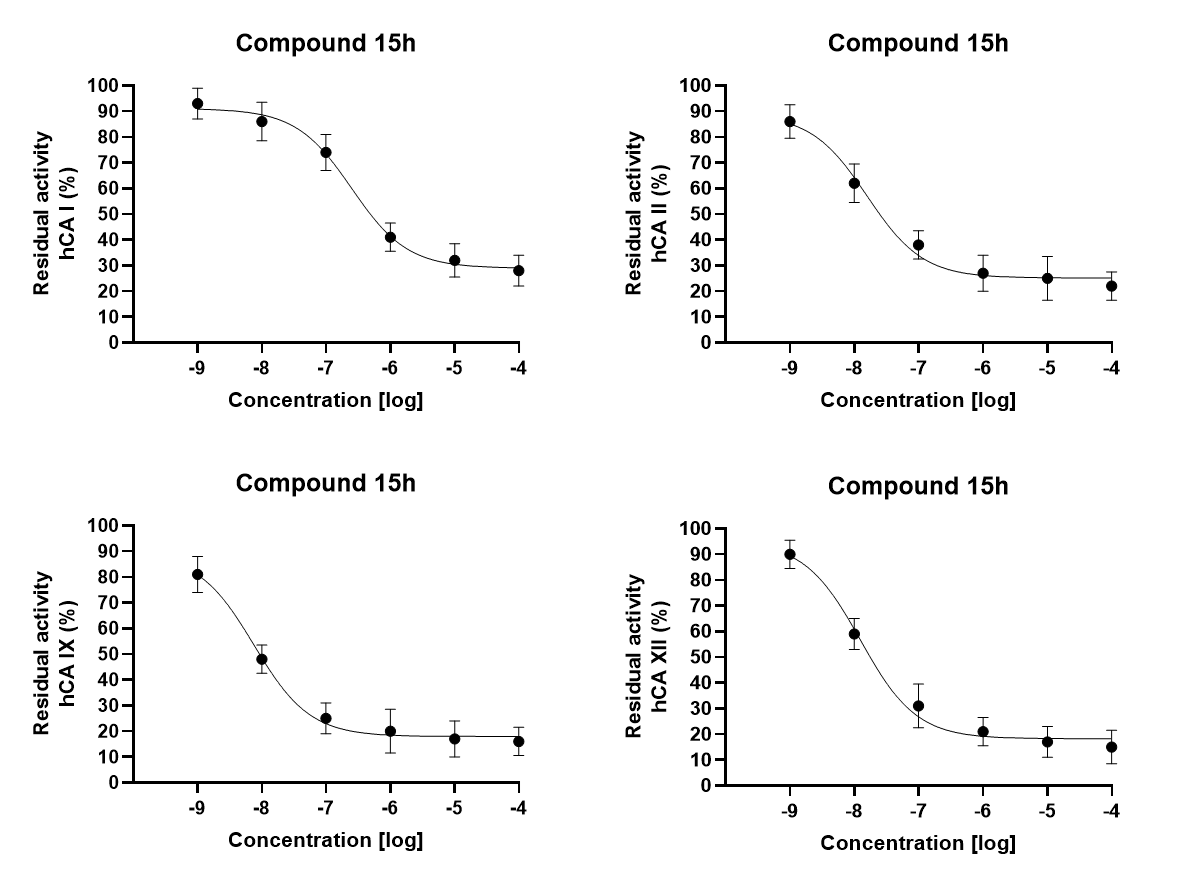
**

IC_50_ determination graphs of hCA I, II, IX and XII for compound **15h**

**
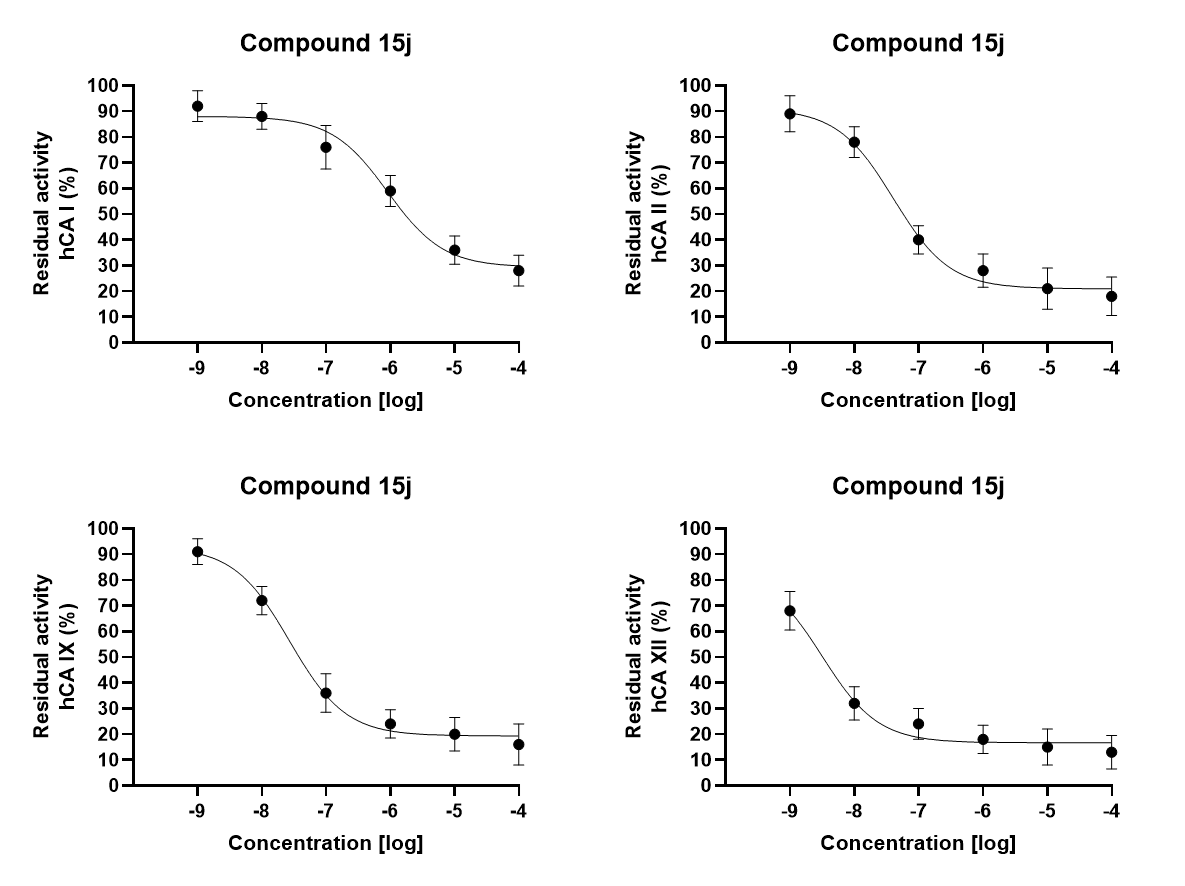
**

IC_50_ determination graphs of hCA I, II, IX and XII for **15j**

**5. Supplementary Figures**


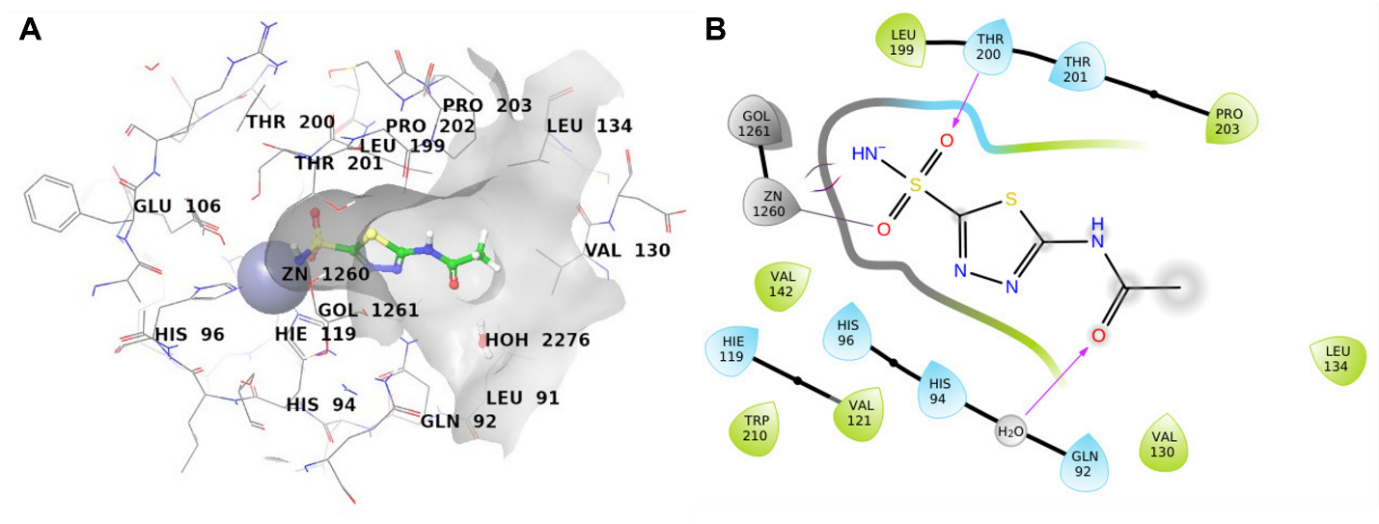


**Figure S1**. Docked complexes of acetazolamide with hCA IX. (a) 3D model of hCA IX complexed with acetazolamide (PDB ID: 5FL5, resolution: 2.05 Å); (b) 2D interaction map of showing the binding interactions of acetazolamide with the active site of hCA IX.


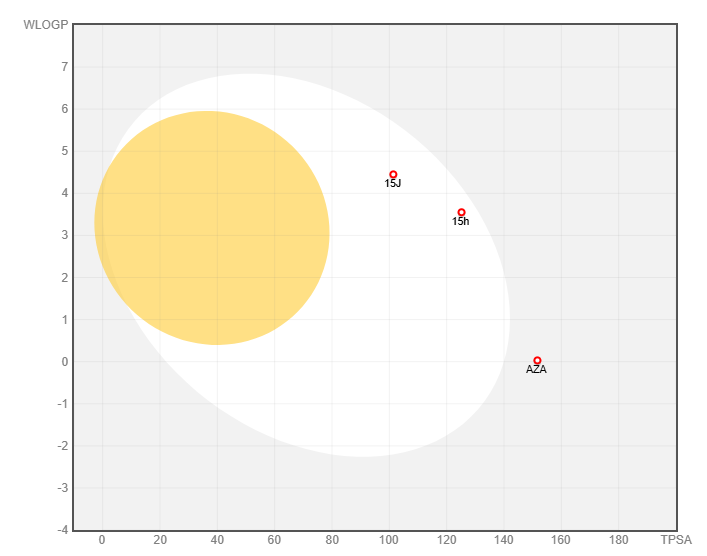


**Figure S2**. Predicted physicochemical property sapce of compounds **15j**, **15h**, and acetazolamide. The yellow region represents compounds predicted to penetrate the blood-brain barrier (BBB), the white region indicates compounds predicted to be passively absorbed into systemic circulation, and the grey region corresponds to compounds predicted to exhibit poor absorption.
